# Applications of wearable sensors in upper extremity MSK conditions: a scoping review

**DOI:** 10.1186/s12984-023-01274-w

**Published:** 2023-11-18

**Authors:** Sohrob Milani Zadeh, Joy MacDermid, James Johnson, Trevor B. Birmingham, Erfan Shafiee

**Affiliations:** 1 Biomedical Engineering, Physical Therapy, Western University, London, ON Canada; 2 Physical Therapy and Surgery, Western University, London, ON Canada; 3 Roth-McFarlane Hand and Upper Limb Centre, St. Joseph’s Health Care, London, ON Canada; 4 School of Rehabilitation Therapy, Queen’s University, Kingston, ON Canada; 5 Clinical Research Lab, Hand and Upper Limb Center, St. Joseph’s Health Center, London, ON Canada; 6 Rehabilitation Science McMaster University, Hamilton, ON Canada

**Keywords:** Wearable sensor, Musculoskeletal condition, Upper extremity, Inertial measurement unit

## Abstract

**Purpose:**

This scoping review uniquely aims to map the current state of the literature on the applications of wearable sensors in people with or at risk of developing upper extremity musculoskeletal (UE-MSK) conditions, considering that MSK conditions or disorders have the highest rate of prevalence among other types of conditions or disorders that contribute to the need for rehabilitation services.

**Materials and methods:**

The preferred reporting items for systematic reviews and meta-analysis (PRISMA) extension for scoping reviews guideline was followed in this scoping review. Two independent authors conducted a systematic search of four databases, including PubMed, Embase, Scopus, and IEEEXplore. We included studies that have applied wearable sensors on people with or at risk of developing UE-MSK condition published after 2010. We extracted study designs, aims, number of participants, sensor placement locations, sensor types, and number, and outcome(s) of interest from the included studies. The overall findings of our scoping review are presented in tables and diagrams to map an overview of the existing applications.

**Results:**

The final review encompassed 80 studies categorized into clinical population (31 studies), workers’ population (31 studies), and general wearable design/performance studies (18 studies). Most were observational, with 2 RCTs in workers’ studies. Clinical studies focused on UE-MSK conditions like rotator cuff tear and arthritis. Workers’ studies involved industrial workers, surgeons, farmers, and at-risk healthy individuals. Wearable sensors were utilized for objective motion assessment, home-based rehabilitation monitoring, daily activity recording, physical risk characterization, and ergonomic assessments. IMU sensors were prevalent in designs (84%), with a minority including sEMG sensors (16%). Assessment applications dominated (80%), while treatment-focused studies constituted 20%. Home-based applicability was noted in 21% of the studies.

**Conclusion:**

Wearable sensor technologies have been increasingly applied to the health care field. These applications include clinical assessments, home-based treatments of MSK disorders, and monitoring of workers’ population in non-standardized areas such as work environments. Assessment-focused studies predominate over treatment studies. Additionally, wearable sensor designs predominantly use IMU sensors, with a subset of studies incorporating sEMG and other sensor types in wearable platforms to capture muscle activity and inertial data for the assessment or rehabilitation of MSK conditions.

**Supplementary Information:**

The online version contains supplementary material available at 10.1186/s12984-023-01274-w.

## Introduction

Wearable motion sensors are light, non-invasive electronic devices [1] that can be comfortably worn or carried by individuals. These systems are designed to assess, monitor, and report specific physical, positional, movement or physiological parameters. They can be used to monitor people providing information arising from different body segments such as the wrist, shoulder, back, chest, knees, and other limbs. Through technological advancements in recent years, various applications of wearable sensors have been suggested and proposed in different fields such as engineering [2], health care [3], gaming [4], daily activity [5], social networking [6], and the military [7]. One of the main applications of wearable sensors in health care studies is the assessment of motion or muscle activity, in patients with various disorders or diseases or healthy individuals. These wearable sensors enable clinicians to have reduced assessment times and obtain objective and quantifiable data of individuals. Sensors may complement the subjective health measurement outcomes derived from clinicians’ and patients’ perspective since they assess different parameters. Moreover, unobtrusive, and continuous measurements can be recorded by using wearable sensors. This enables functional or remote monitoring of patient status in real world daily life activities. Application of this data can support implementation of more affordable or more customized therapy for patients in their home environment, allowing a better and more extensive rehabilitation process [8]. Finally, corrective feedback can be presented to patients by increasing their understanding of performing correct movement patterns during therapy sessions (in the clinic or at home) [9].

Inertial measurement units (IMUs) and surface electromyography sensors (sEMG) are the two leading technologies that are used for the measurement of movement quality across various joints [10, 11]. An IMU usually contains an accelerometer, a gyroscope, and a magnetometer that provides linear accelerations, angular velocities, and strength of magnetic fields in 3 dimensions, respectively [12]. Thus, it can be utilized to assess the body segments’ motions. On the other hand, the sEMG sensor provides a time-stamped signal, frequency, and strength of muscle activity [13]. Therefore, it can obtain more complementary information regarding motor function to therapists and clinicians [8]. Additionally, other types of wearable sensors such as potentiometers and encoders [14, 15] and piezoresistive sensors [16] translate the resistance alterations made by bending sensors or the presence or absence of light made by angular displacement to angles or other motion-related outcomes. However, the applicability of IMUs sensors is increasing rapidly compared to these sensors.

One of the main fields of health care that requires continuous, reliable, and objective clinical measurements or rehabilitation is Musculoskeletal (MSK) disorders. In this regard, 1.71 billion people worldwide have musculoskeletal conditions, which is the highest rate among other types of conditions or disorders that contribute to the need for rehabilitation services [17]. Specifically, Upper extremity musculoskeletal (UE-MSK) disorders and conditions are a significant concern in today’s world since they impose health burdens on patients and a substantial economic burden on society due to sick leave and health care expenses [18]. Although a well-founded and reliable specific global prevalence rate for UE-MSK conditions around the world has not been obtained (due to a lack of a globally accepted definition of UE-MSK disorders or conditions), a conducted study by Huisstede et al. demonstrated that a significant proportion of the MSK disorder population could fit in this category [18]. Therefore, improving the quality of health care, evaluation, and rehabilitation of UE-MSK conditions or disorders will significantly assist the clinicians and patients. In this regard, providing objective, easy-to-use, cheap, and rapid measurements of motion quality can substantially increase the rehabilitation accuracy and diagnosis efficiency and reduce the related costs of therapy sessions. Wearable sensors can be a perfect choice to be applied for assessments of UE-MSK disorders since they can bring forth the mentioned advantages. On the other hand, recent quarantines and health guidelines made by governments and health organizations due to the Covid-19 outbreak worldwide have significantly reduced in-person rehabilitation sessions or therapy visits. This further necessitates the use of wearable sensors for remote and at-home assessments of movement or motion quality.

To date, several reviews have been conducted to assess various types and applications of wearable sensors in rehabilitation or other healthcare fields and evaluate the functionality of these applications. A recent scoping review by Kim et al. has provided the applications of wearable sensors for assessment and treatment of upper extremities (UE) in the population of stroke patients [19]. The reviewed articles in this study applied wearable sensors to obtain UE functional motion, sort motor impairment/activity limitation, augment UE training by providing various types of feedback. Moreover, this review has demonstrated the application of wearable sensors in determining the home-based rehabilitation dosages, the characterization of daily UE use patterns in individuals’ lives, and the rate of adherence to home-based therapy sessions [19]. In another literature review, a similar population and context have been examined by Maceira-Elvira et al. [8]. This literature review has aimed to present an overview of applications of wearable sensors in stroke upper extremity rehabilitation research from different aspects. The study assessed the different wearable sensor technologies in the stroke population, their data processing methods, and instruments. Finally, it has been concluded that aside from the advantages of wearable sensors, IMUs and sEMG sensors offer the best aspects of unobtrusiveness, robustness, user-friendliness, and data quality [8].

Another scoping review study by Sethi et al. has focused on articles investigating the use of inertial motion sensors, sEMG-based, and e-textiles-based interactive wearable technologies [9]. This review summarizes the current applications, limitations, and future of inertial motion and sEMG sensors on different populations such as healthy individuals, stroke patients and neurologically impaired groups. However, it has been mentioned that wearable sensor technologies have encountered certain limitations such as large size equipment, a limited utility for clinical applications, and burdensome setup processes. On the other hand, it has been prospected that through the growth of cloud systems and machine learning algorithms, the data transfer process of these systems will become more convenient [9]. The biofeedback designs for home-based rehabilitation applications have been examined in another scoping review. In this regard, it has been reported that the analyzed feedback introduced in the studies were mainly based on visual, concurrent, and descriptive representations. Moreover, the included articles have investigated the potential reasons for using a feedback system, its user-friendliness, and evaluations [20]. In total, the mentioned reviews have extracted specific parameters of the included articles, such as the number of participants, data collection procedure, sensor type, number, and placement location, data processing methods, assessment type, and results.

Wearable health systems have been reviewed to be applied in clinical practice by Lu et al. [21]. This article has claimed that wearable medical devices have been applied to all parts and limbs of the human body. Furthermore, it has categorized the devices into four application areas: health and safety monitoring, chronic disease management, disease diagnosis/treatment, and rehabilitation. Similarly, this study declares limitations such as the absence of user-friendly solutions, security and privacy concerns, and the lack of industry standards [21].

All these reviews have aimed to provide a map of all available evidence for their research question and highlight the existing gaps in the examined contexts and populations, such as stroke patients or home-based rehabilitation. In contrast to systematic reviews, none of these scoping reviews have conducted a critical appraisal of individual sources of evidence since this step is considered optional for scoping reviews based on PRISMA extension for scoping reviews (PRISMA-ScR checklist and explanation) [22].

Considering the advantages presented by the using wearable sensors in the context of UE-MSK clinical applications, along with the distinct applications of wearable sensor systems in UE-MSK conditions, and the challenges associated with their functionality and usability, this scoping review aims to offer an extensive overview of the most recent findings in related studies. Additionally, we intend to shed light on the prevalent challenges and identify existing gaps within this research field.

In summary, the novelties of our review were covering a broad spectrum of wearable sensor applications in individuals with or at risk of developing UE-MSK conditions, encompassing diverse populations and applications, and categorizing studies into clinical population, workers’ population, and general wearable design/performance studies.

## Materials and methods

In the present scoping review, the outlined guidelines suggested by the preferred reporting items for systematic reviews and meta-analysis (PRISMA) extension for scoping reviews have been considered and applied [22]. The population, concept, context (PCC) structure was selected to identify the critical elements of the research question of this study for conceptualizing purposes. In this regard, the focused populations are patients and individuals who are either diagnosed with UE-MSK disorders (e.g., arthritis or carpal tunnel syndrome patients) or individuals who are at risk of developing UE-MSK conditions (e.g., manufacturing workers, surgeons, farmers), UE wearable sensors (concept), and assessment or rehabilitation (context) [22, 23]. The scoping reviews cannot be registered within the International Prospective Register of Systematic Reviews; however, it has been registered on the Open Science Framework (https://osf.io/8h2mn/).

### Databases and systematic search

Two independent authors (SM and ES) conducted a systematic search of PubMed, Embase, Scopus, and IEEEXplore databases on 4 January 2023. Three categories of keywords and their iterations were used to obtain the relevant articles. The first group of keywords included wearable sensors concept and its iterations such as wearable sensor, wearable electrode, wearable electronic, wearable device, smart prosthesis, electronic textile, IMU, and inertial measurement unit. IMU and its iterations were included in this group since it is the most applied sensor for motion measurements; however, some studies have exploited IMUs and applied a wearable platform but have not mentioned the explicit iterations of the wearable sensor in their script. The second group includes upper extremity, upper limb, shoulder, hand, wrist, elbow, arm, forearm, and finger to obtain the upper limb related articles, and the third group includes the keywords related to the musculoskeletal system such as musculoskeletal, MSK, MSD, muscle, and bone. In Additional file 1: Appendix A, the detailed applied search strategies for each database have been presented. Moreover, the references of included full-text reviewed articles and Google Scholar were examined to search for additional relevant articles. Regarding searching the grey literature, conference abstracts were examined and screened to be included. In case of any disagreement between the two authors regarding the inclusion of a study in the review, the third author (JM) helped to resolve the disagreement.

### Selection criteria

Inclusion criteria:

- Adults with MSK condition or the at-risk of developing an MSK disorder or condition
- Utilizing wearable sensors
- Sensor placement or assessment of outcomes related to shoulder/upper arm, elbow/forearm/wrist, and hand/finger
- Studies published in peer-reviewed journals and conference abstracts with available full text from 2010

Exclusion criteria:

- Wearable sensors applied on robots
- Exoskeletons
- Brain or human–computer interfaces (BCI/HCI)
- Focusing on gait or balance
- Measurements or applications not related to MSK rehabilitation or assessment
- Inadequate details of hardware or measured outcomes of wearable sensors (insufficient details)

The studies providing details regarding the utilized wearable system on people with UE-MSK condition or at risk of developing a UE-MSK condition were included. Due to variability of sensor placements (and in some cases, due to not providing precise sensor placement locations), sensor placement locations have been divided into three regions: shoulder/upper arm, elbow/forearm/wrist, and hand/finger. Furthermore, due to significant technological advancements of wearable sensors in recent years, only the published studies after 2010 have been included in the search process. General wearable design studies and designs for the assessment of muscle activity or limbs motion (not on a specific population) are also included during the search process. This decision has been made to encompass all possible applications of wearable sensors on MSK-population, especially in motion assessments, a typical evaluation in the clinical rehabilitation field.

Regarding the wearability aspect of the applications, the studies that developed a framework for wearability (e.g., embedding sensors in a fabric pocket) were included. Furthermore, studies that mentioned or considered their presented measurement sensors as a wearable system have also been included.

Exoskeletons, BCI/HCI, and other robotic applications were excluded since they are primarily designed for neurological disorders applications and are outside the scope of this study’s research question. Similarly, the studies focusing on gait or balance were excluded. Furthermore, the wearable sensors recording or measuring general physiological parameters (such as blood pressure and heartbeat) unrelated to UE-MSK conditions were also excluded.

All in all, obtained studies through the database search, a manual search of Google Scholar, and conference abstracts were imported into Covidence, a review management software (https://www.covidence.org). Duplicates were removed, and the remaining titles and abstracts were screened. Subsequently, the full-text review process has been performed to find the eligible studies for inclusion in this review. The references of included studies have been examined to find any potentially relevant articles that were not obtained through the search process of databases.

### Data extraction and analysis results

Two independent authors (SM and ES) extracted data on the study designs, study aims, number of participants, sensor placement locations, sensor type and number, outcome(s) of interest, the processing software, and the presence of other features, including home-based applicability, comfortability-assessment, and wireless data transmission ability. We also extracted details on intervention or data collection procedures and the critical points of outcome processing methods of each study.

The studies proposing a novel wearable system for motion assessment that have conducted motion tests on one participant (case-study) or have not conducted any tests on any participants have been categorized as preliminary studies in the study design section.

All the wearable system data corresponding to upper body limbs and segments (shoulder/upper arm, elbow/forearm/wrist, and hand/finger) have been extracted and presented in this review. Several studies might exploit wearable sensors on other body parts such as the neck, back, and knees in addition to upper limbs. Nevertheless, the corresponding information related to neck, back, and lower-body segments has not been reported and summarized. For example, some of the workers’ population studies have used a full-body size wearable system comprising of IMU sensors on lower body parts in addition to upper body regions. Therefore, only corresponding details of hands, wrists, elbows, and shoulder in these certain studies were extracted and analyzed. Subjective outcomes and non-wearable related data are also excluded. Moreover, since the focus of this review is on the applications of wearable sensors, the results and statistical analysis procedures of the included studies have not been included. It must be noted that the full-text script and supplementary materials of all included studies have been reviewed to extract the mentioned parameters. All in all, any potential absence of further details in some of the parameters of studies (e.g., intervention details or software used for processing) is due to the fact that the authors have not reported the corresponding data.

## Results

**Fig. 1:**
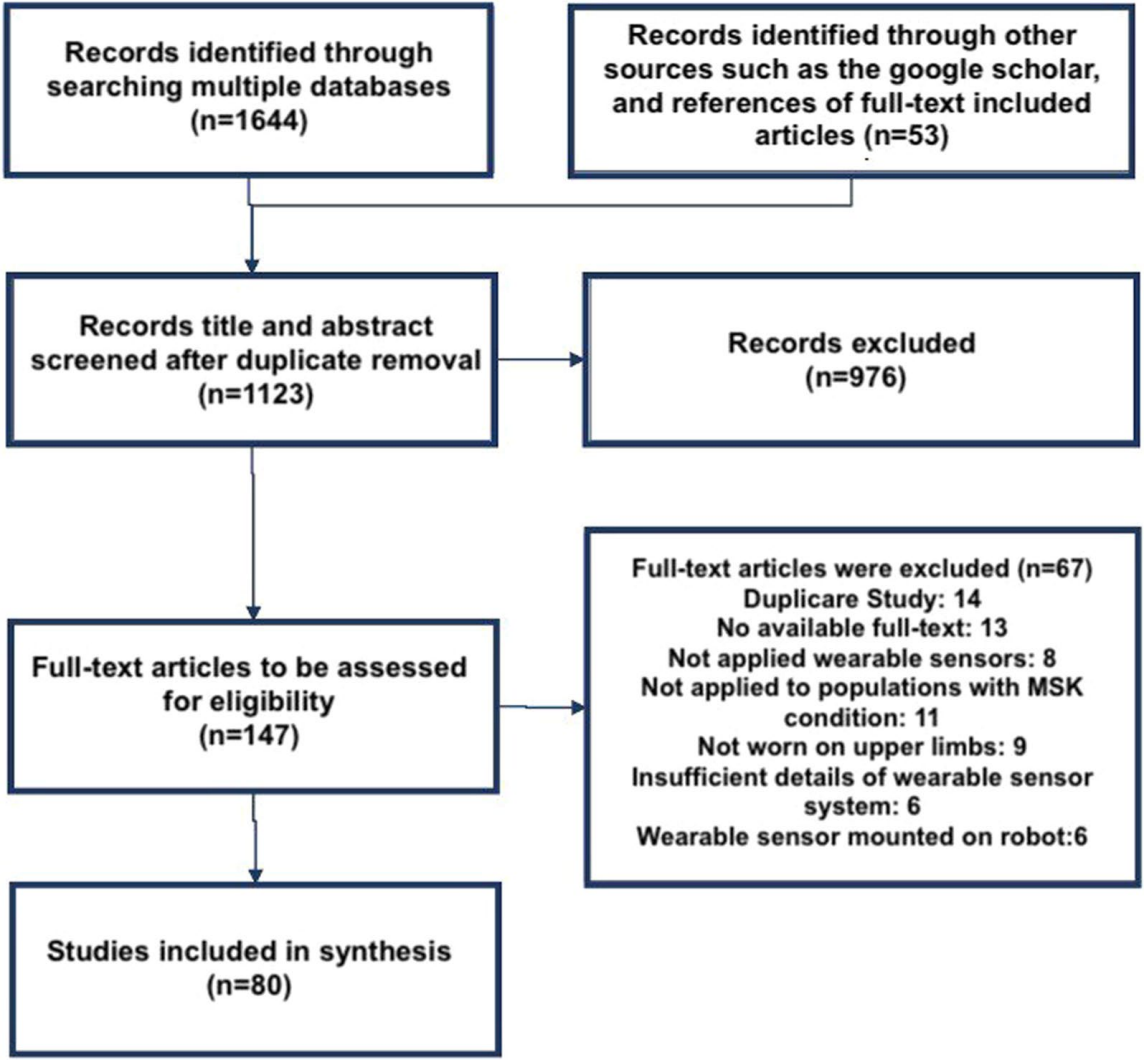
PRISMA flowchart of the screening and review process for included studies

The scoping review search process initially resulted in finding 1644 documents through the 4 databases. Furthermore, 42 studies were also obtained through the Google Scholar. After duplicate removal, 1123 studies were identified for the title and abstract screening stage. Through this process, 976 irrelevant studies were removed, and the full-text reviewing process was initiated. Finally, 67 documents were also removed in this stage, as they did not fit the inclusion criteria or the objective of this review. Hand searching of reference lists led to 11 additional studies at the full-text review phase. This process led to the inclusion and analysis of a total of 80 studies presented in this scoping review. In Fig. 1, the results of searching, screening, eligibility, and inclusion processes have been demonstrated while applying the PRISMA methodology [22].

The synthesis process led to categorizing the included studies into three categories (1) Clinical population studies, (2) Workers’ population studies conditions, and (3) General wearable design/performance studies. The summarized results for each category are presented in the following. Tables 1, 2, 3 provide the extracted detailed information of included studies in each category.

**Table 1:** Clinical population studies

Author year	Participants (Intervention/Control)	Study design type	Aim	Sensor placement	Sensor type, model (provider), utilized number, and wearable platform [core processing unit is included in case of reporting by authors]	Measured outcome(s) [secondary outcomes are indicated with ‘secondary’]	Software for processing/data display	Home- based/ comfortability/ wireless? (✓/×)
(1) Duc et al. 2014 [38]	20 subjects Intervention: 10 patients with rotator cuff tear Control:10 healthy subjects	Cross sectional case control	(1) Quantifying the muscular activation duration in the upper trapezius, medial deltoid, and biceps brachii during movement (2) Investigating the rotator cuff tear impression in both laboratory and daily life settings	Shoulders	(1) 2 IMU sensors of ADXRS and ADXL (triaxial gyroscope, and accelerometer) from Analog devices (Analog devices Inc., USA) (2) 4 EMG sensors (Biometrics® SX230, UK) with sampling frequency of 1.6 kHz	(1) Duration of humorous movements (s) (2) Duration of muscular activation (s) in medial deltoid and biceps brachii	No information	✓/×/×
(2) Pichonnaz et al. 2015 [45]	62 subjects Intervention: 21 patients after rotator cuff surgery Control: 41 healthy subjects	Longitudinal case control [3, 6, and 12 months after surgery]	(1) Assessment of the underuse rate of relevance as an upper limb postsurgical function indicator (2) Applying a new metric to investigate the effect of the rotator cuff surgery on arm usage during the first year after surgery	Shoulders	(1) 2 IMU modules each containing 3 ADXRS gyroscopes and 3 ADXL accelerometers (Analog Devices, Norwood, MA, USA)	(1) Arm activity usage (%)	No information	✓/×/×
(3) Duc. et al. 2013 [46]	Lab phase: 11 subjects Clinical phase: 21 patients after rotator cuff surgery Control: 41 healthy subjects	Longitudinal Case control [3, 6, and 12 months after surgery]	(1) Validating a method using wearable inertial sensors for detecting humerus movements relative to the trunk (2) Providing new outcome parameters for arm movement during long-term measurement of daily activity	Shoulders	(1) 2 IMU modules each containing 3 ADXRS gyroscopes and 3 ADXL accelerometers (Analog Devices, Norwood, MA, USA)	(1) The movement frequency of arms (number of arm movements/ the number of sitting and standing hours) (2) The symmetry index of Fr in dominant and non-dominant arms (%) [Using duration of movements (h) and angular velocity of arms (°/s)]	No information	✓/×/×
(4) Najafi et al. 2021 [50]	29 subjects Intervention: 16 neurogenic thoracic outlet syndrome (nTOS) patients Control: 13 healthy subjects	Cross-sectional case control	(1) Identifying digital biomarkers of upper extremity function impacted by nTOS (2) Using the biomarkers for objectively defining nTOS severity	Upper arm	(1) An IMU system (LEGSys™, BioSensics, Newton, MA, USA) attached using an adjustable strap (sampling frequency = 100 Hz)	(1) Joint angle, and angular velocity in 3 axes (yaw, pitch, roll; °, and °/s) (2) Number of performed cycles (3) Duration of abduction and adduction movements (ms)	MATLAB	×/×/×
(5) Van de Kleut. et al. 2021 [47]	33 subjects indicated for reverse total shoulder arthroplasty (RTSA)	Longitudinal case series [1 month before surgery, and 3-month and 1-year after surgery]	(1) Proposing a new system to monitor and record arm motion and activity at home preoperatively and postoperatively RTSA using portable wearable sensors	Shoulders	(1) 2 IMU sensors (3-Space Data Logger, Yost Labs, Portsmouth, OH, USA, (sampling frequency = 10Hz) fitted in a tight-fitting compression shirt of Nike (Beaverton, OR, USA)	(1) Number of elevation events/h (2) Percentage of time spent within different elevation ranges of 0◦–20◦, 20◦–40◦, 40◦–60◦, 60◦–80◦, 80◦–100◦, and > 100◦ (%) (3) The percentage of occurred elevation events within the elevation ranges (%) (4) Intensity of arm activity (low, moderate, or high) [Using upper arm joint angles in 3 axes (yaw, pitch, roll; °)]	MATLAB	✓/×/×
(6) Kwak et al. 2019 [24]	24 patients with rotator cuff disease	Cross-sectional case series	(1) Measuring motion smoothness–based parameters (motion quality) of the shoulder (2) Proposing new parameters to discriminate between healthy shoulders and shoulders with rotator cuff tear	Wrist	(1) 1 IMU sensor (LogonU, Seoul, Republic of Korea) attached to the wrist using a stretchable fabric	(1) The number of peaks in the sum of angular velocity (2) The peak velocity–to–mean velocity ratio (ratio of the maximum recorded angular velocity of the motion and the mean angular velocity) (3) The number of sign reversals in angular velocity (number of zero crossings) [Using angular velocity and acceleration of arms (°/s, g)]	MATLAB	×/×/×
(7) Larrivée et al. 2019 [25]	38 subjects affected by rotator cuff tendinopathy	Longitudinal case series [1 week before, the same day, 2 and 4 weeks after the corticosteroid injection]	(1) Evaluation and comparison of test–retest reliability and sensitivity to change of clinical assessments of shoulder function to wrist-based inertial measures of shoulder motion (2) determining the acceptability and compliance of using wrist-based wearable sensors	Wrist of affected shoulder	Self-developed system called WIMU-GPS: (1) 1 Wireless IMUs including GPS (SiRFstarIV, 48 Channels, sampled at 1Hz), (inertial data sampling frequency = 50 Hz) embedded in a fabric wrapping around the wrist	(1) Active time; reported as a ratio with total recording time (ratio) (2) Mean activity count (AC) per minute (ratio) sorted in 3 categories of low-intensity (LIA), medium-intensity (MIA), and high-intensity activities (HIA) [Using 3 axes acceleration data (g)]	No information	✓/✓/✓
(8) Burns et al. 2018 [26]	20 Healthy subjects	Cross-sectional case series	(1) Developing and evaluating the potential for performing home shoulder physiotherapy monitoring using a commercial smartwatch and a new classification method	Wrist	(1) Apple Watch (Series 2 & 3, Apple Inc., USA) containing 6-axis acceleration and gyroscope (sampling frequency = 50 Hz)	(1) Acceleration and rotational velocity in all 3 axes (yaw, pitch, roll; g, and °/s)	PowerSense app	✓/×/✓
(9) Burns et al. 2020 [27]	42 patients with rotator cuff pathology	Longitudinal case series [monthly visits for a year after treatment]	(1) Validity assessment of a wearable sensor system for evaluating participation and technique adherence of shoulder exercise (2) Quantifying the rate of home physiotherapy adherence, and its effect on recovery (3) Designing a pilot test for an ethically conscious adherence-driven rehabilitation program specialized for each individual	Wrist	(1) A Huawei 2 smartwatch (Huawei Technologies Co Ltd, China, sampling frequency = 50 Hz)	(1) Participation adherence rate (%) over each 2-week interval of treatment (2) shoulder active range of motion in 3 axes (yaw, pitch, roll; °)	No information	✓/×/✓
(10) Hurd et al. 2018 [28]	14 subjects undergoing Reverse Shoulder Arthroplasty (RSA)	Longitudinal case series	(1) Evaluating the changes in pain, self-reported function, and limb activity and their correlation and difference after RSA using subjective and wearable sensor-measured outcomes	Wrist and upper arm	2 Triaxial GT3XP-BTLE IMUs (ActiGraph, Pensacola, Florida, USA, sampling frequency = 100 Hz) attached with Velcro straps	(1) Mean limb activity (m/s^2^/epoch) (2) The activity frequency categorized in 3 levels of inactive, low, and high (%) [Using 3 axes accelerometer data (g)]	MATLAB	✓/×/×
(11) Ajcevic et al. 2020 [51]	13 subjects Intervention: 6 subjects with adhesive capsulitis (frozen shoulder) Control: 7 healthy subjects	Longitudinal case control [at the beginning of study and after 3 months]	(1) Investigating the possibility of quantitative evaluation of capsulate-related deficit versus healthy controls (2) Assessment of treatment efficacy by measurement of shoulder kinematic parameters using IMUs	Shoulder (scapula and humerus)	2 MTw wireless IMUs (Xsens Technologies, Netherlands) placed on an elastic cuff	(1) The range of motion in elevation, and abduction movements of scapula and humerus (yaw, and pitch; °) (2) Activation time of the scapula and humerus (s)	No information	×/×/×
(12) Chen et al. 2020 [44]	Reliability: 25 subjects [ 10 for control group and 15 with Adhesive Capsulitis Effectiveness: 15 subjects [7 for home-based exercise and 8 for motion sensor–assisted]	Longitudinal case control [at the beginning of study and after 1, 2, and 3 months]	(1) Verifying the reliability and effectiveness of a treatment model using a wearable motion sensor device to assist AC patients by performing home-based exercises to improve training compliance and the accuracy of exercises	Upper arm and Wrist	(1) 1 Motion sensor device (BoostFix wearable self-training kit, COMPAL Electronics Inc, Taipei, Taiwan) containing 6-axis microelectromechanical systems attached to body parts with straps	(1) Active ROM and passive ROM of shoulder (yaw, pitch, roll; °) (2) Exercise completion rates (%)	Doctor and patient apps developed by BoostFix	✓/×/×
(13) Aslani et al. 2018 [39]	7 Subjects [6 healthy subjects and 1 subject with frozen shoulder]	Cross-sectional case series	(1) Developing and evaluating a single IMU accompanied by an EMG sensor for monitoring the 3D reachable workspace along with simultaneous measurement of deltoid muscle activity	Upper arm	(1) IMU sensor: 1 BNO055 (Adafruit, USA) attached with an adjustable band (2) EMG sensor: MyoWare Muscle Sensor	(1) Shoulder range of motion in Spherical coordinate (azimuthal angle and elevation angle) [Reference point: Top shoulder joint] (2) RMS values of EMG amplitude (V)	MATLAB	×/×/✓
(14) Yin and Xu-2018 [29]	1 Healthy subject	Preliminary study	(1) Proposing a system to recognize the movements of patients that require upper limb rehabilitation like frozen shoulder patients	(1) Upper arms, forearms, and hands	(1) 6 IMU sensors embedded in a designed stretchable sleeve (No further details)	(1) Joint elevation angles in all 3 axes (yaw, pitch, roll; °)	Self-developed game through Microsoft® XNA game engine	✓/×/✓
(15) Xuedan et al. 2019 [42]	No information	Preliminary study	(1) Proposing new treatment of shoulder periarthritis using near-infrared light through stimulation of the acupuncture points on frozen shoulder	Shoulder (scapulohumeral area)	(1) High-power LEDs (800mW) with wavelength of 940nm (near infrared) mounted on flexible circuit boards were attached to a mesh vest by a nylon Velcro (voltage:1.4–1.7 V; current:700–1000 mA)	(1) Peak optical power (W) (2) peak optical density (mW/cm^2^)	Self-developed software	×/×/×
(16) Körver et al. 2014 [52]	Control: 100 healthy subjects Intervention: 15 patients with subacromial impingement syndrome	Longitudinal case control [at diagnosis and 5 years after diagnosis]	(1) Objective assessment of shoulder movements in patients with subacromial impingement syndrome at baseline and at five-year after treatment using inertial movement sensor measurements	Upper arm	(1) 1 Inertia-Link-2400-SK1 IMU sensor (MicroStrain, Inc., Williston, Vermont, USA) fixed with an adhesive patch	Upper arm accelerations and angular velocity or rate in 3 axes (yaw, pitch, roll; g, and °/s)	3DM-GX2 Software Development Kit	×/×/×
(17) Lorussi et al. 2019 [43]	10 Healthy subjects	Cross-sectional case series	(1) Development of a shoulder physiotherapy application (called ShoulPhy) for shoulder impingement syndrome patients with the aims of (a) remote monitoring of the subject’s adherence to the program (b) providing a quantitative evaluation of the therapeutic activity and functional level (c) designing individualized exercises by the therapists	IMUs: Wrists Strain: spine to shoulder (scapula)	(1) 2 IMUs (MTw; XSens, Netherlands) embedded in wearable straps; 1 textile strain sensor (optical detection of relative positions of markers is made by Smart DX 100 (BTS Bioengineering, USA)	(1) Shoulder and wrists joint angles in 3 axes (yaw, pitch, roll; °) with respect to standing position reference (2) The measurements error of software in 3 axes (yaw, pitch, roll; °)	ShoulPhy (self-developed app)	✓/×/×
(18) Carmona-Ortiz et al. 2020 [53]	A healthy subject and a subject with Becker muscular dystrophy	Cross-sectional case series	(1) The development of a fully portable, nonobtrusive, and wearable IMU-based motion measurement system called the ArmTracker tracking arm and torso kinematics during daily life	Upper arms and forearms	(1) 4 IMU sensors (BNO055, Bosch Sensortec GmbH, sampling frequency = 50 Hz) encapsulated in a 3D printed plastic case (2) a microcontroller (Teensy 3.6, PJRC.COM, LLC.) [All components are embedded in a Lycra shirt.]	(1) Arm and wrists joint angles and acceleration (yaw, pitch, roll; °, g)	MATLAB	✓/×/×
(19) Zucchi et al. 2020 [30]	40 subjects with distal radius fractures [20 treated with Kirschner wire fixation and 20 subjects treated with volar plate fixation]	Cross-sectional case control	(1) Retrospective evaluation of wrist ROM for patients in recovery after Kirschner wire fixation and volar plate fixation surgical treatment, using an IMU thereby evaluating the presence of compensatory movements (2) Assessment of the presence of muscle fatigue, through sEMG	IMU: hand EMG: upper arm	(1) IMU: a single IMU sensor (Fisiocomputer, Rome, Italy) attached to hand with a band (2) sEMG: a multichannel Pocket Free EMG system (BTSengineering, USA, sampling frequency = 1000 Hz)	(1) Affected wrist ROM (°) and in percentages with respect to the unaffected wrist (%) through ulnar and radial deviation (yaw), flexion and extension (pitch), and pronation and supination of forearm (2) EMG amplitude (V)	Fisiocomputer, and BTSengineering software	×/×/×
(20) Perraudin et al. 2018 [31]	Intervention: 30 patients with osteoarthritis, rheumatoid arthritis, or psoriatic arthritis Control: 15 healthy subjects	Cross-sectional case control	(1) The feasibility of performing unsupervised, correct, and consistent sit to stand tests at home (2) Finding a model that can that demonstrate the relationship of the tests duration obtained from sensors to pain and stiffness	Wrist	1 ActiGraph GT9X IMU sensor (ActiGraph Inc., USA, sampling frequency = 30 Hz) mounted on a wristband	(1) The 3-axis accelerometer data (g), and its spherical coordinates (norm vector, azimuth in degrees, elevation in degrees) (2) The duration of the 5 × STS tests (s) (3) Adherence to the tests (%)	Self-developed smartphone app	✓/×/×
(21) Kassanos et al. 2019 [32]	No information	Preliminary study	(1) proposing a new design for (a) temperature sensing and heating capabilities for localized temperature measurements (b) thermotherapy and closed-loop thermoregulation for the arthritis patients	Wrist	(1) A flexible printed circuit (FPC) with a 50 μm thick, 26 mm wide and 188 mm long, and made from polyimide substrate (wrapped around the wrist)	Temperature of element placed on wrist (°c)	No information	×/×/×
(22) Murad et al. 2017 [33]	1 Healthy subject	Preliminary study	(1) Providing rehabilitation programs through music therapy for individuals with motor impairments using Motion Initiated Music Ensemble with Sensors (MIMES)	Wrist	(1) A smartwatch (no further details)	(1) 3 Axes accelerometer signals of wrist (yaw, pitch, roll; g)	No information	✓/×/✓
(23) Holland et al. 2020 [34]	Control: 17 healthy subjects Intervention: 10 patients with hand arthritis	Cross sectional case control	(1) Evaluating the appropriateness of ‘‘arthritic’’ designed golf grips for patients with hand arthritis by assessment of total applied grip force and grip configuration (ROM) using 12 ‘‘arthritic’’ designed golf grips in golfers with and without hand arthritis	Force: fingers (tips of thumb, index, middle, and ring fingers)	(1) FingerTPS system containing 3 sensors (Pressure Profile Systems, Los Angeles, CA, USA, sampling frequency = 50Hz)	(1) Forces at the distal palmar aspect of the thumb, index, middle, and ring finger of each participant’s trail hand (Pounds per square inch or psi) (2) Grip configuration or ROM of the thumb and index fingers (°)	Dartfish Movement Analysis Software/ Chameleon Visualization Software	×/×/×
(24) Silişteanu et al. 2016 [36]	25 subjects with the Carpal tunnel syndrome	Longitudinal case series [followed by a 30-day duration]	(1) Limiting the flexion/extension movement at the level of hand and fingers by using sensor gloves reducing the recovery time of Carpal tunnel syndrome	Hand	(1) VPL Data Glove (Sun Microsystems Inc., USA) containing 14 optical sensors embedded in a fabric glove	(Secondary) (1) The positions of fingers (no information regarding the details)	No information	×/×/×
(25) Connolly et al. 2018 [54]	9 Subjects with significant (not severe) pain in their hands	Cross-sectional case series	(1) Development of a novel wireless smart glove called iSEG-Glove to facilitate objective accurate measurement of fingers joint movement	Fingers and hand	(1) iSEG-Glove system containing: (a) 16 9-axes IMU sensors; MPU-9150 (TDK InvenSense, Japan) (b) AVR32; UC3C 32 Bit Microcontroller	(1) ROM and angular velocity of finger joints (yaw, pitch, roll; °, °/s)	Self-developed GUI	×/×/✓
(26) Mack and min-2019 [37]	No information	Preliminary study	(1) Developing a wireless wearable wrist positions detection system that monitors symptoms of Carpal Tunnel Syndrome using flex sensors	Wrist	(1) 2 Capacitive resistance flex sensor and potentiometer (Spectra symbol, USA) embedded in a thin fabric glove (2) Arduino Pro Mini; ATmega328 AVR microcontroller	Wrist angle (pitch, °)	MATLAB (Self-developed GUI)	×/×/✓
(27) O’Quigley et al. 2014 [35]	No information	Preliminary study	(1) Proposing a glove for home-monitoring of Rheumatoid Arthritis (RA) patients through assessment of finger joints movement (2) Comparison of the proposed developed sensor glove based on piezo-resistive fabrics with a motion capture VICON Nexus system	Piezo-resistive: Fingers IMU: top of the hand	(1) 10 piezo-resistive sensor fabrics (LR and LTT of Eeonyx, USA), and 1 IMU sensor (No further details) embedded in a glove (2) Arduino Fio as the core processing unit	Finger joint angles (°)	Self-developed GUI	✓/×/✓
(28) Langohr et al. 2018 [48]	36 Subjects undergone shoulder arthroplasty Control: the contra- lateral asymptomatic joint of subjects	Cross sectional case control	(1) Determining the total daily shoulder motion of patients following TSA and RTSA (2) Comparing the mobility of the arthroplasty shoulder with the asymptomatic joint (3) Comparing the daily motion of TSA and RTSA shoulders	Upper arms, forearms, and torso	(1) 5 9-axes IMU sensors (YEI Technology, Portsmouth, OH, USA) embedded in sewn pockets of a stretchable shirt (2) An external battery pack affixed to a belt (3) a tight-fitting long-sleeved spandex shirt (Nike, Beaverton, OR, USA)	(1) ROM and shoulder joint angles (°) (2) Percentage of time spent in different angle ranges of elevation and plane of elevation axes (%) (3) Number of motions per hour in each angle range	LabVIEW (Self-developed GUI)	✓/×/×
(29) Haverstock et al. 2020 [49]	Control: 13 healthy subjects Intervention: 33 Subjects undergone shoulder arthroplasties	Cross sectional case control	(1) Determining the posture and cumulative elbow motion during one-day daily activities (2) Comparing elbow motions of both dominant and nondominant arms	Upper arms, forearms, and torso	(1) 5 9-axes IMU sensors (YEI Technology, Portsmouth, OH, USA) embedded in sewn pockets of a stretchable shirt (2) An external battery pack affixed to a belt (3) a tight-fitting long-sleeved spandex shirt (Nike, Beaverton, OR, USA)	(1) ROM and forearm joint angles in flexion/extension and pronation/supination postures (°) (2) Percentage of time spent in different angle ranges (%) (3) Number of elbow motions per hour in each angle range	LabVIEW (Self-developed GUI)	✓/×/×
(30) Lavado and Vela 2022 [40]	5 Healthy subjects	Cross-sectional case series	(1) Design and implementation of an IMU- and EMG-based wearable device for telemonitoring the elbow flexion- extension angle and muscle activity of patients’ rehabilitation	IMU: Forearm EMG: Upper arm (biceps brachii and triceps brachii)	(1) Gravity Analog EMG sensor (OYMotion, China) (2) MPU-6050 (InvenSense Inc, USA); a 3-axis gyroscope and a 3-axis accelerometer (3) Arduino Nano as the core processing unit	(1) ROM and flexion–extension elbow joint angle (°) (2) EMG amplitude of biceps brachii and triceps brachii (V)	MATLAB (Self-developed GUI)	×/×/✓
(31) Rigozzi et al. 2022 [41]	4 Healthy tennis players	Cross-sectional case series	(1) Developing a novel microcontroller-based wearable device to measure grip strength, forearm EMG activity and vibrational transfer aiding the diagnose of elbow tendinopathy (2) Comparing the grip strength and forearm EMG activity of tennis players with different levels of experience	EMG: Forearm (extensor carpi radialis brevis and flexor carpi radialis) Accelerometer: The racket, wrist (lateral epicondyle of the distal ulnar head) and elbow (lateral epicondyle of the humerus)	A prototype called TRAM-2 attached to the handle of racket: (1) 2 EMG Muscle Sensors (MyoWare AT-04–001, Advancer Technologies, Raleigh, USA) (2) 3 accelerometer sensors (ADXL377, SparkFun, Colorado, USA) (3) a custom-built pressure sensor (Adafruit Velostat and Adafruit Copper Foil Sheet) (4) a microcontroller (Teensy 3.6, PJRC, Oregon, USA)	(1) Normalized EMG activity and grip strength to the maximum voluntary contraction (MVC) activity level (%) (2) Angular rotation of racket, wrist, and elbow (°/s)	No information	×/×/×

ROM: Range of motion; sEMG: Surface electromyography; EMG: Electromyography; IMU; Inertial Measurement Unit; GPS: Global Positioning System; TSA: Total Shoulder Arthroplasty; RTSA: Reverse Total Shoulder Arthroplasty; RSA: Reverse Shoulder Arthroplasty; RMS: Root Measn Square; LED: Light Emitting Diode; RA: Rheumatoid Arthritis; MVC: Maximum Voluntary Contraction; GUI: Graphical User Interface; 3D: Three dimensional; AVR: Advanced Virtual RISC; V: Volt; W: Watt; s: Second; ms: Millisecond; °: Degree; m: Meter; cm: Centimeter; °c: Degree Celsius; Hz: Hertz; g: g-force [Unit of acceleration]

**Table 2:** Workers’ population studies

Author year	Participants (Intervention/Control)	Study design type	Aim	Sensor placement	Sensor type, model (provider), utilized number, and wearable platform [core processing unit is included in case of reporting by authors]	Measured outcome(s) [secondary outcomes are indicated with ‘secondary’]	Software for processing/data display	Comfortability/ wireless? (✓/×)
(1) Humadi et al. 2020 [67]	10 Healthy workers	Cross-sectional case series	(1) Investigating the validity and repeatability of an IMU system for in-field Rapid Upper Limb Assessment (RULA) score assessment in manual handling tasks using 3D Cardan angles and 2D projection angles with respect to values of a motion-capture camera system (reference)	IMU: shoulders, forearms, and wrists Motion markers: Shoulders (scapula and humerus), and forearms	(1) 6 IMUs (MTw, Xsens Technologies, the Netherlands, sampling frequency = 60Hz) (2) Arduino microcontroller (3) A motion-capture camera system (VICON, Oxford Metrics Group, UK) with eight cameras (sampling frequency = 100Hz)	(1) Root-mean-square error (RMSE) of obtained joint angles (°) (2) RULA score (1 to 7) [Using 3 axes upper arm, lower arm and elbow joint angles (°)]	Self-developed software/ Xsens-MVN Analyze	×/✓
(2) Humadi et al. 2021 [68]	11 Healthy novice workers	Cross-sectional case series	(1) Investigating the accuracy and reliability of (a) IMU-based wearable technology and (b) a marker-less optical technology (using Kinect V2) against VICON motion-capture system (the gold standard) based on RULA risk assessment tool during different manual material handling tasks	IMUs: Upper arms, forearms, and hands VICON markers: shoulders, elbows, and wrists	(1) 6 IMUs (MTw, Xsens Technologies, the Netherlands, sampling frequency = 60 Hz) fixed with plates and attached with using double-sided medical tape and extra layers of surgical tape (2) Kinect V2 (Microsoft Corporation, USA) (3) Motion-capture camera system of VICON (Oxford Metrics Group, UK) with eight cameras (sampling frequency = 100 Hz)	(1) Median of joint angles in 3 axes (yaw, pitch, roll; °) (2) Root-mean-square error (RMSE) of obtained joint angles in IMU and Kinect methods with respect to VICON system as the gold standard (°)	Self-developed/ Xsens-MVN Analyze	×/✓
(3) Lee et al. 2020 [75]	4 Healthy observers/ subjects	Cross-sectional case series	(1) Testing the interrater reliability of the IMU-based posture-matching method, for providing applications for the IMU sensor-based motion tracking system (2) Identifying the risk of WMSD	Upper arms, wrists, and hands	(1) 3 I2M IMU Motion tracking system (NexGen Ergonomics Inc, Quebec, Canada, sampling frequency = 128 Hz)	(1) 3 Axes elbow, wrist, forearm, and upper arm joint angles (yaw, pitch, roll; °)	TK Motion Manager/ Human Motion Analyzer	×/×
(4) Akanmu, and Olyawela-2020 [80]	1 Healthy carpenter	Preliminary study	(1) Examining ergonomic exposures of carpentry subtasks that might lead to musculoskeletal injuries (2) Evaluation of preventive and protective interventions impact on the musculoskeletal injuries	Upper arm, and forearm	(1) 2 smartphones (Sampling frequency = 10Hz) attached with straps and bands	(1) Joint angles (yaw, pitch, roll; °) (2) Duration of carpentry subtasks (s)	No information	×/✓
(5) Ohlendorf et al. 2020 [55]	20 Dentist teams and a student control group	Cross- sectional case control	(1) Contributing information on the prevalence of MSD in dental professionals using online questionnaires (2) Ergonomic risk assessment of treatment procedures based on RULA, focusing on treatment concepts of general dentistry, endodontology, oral surgery and orthodontics	Shoulders (scapula and humerus), and forearms	(1) 8 IMU sensors embedded in a Lycra body suite (MVN Link, XSens, Enschede, Netherlands, sampling frequency = 240Hz)	(Secondary) (1) A risk score for every recorded frame using the RULA (a mean score and SD) for wrist and arm (2) The percentage of spent time in the respective risk scores and at high-risk postures (%)	MATLAB/ Xsens-MVN Analyze	×/✓
(6) Blume et al. 2021 [56]	15 Teams consisting of a dental student a dental assistant trainee	Cross- sectional case series	(1) Investigating the ergonomic risk of dental students based on RULA score using inertial sensors during performing standardized dental activities	Upper arms (scapula and humerus), forearms, and wrists	(1) 8 IMU sensors embedded in a Lycra body suite (MVN Link, XSens, Enschede, Netherlands, sampling frequency = 240Hz)	(1) Modified RULA score (1 to 7) and relative time spent at each RULA score [Using accelerometer, angular velocity, and joint angle values in 3 axes (yaw, pitch, roll; °, °/s, g)]	Xsens-MVN Analyze	×/✓
(7) Maurer-Grubinger et al. 2021 [57]	15 Healthy oral and maxillofacial surgeons	Cross- sectional case series	(1) Developing a script that allows quantification of RULA ergonomic risk in dentistry using IMU measured data of joint angles and positions of body segments (2) Proposing various modified RULA outcomes	Upper arms (scapula and humerus), forearms, and wrists	(1) 8 IMU sensors embedded in a Lycra body suite (MVN Link, XSens, Enschede, Netherlands, sampling frequency = 240Hz)	(1) Modified RULA score (1 to 7) and relative time spent at each RULA score [Using accelerometer, angular velocity, and joint angle values in 3 axes (yaw, pitch, roll; °, °/s, g)]	Xsens-MVN Analyze	×/✓
(8) Schall et al. 2021 [60]	35 Subjects [18 manufacturing cyclic workers and 17 non-cyclic workers]	Randomized clinical trial	(1) Comparing mean levels of full-shift exposure summary metrics based on both posture and movement speed between manufacturing cyclic and non-cyclic workers (2) Exploring the patterns of between- and within-worker exposure variance and between-minute (within-shift) exposure level and variation within each group	Upper arms and dominant wrist	(1) 3 ActiGraph GT9X Link IMUs (ActiGraph, USA, sampling frequency = 100 Hz) fixed with elastic hook-and-loop fastener straps using hypoallergenic cohesive bandages	(1) Upper arms posture or angles (°), and relative time spent at neutral and extreme states (%) (2) Angular movement speed (°/s) and relative time spent at low-speed and high-speed states (%) (3) Relative time spent at 3 states of low speed, neutral, and low speed-neutral of rest/recovery exposure (%)	ActiGraph Software	×/✓
(9) Merino et al. 2018 [63]	3 Workers with musculoskeletal complaints	Cross-sectional case series	(1) Evaluating the risk of musculoskeletal injuries in banana harvesting task using EMG, IMU sensors and subjective outcomes	IMU: upper arms, forearms, and wrists EMG: upper arms	(1) IMU: 6 Xsens MVN Biomech™ sensors (Enschede, the Netherlands, Sampling frequency = 120 Hz) mounted on and attached with Velcro straps (2) EMG: Miotec 4-channel Miotool 400 (sampling frequency = 2 kHz) (3) Ag/AgCl surface electrodes of Meditrace®) in a bipolar configuration (1 cm in diameter and an inter-electrode distance of 2 cm)	(1) Mean joint angles (yaw, pitch, roll; °) and time taken to remove the bunches from the stalk (s) (2) Maximum voluntary isometric contraction (μV), peak muscle use (RMS and percentage) and mean value of signal and median frequency in each muscle (μV and Hz)	Excel/ Xsens MVN Studio Pro/ Miograph	×/✓
(10) Vignais et al. 2017 [76]	5 Healthy workers	Cross-sectional case series	(1) Performing a real-time ergonomic analysis on workers dealing with material handling tasks using IMUs and electro goniometers based on combining RULA computation and a subtask video analysis	(1) IMU: upper arms and forearms (2) Electro goniometer: wrists	(1) 4 CAPTIV Motion IMUs (TEA, Nancy, France) fixed with adjustable strap (sampling frequency = 64 Hz) (2) 2 bi-axial electro goniometers (Biometrics Ltd.,UK) attached by medical tape and straps (sampling frequency = 32 Hz)	(1) RULA score for each joint and an overall score (1 to 7), and percentage of time spent of each joint at risky levels (%) [Using 3 axes shoulder, elbow, and wrist joint angles (yaw, pitch, roll; °)]	CAPTIV software	×/✓
(11) Vignais et al. 2013 [79]	12 Healthy workers	Randomized clinical trial	(1) Performing a real-time ergonomic analysis of manual tasks in an industrial environment dealing with manual handling tasks using IMUs and electro goniometers based on RULA computation for body overall and local parts (2) Assessment of an auditory feedback to prevent development of MSK disorders in workers	Similar to previous study	(1) 4 wireless Colibri IMUs (Trivisio GmbH, Trier, Germany, sampling frequency = 100 Hz) wrapped around limbs with adhesive bands (2) Goniometer type is similar to previous study	(1) RULA score for each joint and an overall score (1 to 7), and percentage of time spent of each joint at risky levels (%) [Using 3 axes shoulder, elbow, and wrist joint angles (yaw, pitch, roll; °)]	No information	✓/✓
(12) Zhang et al. 2022 [61]	30 Manufacturing workers (2 cyclic/ non-cyclic groups of 15 workers)	Cross-sectional case control	(1) Quantification of self-reported daily discomfort, distraction and burden caused by putting on wearable inertial sensors in manufacturing workers (2) Evaluating contribution level of different personal and work characteristics on the discomfort, distraction, and burden ratings	Upper arms, and dominant wrist	(1) 3 IMU sensors of ActiGraph GT9X Links (ActiGraph, USA) fixated with elastic hook and loop fastener straps	(1) Upper arm elevation angle (°), and the magnitude of the elevation speeds (°/s)	ActiLife	✓/×
(13) Seidel et al. 2021 [70]	500 Workers	Cross-sectional case series	(1) Providing direct measurements for assessment of workloads of the hand or elbow in work-field based on the Threshold Limit Value (TLV) for Hand Activity Level (HAL) (2) Finding associations between measured TLV for HAL and disorders or complaints	IMU: shoulders, forearms, elbows, and wrists EMG: Forearms	(1) CUELA multi-sensor system (IFA, Germany) containing potentiometers and IMUs (Analog Devices ADXL 103/203 3D accelerometers and muRata ENC-03R gyroscopes and goniometers), all embedded in a wearable body-shaped cloth (2) A 4-channel surface EMG module (BioMed, Germany)	(1) Mean power frequency of the power spectra of angular data (Hz) [Using 3 axes angular values of measured joints (yaw, pitch, roll; °)] (2) Mean angular velocity (°/s) (3) Kinematic micro-pauses (%) (4) HAL exposure categories (Low/Medium/High) [Using RMS values of EMG signal (V)]	CUELA designed software	×/×
(14) Poitras et al. 2020 [69]	16 Healthy subjects	Cross-sectional case series	(1) Assessment of the concurrent validity of IMU units of MVN, Xsens in comparison to VICON motion capture system during simple tasks and complex lifting tasks (2) Establishment of the discriminative validity of a wireless EMG system for the evaluation of muscle activity	IMU and markers: shoulders (scapula humerus), and wrists EMG: shoulders	(1) 6 IMU sensors of MTw (MVN, Xsens Technologies, Enschede, Netherlands) fixed with hook and loop straps around arms (2) 9 Vicon MX cameras (Vicon Motion Systems Limited, Oxford, UK) (3) EMG sensors of Trigno Wireless EMG system (Delsys, Boston, MA, USA)	(1) Shoulder ROM and RMSE value with respect to VICON measurement (°) (2) RMS EMG (V, and (% of MVC)	Nexus/MVN studio/ MATLAB	×/✓
(15) Bassani et al. 2021 [71]	1 Healthy subject	Preliminary study	(1) Proposing a wearable monitoring system for sEMG and coherent motion data aimed at real-time tracking of workers’ activity for the analysis and prevention of WMSK disorders	IMU: upper arm, forearm, and hand EMG: forearm	(1) 3 MPU-9250 IMUs (Invensense Inc., USA), enabling 9-axes inertial sensing in addition to a thermal sensor to ease the compensation of the gyro values all embedded in a package and wrapped around the limbs with a stretchable strap (sampling frequency = 100 Hz) (2) 8 sEMG electrodes and acquisition systems (proposed system and g.®USBAmp of Guger Technologies) (3) STM32F407VG ARM Cortex-M4 CPU (STMicroelectronics)	(1) Signal to noise ratio of EMG signals (SNR) [Using RMS EMG signal values (mV)] (2) Acceleration, angular velocity, and angle of joints in all 3 axes (yaw, pitch, roll; g, °/s, and °)	MATLAB	×/✓
(16) Lee et al. 2019 [81]	3 Healthy subjects	Cross-sectional case series	(1) Assessment of the reliability and validity of a novel posture matching method in construction activities using IMU measurements of joint angles	Dominant upper arm wrist, and hand	(1) 3 IMU sensors (I2M IMU system; NexGen Ergonomics Inc., Canada) fixed with stretchable bands and straps through a designed platform	(1) Upper arm joint angles and RMSE values in all 3 axes (yaw, pitch, roll; °)	TK Motion Manager software/ HM Analyzer	×/×
(17) Peppoloni et al. 2016 [72]	10 Healthy supermarket cashiers	Cross-sectional case series	(1) Proposing a novel wearable wireless system capable of assessing the muscular efforts using sEMG and postures of the human upper limb using IMU sensor for WMSK disorders diagnosis	IMU: upper arm, forearm, and hand, EMG: forearm	(1) 3 IMU sensors embedded inside elastic band (no further information) (2) 8-Channel sEMG (no further information)	(1) Shoulder, elbow and wrist extension/flexion, and ulnar deviation (yaw, pitch, roll; °) (2) RMS values of EMG signal (mV) and power spectral density (W/Hz) (3) Strain inde and RULA score (1 to 7)	Self-developed GUI by MATLAB	×/✓
(18) Battini et al. 2014 [82]	No information	Preliminary study	(1) Introducing an innovative full-body real-time ergonomics assessment system for manual material handling in warehouse environments, using inertial sensors	Shoulders (scapula and humerus), forearms, and hands	(1) IGS-180i (Animazoo, UK) containing 8 IMU sensors for upper body section and mounted on a light full-body suit (sampling frequency = 500 Hz)	(1) Ergonomic evaluation scores such as RULA, and Lifting Index [Using shoulders (scapula), upper arms, forearms, and hands joint angles and postures with 6-DoF (yaw, pitch, roll; °)	Self-developed software	×/✓
(19) Slade et al. 2021 [83]	5 Healthy subjects	Cross-sectional case series	(1) Presenting the OpenSenseRT, an open-source and wearable system providing upper and lower extremity kinematics in real time using IMUs (2) Assessment the accuracy of the proposed system by comparing it to an optical motion capture as the gold standard seeking to achieve an RMSE of 5° or less for 3D joint angles	IMU and markers: Upper arms, forearms, and hands	(1) 6 IMU (ISM330DHCX breakout boards, Adafruit Industries Inc., USA) mounted on stretchable straps (2) A Raspberry Pi 4b + (Raspberry Pi Foundation) as the microcontroller (3) Optical motion capture system (Optitrack)	(1) Shoulder joint angles and RMSE during flexion, adduction, and rotation (yaw, pitch, roll; °) (2) Elbow joint angle and RMSE during flexion (pitch, °) (3) Wrist joint angle and RMSE during flexion (pitch, °)	Self-developed software by OpenSense tools	×/×
(20) Yang et al. 2020 [58]	116 Surgery cases ( by 53 surgeons)	Cross-sectional case series	(1) Identifying risk factors and assessment of intraoperative physical stressors by subjective (questionnaires) and objective outcomes (IMU measurements)	Upper arms	(1) 2 IMU (APDM Inc, Portland, USA) [model has not been specified] (Sampling frequency = 128 Hz)	(1) Mean deviation angle of upper arms (°) (2) Percentage of spent time in the demanding posture during the surgical time (%)	MATLAB	×/×
(21) Hallbeck et al. 2020 [59]	4 Healthy breast surgeons	Cross-sectional case series	(1) Comparing the ergonomics for the beast surgeons between skin-sparing mastectomy (SSM) and nipple-sparing mastectomy (NSM) using subjective and objective measures	Upper arms	(1) 2 IMU (APDM Inc, Portland, USA) [model has not been specified] (Sampling frequency = 128 Hz)	(Secondary) (1) Orientation (angles) of upper arms using mean angle (yaw, pitch, roll; °) (2) Spent time in each RULA level for postures during surgery (%)	MATLAB	×/×
(22) Nath et al. 2018 [84]	2 Healthy workers	Cross-sectional case series	(1) Proposing a new methodology for evaluation of the ergonomic risk levels in warehouse operations caused by overexertion using body motion data (2) Investigating the appropriate data acquisition and processing settings through a leave-one-subject-out cross-validation framework	Upper arm	(1) 1 Smartphone (Google Nexus 5X or Google Nexus 6) strapped around the limb	(1) Acceleration (g), linear acceleration), and angular velocity in all 3 axes (yaw, pitch, roll; g, m/s^2^, and °/s) (2) Activity duration (s) and frequency (%)	MATLAB	×/✓
(23) Jahanbanifar and Akhavian-2018 [77]	1 Healthy construction worker	Preliminary study	(1) Developing a framework for quantification of human force as a risk indicator associated with WMSK disorders in construction workers using wearable sensors	Upper arm	(1) 1 Smartphone (sampling frequency = 35 Hz) fixated with a sports armband (2) SIMULATOR II; Functional Upper Extremity Rehabilitation device (BTEtechnologies inc., USA)	(1) F as the net force exerted (N or kg.m/s^2^) (2) P as the power (W or kg.m^2^/s^3^) (3) d as the displacement of the subject’s arm (m) [Using acceleration, angular velocity and posture values in all 3 axes (yaw, pitch, roll; g, °/s, and °)] (4) t as the duration of the experiments (s)	Sensor Log smartphone application/ Self-developed Python software	×/✓
(24) Cerqueira et al. 2020 [74]	5 Healthy subjects	Cross-sectional case series	(1) The design and development of an innovative smart garment providing (a) real-time ergonomic risk assessment, (b) objective data measurements to ergonomists, (c) posture awareness to operators through haptic feedback	Upper arms	(1) 2 IMUs; 9250 (Invensense Inc., USA) attached to a skinny fitted shirt (sampling frequency = 100 Hz) (2) 2 haptic motors; ERM (Precision MicrodrivesTM, London) operating at 80 Hz and 250 Hz (3) the STM32F4 ARM microcontroller	(1) Upper arm posture, and RMSE in pitch and roll axes (°) (2) Time spent percentage during each risk state or posture state (%)	MATLAB	×/✓
(25) Singh et al. 2017 [85]	4 Healthy surgeons (gynecologists)	Randomized crossover study	(1) Comparing the effect of different chairs on WMSD for surgeons during vaginal procedures based on subjective and objective outcomes (2) Assessment of the WMSD risk in gynecologists	Upper arms	(1) 2 IMU sensors of OPAL(12M SXT version APDM, Inc, Portland, USA) wrapped around limbs with adhesive bands	(Secondary) (1) The percentage of time spent in each RULA score for each body part (%) [Using shoulder elevation angle (°)]	No information	×/✓
(26) Lind et al. 2020 [62]	16 Manufacturing plant workers	Cross-sectional case series	(1) Proposing the developed haptic feedback module of the Smart Workwear System platform (2) Evaluating its user experience and preventive application for reducing biomechanical loads in light repetitive manual tasks	IMU: upper arms Haptic vibrator: 5cm lower than IMUs	The Smart Workwear System haptic feedback module embedded in a workwear shirt containing: (1) 2 IMUs (LPMS-B2 IMU, LP Research, Tokyo, Japan, sampling rate = 25Hz) embedded on stretchy workwear shirt (2) A vibration actuation unit (Precision Microdrives Limited, London, UK, feedback level = 10Hz) (3) STM32 microcontroller	(1) Angles of upper arm elevation (°) (2) Proportion of time-spent at upper-arm elevations ≥ 30◦, ≥ 45◦ and ≥ 60◦ (%)	ErgoRiskLogger smartphone application	✓/✓
(27) Granzow et al. 2017 [64]	14 Reforestation hand planters	Cross-sectional case series	(1) Characterizing the trunk and upper arm postures, movement velocities, and neck/shoulder muscle activation patterns in hand planters during full-shift work (2) Comparing the results with previous findings to understand the exposures to physical risk factors of hand planters	Shoulder	(1) IMUs: 1 ActiGraph GT9X (Actigraph, USA, sampling frequency = 100 Hz) attached with elastic neoprene straps (2) EMG electrodes (model SX230, Biometrics Ltd, Gwent, UK)	(1) Shoulder muscle forces as a percentage of the RMS EMG amplitudes observed for the submaximal reference contractions (%) (2) Upper arm flexion/extension angles (°) and angular velocity (°/s) (3) The ratio of time spent at three postures of neutral, rest and extreme, and at two velocities of low and high (%)	Self-developed GUI by LabView	×/×
(28) Khalil et al. 2021 [65]	34 Baseball pitchers	Cross-sectional case series	(1) Determining the relation between medial elbow torque measured by wearable sensor IMU, and adaptations of the medial elbow structures obtained by dynamic ultrasound imaging in asymptomatic collegiate pitchers	Elbow	(1) 1 Motus Global mobile IMU sensor (Motus Global Inc., USA) embedded in a pocket of a wearable sensor baseball sleeve (Motus Global, Rockville Centre, NY)	(1) Medial elbow torque (N.m) (2) Arm rotation (maximum angle of the forearm; °), (3) Arm slot (angle of the forearm in relation to the ground at ball release; °) (4) Arm speed (maximum rotational velocity of the forearm; rotations/minute)	Motus Global smartphone application (motusTHROW)	×/✓
(29) Villalobos and Maccowlry 2021 [86]	20 Meat cutters	Cross-sectional case series	(1) Presenting an application of IMUs to perform task classification and measure work-related musculoskeletal disorders risks in meat cutters, using artificial intelligence and machine learning techniques (2) Validation of the proposed application	Wrist	(1) A Wit Motion BWT901CL Bluetooth 2.0 IMU sensor (Wit intelligence, China) attached with a Velcro strap	(1) Angle, angular velocity, and acceleration of wrist/hands (yaw, pitch, roll; °, °/s, g) (2) Wrist/hands RULA score (1 to 4) and the spent time in a risky posture based on RULA score (s)	Self-developed GUI	×/✓
(30) Forsman et al. 2021 [78]	6 Healthy subjects	Cross-sectional case series	(1) Developing and testing the validity of a method for workplace wrist velocity measurements	Wrist and hand	(1) 2 IMUs (Movesense.com, Suunto, Vantaa, Finland, sampling frequency = 50 Hz) attached with double-sided tape or an armband	(1) Wrist angular velocity (yaw, pitch, roll; °/s)	Self-developed smartphones app	×/✓
(31) Rodríguez-Vega et al. 2022 [73]	1 Healthy subject	Preliminary study	(1) Development of measurement and classification of hand movements at work, using a hand-motion capture system	Hand and fingers	(1) 6 IMU sensors (2) 6 Force-resistive sensors (No further details)	(1) Triaxial acceleration, angular velocity, and magnetic field (yaw, pitch, roll; m/s2, rad/s, and µT) (2) Exerted force each fingertip (μV)	MATLAB	×/×

ROM: Range of motion; sEMG: Surface electromyography; RULA: Rapid Upper Limb Assessment; EMG: Electromyography; IMU; Inertial Measurement Unit; GPS: Global Positioning System; RMS: Root Measn Square; MVC: Maximum Voluntary Contraction; GUI: Graphical User Interface; Ag/AgCl: Silver/Silver chloride; SNR: Signal to noise ratio; 3D: Three dimensional; ARM: Advanced RISC Machine; AVR: Advanced Virtual RISC; V: Volt; W: Watt; s: Second; ms: Millisecond; °: Degree; m: Meter; cm: Centimeter; °c: Degree Celsius; Hz: Hertz; g: g-force [Unit of acceleration]; WMSD: Work-related musculoskeletal disorder; RMSE: Root mean square error; μV: Microvolt; MVC: Maximum voluntary contraction; kg: Kilogram; RMS: Root mean square; N: Newton; T: Tesla; rad: Radian

**Table 3:** General wearable design/performance studies

Author year	Participants	Study design type	Aim	Sensor placement	Sensor type, model (provider), utilized number, and wearable platform [core processing unit is included in case of reporting by authors]	Measured outcome(s) [secondary outcomes are indicated with ‘secondary’]	Software for processing/data display	Home- based/ comfortability/ wireless? (✓/×)
(1) Hong et al. 2021 [87]	Subjects at risk of developing MSK disorders	Preliminary study	(1) Proposing a kirigami-structured highly anisotropic piezoelectric network composite (HAPNC) sensor for monitoring multiple information of joint motions, and sending an alarm to prevent MSK disorders	Shoulders	Multilayer HAPNC sensor: a cross-shaped polyethylene terephthalate (PET) substrate, with a square top and bottom layer with two electrodes and one piezoelectric composite	(1) Bending angle (°) (2) Bending radius (mm) (3) Voltage recorded from sensors movement (mV)	Self-developed software/ COMSOL Multiphysics	×/×/×
(2) Jang et al. 2020 [99]	28 Healthy subjects	Cross-sectional case series	(1) Proposing a novel method for monitoring bad postures, including the forward head posture, rounded shoulder, and elevated shoulder	Upper arms	2 IMU sensors of EBIMU24GV3 (E2BOX. China), embedded in a self-designed wearable fixture tool (made by a 3D printer) that can be wrapped around the shoulder (sampling frequency = 10 Hz)	The shoulder symmetry angl represented by percentage (%) [Using elevation angle of shoulders (°)]	No information	×/×/×
(3) Matiur Rahman et al. 2021 [97]	10 Healthy subjects	Cross-sectional case series	(1) Identifying and discriminating subject-specific EMG signal patterns between five elbow joint angles (0°, 30°, 60°, 90° and 120°) during MVCs of sEMG	Upper arms	(1) A wearable 3 channel-based EMG system of SH-SHIM-KIT-004 (SHIMMER™, Ireland) with 1-kHz sampling rate (2) Wet Ag/AgCl surface electrodes in bipolar setting and diameter of 4 mm	(1) sEMG signal (V)	MATLAB	×/×/×
(4) Zabat et al. 2015 [88]	1 Healthy athlete	Preliminary study	(1) Proposing an embedded system that measures joint angles in 3 axes using gravitational acceleration and magnetic field sensors measurements	Upper arm	(1) 1 LSM303DLHC sensor (STMicroelectronics Inc., Italy) containing a 3-axis digital accelerometer and magnetometer embedded in a prototype box package (2) PIC18f2550 microcontroller	Flexion/extension, abduction/adduction and internal/external rotations of the upper limb joint angles (yaw, pitch, and roll; °)	Self-developed GUI with Microsoft Visual C# (.NET Framework)	×/×/×
(5) Romero avilla et al. 2020 [96]	10 Healthy subjects	Cross-sectional case series	(1) Demonstrating the proof-of-principle of an innovative sEMG sensor system, independently used by patients for detecting their muscular activation	Upper arm and forearm (arranged circularly)	(1) 8 EMG modules each containing two bipolar sEMG leads equipped with seven dry cap-electrodes; embedded in a rotation-symmetrical flexible armband (2) ARM microcontroller (EFM32WG 380 F 256, Silicon Labs)	(1) RMS values EMG signal (mV)	No information	✓/×/×
(6) Jurioli et al. 2020 [98]	16 Healthy subjects	Cross-sectional case series	(1) Introducing a low-cost wearable device integrated to VR environments aiming to provide a better-quality rehabilitation process for most patients with motor disabilities	Forearm	(1) 1 IMU sensor of MPU-6050 (InvenSense Inc, USA) (2) Arduino Nano microcontroller (3) All instruments encapsulated in a 3D printed box made using ABS (Acrylonitrile Butadiene Styrene) plastic and wrapped around the forearm with Velcro tapes	(1) Wrist positions in relation to the elbow (yaw, and roll, °) (2) Time spent for completing the puzzle game (s)	Self-developed smartphone application	×/✓/✓
(7) Elshafei and Sheihab 2021 [89]	20 Healthy gym-goers	Cross-sectional case series	(1) Adopting a wearable approach for detecting biceps muscle fatigue during a bicep concentration curl exercise using an IMU wearable sensor	Wrist	1 IMU (Apple Watch Series 4, Apple Inc., USA) with sampling frequency of 50 Hz	(1) X-axis and Z-axis angular velocity and posture (°/s, °), Y-axis of accelerometer (g) (2) Total exerted force of hand (N) [using product of used dumbbells mass and acceleration]	No information	×/×/✓
(8) Karunarathne and Pathirana 2014 [100]	4 Healthy subjects	Cross-sectional case series	(1) Investigating the applicability of some typical solutions of Wahba’s Problem and ordinary filtering mechanism with IMU sensor measurements for obtaining precise kinematics of human	Elbow and wrist of left arm	2 BioKin IMU sensors (BioKin Inc., Australia)	(1) The Root Mean Square Error (RMSE) of measured angles in both methods of IMUs and VICON motion capture (°) [Using wrist and elbow joint angles, accelerations, and velocities (yaw, pitch, roll; °, °/s, g)]	No information	×/×/✓
(9) Young et al. 2021 [101]	10 Healthy subjects	Cross-sectional case series	(1) Quantifying the accuracy and evaluating the validity of a novel proposed design to assess wrist ROM with respect to optical motion capture system as a gold standard through calculating their correlation coefficient	Wrist	(1) The set (3D printed in polylactic acid); contains 2 rotary position sensor of Bourns Model 3382 (Bourns, Riverside, CA, USA) embedded in the wrist strap (2) Teensy 3.5 microcontroller (PJRC, Sherwood, OR) (3) VICON motion capture system using 5 T160 and 4 Bonita cameras (Vicon Motion Systems Ltd., UK)	(1) Wrist flexion/extension and radial/ulnar deviation angles (pitch, yaw; °)	Python	×/×/×
(10) Hochman et al. 2020 [94]	7 Healthy subjects	Cross-sectional case series	(1) Proposing a novel method of measuring the joint acoustic emission of wrist using acoustic emission sensing method	Accelerometers: wrist IMU: hand	(1) 4 Uniaxial accelerometers of 3225F7 (Dytran Instruments Inc., USA) used as contact microphones (2) USB-4432 data acquisition (National Instruments, USA) recording vibrations at a sampling rate of 50 kHz (3)1 BNO-055 IMU package (Ardafruit Industries Inc., USA; Sampling frequency = 100 Hz) embedded in a silicone gel grip	(1) The signal-to-noise ratio (db) of recorded signal of microphones (uniaxial-accelerometer) (2) Wrist angle and acceleration in 3 axes (yaw, pitch, roll; °)	MATLAB	×/×/×
(11) Saito et al. 2017 [90]	No information	Preliminary study	(1) Introducing a novel strain sensor using pyrolytic graphite sheet (PGS), a low-cost, simple, and flexible material, for the application of wearable devices for monitoring human activity	Elbow and middle finger	(1) Small and thin films are cut from 17-µm-thick PGSs, and then attached to a flexible plastic substrate (2) Silver conducting paste as electrodes are wired on the films for electrical measurements, and the adhesives cover the silver electrodes for mechanical failure prevention	(1) Flexion/extension motion of elbow and fingers through mapping the resistance of the strain sensor (Ω)	No inforamtion	×/×/×
(12) Xie et al. 2020 [95]	No information	Preliminary study	(1) Proposing a novel method of monitoring biomechanical motion of joints (or eye blinking) based on electromagnetic sensing techniques	Index finger and wrist	(1) A self-developed high-speed Electromagnetic testing instrument based on Field Programmable Gate Array (FPGA), performing digital demodulation at 100 k/second and features an Ethernet communication (2) Electromagnetic coils (the excitation coil driven by an alternating current ∼48 mA rms)	(1) Finger and wrist bending level and frequency through EM impedance (Ω)	No information	×/×/×
(13) Smondrk et al. 2021 [91]	1 Healthy subject	Preliminary study	(1) Designing and realizing a device for measurement of finger flexion, and extension and forearm motion (2) Assessment of the device reliability	Bend sensor: fingers IMU: forearm	(1) 5 Flexible bend sensors (Flexpoint Sensor Systems, USA) embedded inside the instrumented glove (2) A 9-axis IMU, LSM9DSO (ST Microelectronics, Switzerland) (3) A microcontroller unit of ATmega328P (Microchip Technology, USA)	(1) Fingers joint angles (pitch, °)	No information	×/×/✓
(14) Zheng et al. 2016 [92]	5 Healthy subjects	Cross-sectional case series	(1) Developing a sensor glove (called FuncAssess) and evaluation of its validity and reliability (2) Proposing a method for visualizing and quantifying the abnormality of the inter-joint coordination	Fingers	(1) Glove fabric made from polyamide stretchable fabric including sleeves for insertion of force sensors (2) 10 Bend sensors of Flexpoint Sensor Systems (Draper, USA, sampling frequency = 50 Hz) (3) 5 Force sensors of Flexiforce (Tekscan, Inc., USA) (4) MSP430 microcontroller (Texas Instruments, Inc., Dallas, TX)	(1) Finger joints bending angle (°) (2) Finger joints load (gram)	MATLAB	×/×/×
(15) Moreira et al. 2014 [102]	1 Healthy subject	Preliminary study	(1) Proposing a glove aiming to (a) track hand and fingers, while minimizing drift and offset errors (b) avoid the need for cumbersome calibration procedures and (c) evaluate its reliability and validation	Hand and fingers	(1) Glove fabric is made from polyamide stretchable fabric (2) 11 9-DOF IMUs including a 3-axis gyroscope sensor of L3GD20, STMicroelectronics Inc., Italy) and 3-axis accelerometer and magnetometer sensor of LSM303DLHC (STMicroelectronics Inc., Italy), all stitched to the fabric with four fixation points (2) STM32F4 ARM processor	(1) Fingers and hand joint angles (yaw, pitch, and roll; °)	Python	×/×/✓
(16) Hazman et al. 2020 [103]	10 Healthy subjects	Cross-sectional case series	(1) Developing a glove for finger joint measurement for collecting ROM values of distal interphalangeal (DIP), proximal interphalangeal (PIP) and metacarpophalangeal (MCP) joints of an index finger	Index finger	The proposed glove: (1) Made from cloth material (1) 3 6-axis IMUs, (2) 2 2.2-inch bend sensors, (3) Arduino Nano (AVR microcontroller- ATMega328)	(1) ROM of the MCP, DIP, PIP joints in angles (yaw, pitch, and roll; °) (2) Percentage of error between both methods (%)	A self-developed GUI on MATLAB	×/×/×
(17) Oigawa et al. 2021 [93]	2 Healthy subjects	Cross-sectional case series	(1) Investigating a novel method for evaluating hand movement function through fingertip data during a 10-s grip and release acquired by wearable contact-force and accelerometer sensors	Fingers	(1) 2 Contact-force sensors of HapLog (Kato Tech Co., Ltd., Kyoto, Japan) each containing (a) a triaxial accelerometer and a strain sensor mounted on the cover sensor (b) a bangle-type connector (c) a calibration unit	(1) 3 Axes, and absolute acceleration (yaw, pitch, roll; g) (2) Contact force (N)	HapLog software	×/×/×/
(18) Rovini et al. 2020 [104]	20 Healthy subjects	Cross-sectional case series	(1) Proposing an innovative, ring-shaped wearable system, called SensRing, that provides inertial data of fingers during the movement (2) Performing a preliminary technical validation to compare the measured data of the SensRing to a motion capture system of Vicon (as the gold standard) on two finger tapping exercises	Fingers	(1) SensRing: a ring-shaped wearable sensor, including (a) a LSM9DS1 IMU sensor (STMicroelectronics, Italy, sampling frequency = 50 Hz) (b) STM32-F103 ARM microcontroller (STMicroelectronics, Italy) [The processing board and device are fixed to the wrist with an elastic band] (2) The Vicon system including 8 cameras (sampling frequency = 100Hz)	(1) Triaxial accelerations and angular velocities (yaw, pitch, roll; g, °/s) (2) Number of repetitions, frequency of the movement (Hz), and range of index finger movement (°)	No information	×/×/✓

ROM: Range of motion; sEMG: Surface electromyography; EMG: Electromyography; IMU; Inertial Measurement Unit; GPS: Global Positioning System; RMS: Root Measn Square; MVC: Maximum Voluntary Contraction; GUI: Graphical User Interface; SNR: Signal to noise ratio; 3D: Three dimensional; ARM: Advanced RISC Machine; AVR: Advanced Virtual RISC; V: Volt; s: Second; ms: Millisecond; °: Degree; °c: Degree Celsius; Hz: Hertz; g: g-force [Unit of acceleration]; RMSE: Root mean square error; RMS: Root mean square; N: Newton; T: Tesla; db: Decibel; Ω: Ohm

**Fig. 2:**
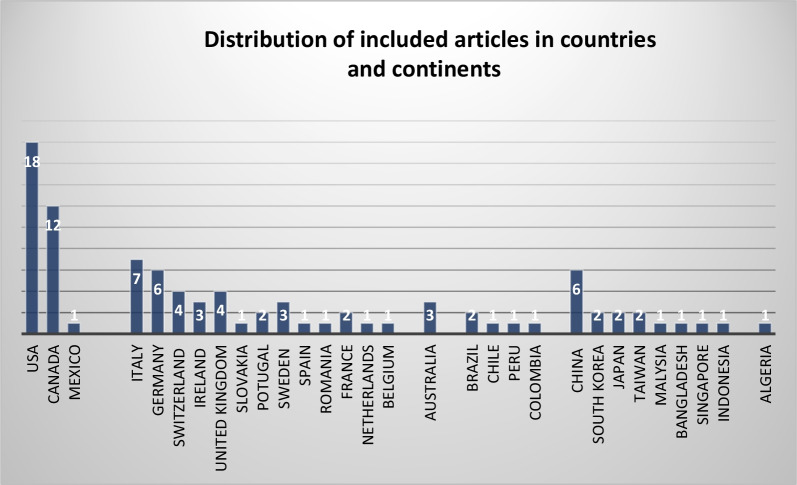
Number of included studies sorted by the countries

Among the identified body of evidence, various researchers from 30 countries have contributed to this field by conducting studies. In Fig. 2, the number of conducted studies in each country has been demonstrated in a bar chart. Researchers of the USA, Canada, Italy, China, and Germany are pioneers in this field by presenting 18, 12, 7, 6, and 6 studies, respectively. It must be noted that in the case of collaborations of researchers from different countries, all author’s affiliations have been considered the origin country of research.

**Fig. 3:**
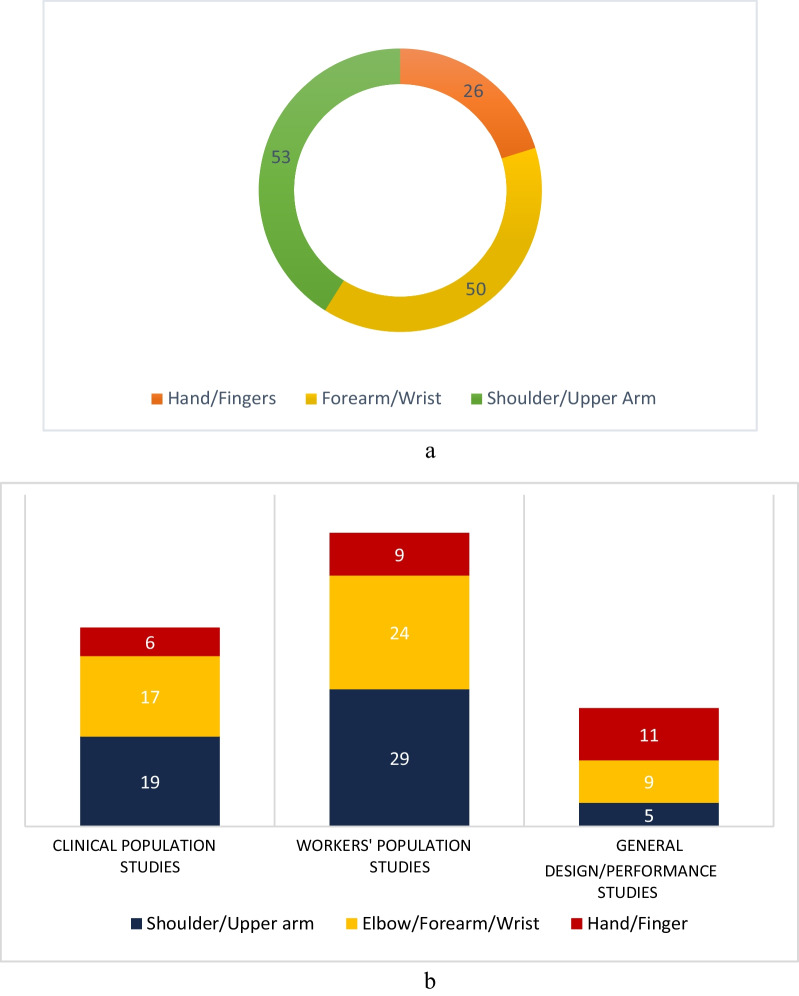
**a** Sensor placements at different segments for all included studies; **b** Sensor placements at different segments in clinical population, workers’ population, and general design/performance studies, separately

The sensor placement locations were sorted into (1) shoulder and upper arm (scapula and humerus), (2) elbow, forearm (ulna and radius), and wrist and (3) hand, and finger (phalanges). (Fig. 3) Most of the included studies (n = 76) have attempted to measure angles of these limbs (for obtaining Range of Motion or ROM) alongside their angular velocity or acceleration through exploiting IMU, piezoresistive, or optical sensors providing three-dimensional values (represented in Quaternion axes, Eulerian axes, or yaw/pitch/roll). In these studies, subjects performed a series of various joint movements such as flexion/extension, abduction/adduction, and internal/external rotation or a combination of these motions to obtain the measurement parameters of that joint. A summary of the data collection procedures along with the critical points regarding the processing methods used for the translation of obtained joint data into meaningful clinical or non-clinical outcomes have been reported for all three categories in Additional file 2: Appendix B.

**Fig. 4:**
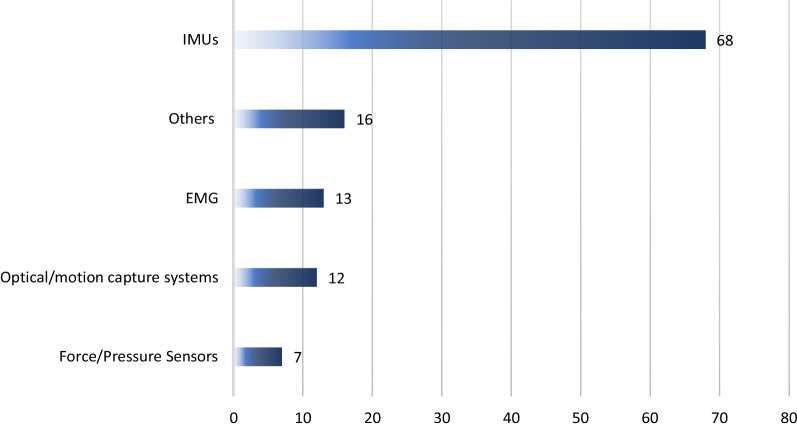
Number of different types of sensors used in all studies; Examples of others include smartwatches, self-developed systems, LED, and non-IMUs

The outcome column of the following tables summarizing the extracted data of included studies presents the corresponding outcome that has been directly or indirectly obtained via wearable sensor platform. If the targeted outcome of a study is indirectly measured by the wearable sensor platform, the sensor-measured parameters (joint angle, acceleration, or angular velocity) are mentioned in brackets. Moreover, if the outcome obtained by wearable sensors is a secondary outcome of the study, it is demonstrated in that section. Since the assessment and collection of the statistical results of the studies are outside the scope of this scoping review, the related information about the statistical processes and analyses has not been reviewed. We also presented an overall visual representation of the total number of various sensor types used in all the studies, utilizing a pie chart (Fig. 4).

### Clinical population studies

In this category, either the participants of the included study were MSK patients, or the introduced wearable platform was designed and aimed to be applied to individuals with MSK conditions. A total of 31 articles were identified to be included in this category. Nineteen studies out of 31 were related to applications of wearable sensors for shoulder or upper arm conditions. However, the sensor placement of some of these studies included other upper body parts such as the wrist [24–28] and hand [29]. One of the included studies has utilized a wrist wearable system for assessment purposes on patients with distal radius fractures [30]. Three other studies on arthritis and motor impairment patients have used wrist-mounted wearable sensors [31–33]. Five studies focused on hand and finger diseases such as hand arthritis [34, 35] and carpal tunnel syndrome [36, 37], applied wearable sensors (such as IMUs, and piezoresistive sensors) that were either embedded in a glove or attached with straps.

**Fig. 5:**
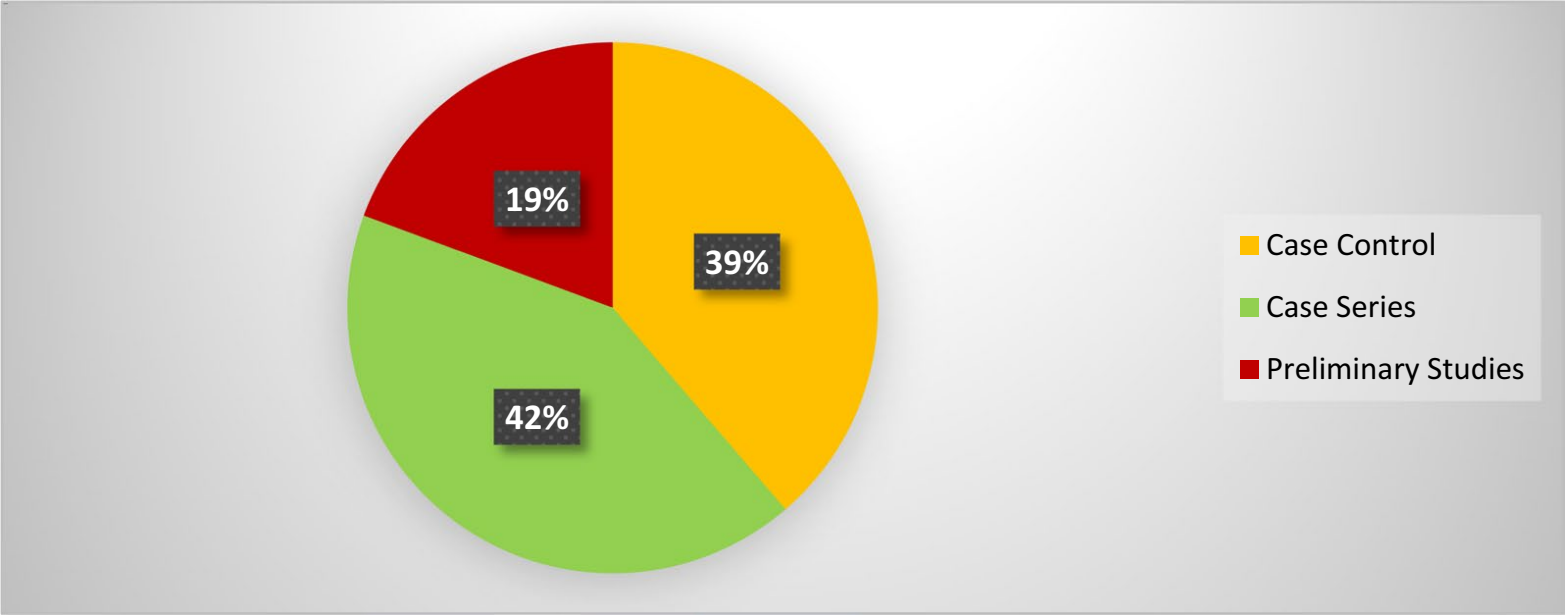
Study design types of included articles in the clinical population studies category

The study design types of included articles in this category were case–control studies (12 studies), case series studies (13 studies), and preliminary studies (6 studies), without any Randomized Clinical Trials (RCT) studies (Fig. 5).

Most of the studies (n = 26, 84% of clinical population studies) exploited IMU sensors in their wearable platform (usually containing a 3-axis accelerometer, 3-axis gyroscope, and 3-axis magnetometer) to obtain joint angles, accelerations, angular velocities, and posture. While only five studies have included EMG sensors in their wearable platform to obtain muscle activity [30, 38–41]. Some of the studies have also used other instruments and sensors to obtain measurements or treat an MSK condition, such as using high-power LEDs [42] and thermal flexible printed circuit boards for treatment [32] or using piezoresistive or strain sensors for measurements [35–37, 43]. In the sensor type and hardware column of Table 1, the model and provider company of sensor, the attachment or embedding method of the sensor in wearable platform, core processing unit, and data sampling frequency of wearable sensors were indicated (in case of reporting by authors).

In the included studies, participants completed functional activities or exercises, like shoulder flexion/extension, abduction/adduction, and internal/external rotation, or wrist, elbow, and fingers flexion/extension for a specific set of repetitions in standardized conditions such as in studies conducted by Duc et al. [38], Kwak et al. [24], and Chen et al. [44]. Some studies have collected the data in a non-standardized set up like a home setting. In these studies, participants’ daily activities have been recorded and analyzed, such as in studies conducted by Pichonnaz et al. [45], Duc et al. [46], Van de Klut et al. [47], Langohr et al. [48], and Haverstock et al. [49]. The explained wearable systems have been identified as home-based wearable systems and have been reported in Table 1. It must be noted that usually, the calibration process of sensors has been described, and a summary of these procedures has been included in Additional file 2: Appendix-B (in case of reporting by authors).

**Fig. 6:**
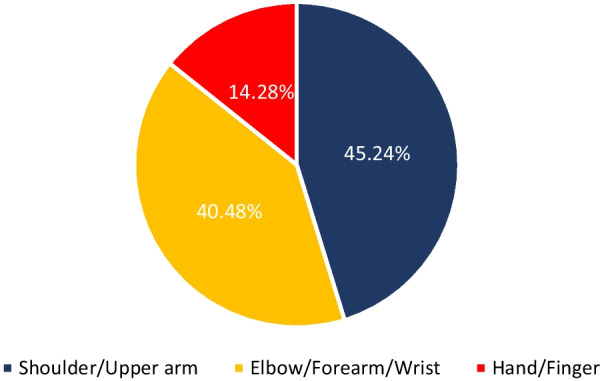
Sensor placements at different upper limb segments for Clinical population studies category

The software utilized for each study’s processing phase (translating raw data into the intended outcomes) has been indicated in the “Software for processing/data display” column of Table 1.Each study’s home-based applicability, comfortability assessment, and wireless data transmission ability have been examined in the final column. In this regard, the introduced wearable system of 17 studies (55% of this category’s studies) has been recognized as home-based applications. Only 1 study (3% of this category’s studies) have assessed the comfortability of their applied wearable sensor platform in subjects [25], and 10 studies (32% of this category’s studies) have explicitly mentioned the ability to send the measured data through a wireless system [25–27, 29, 33, 35, 37, 39, 40]. This ability is a crucial parameter for the future development of wearable systems. The share of each sensor placement location in the studies has been demonstrated (Fig. 6).

### Workers’ population studies

**Fig. 7:**
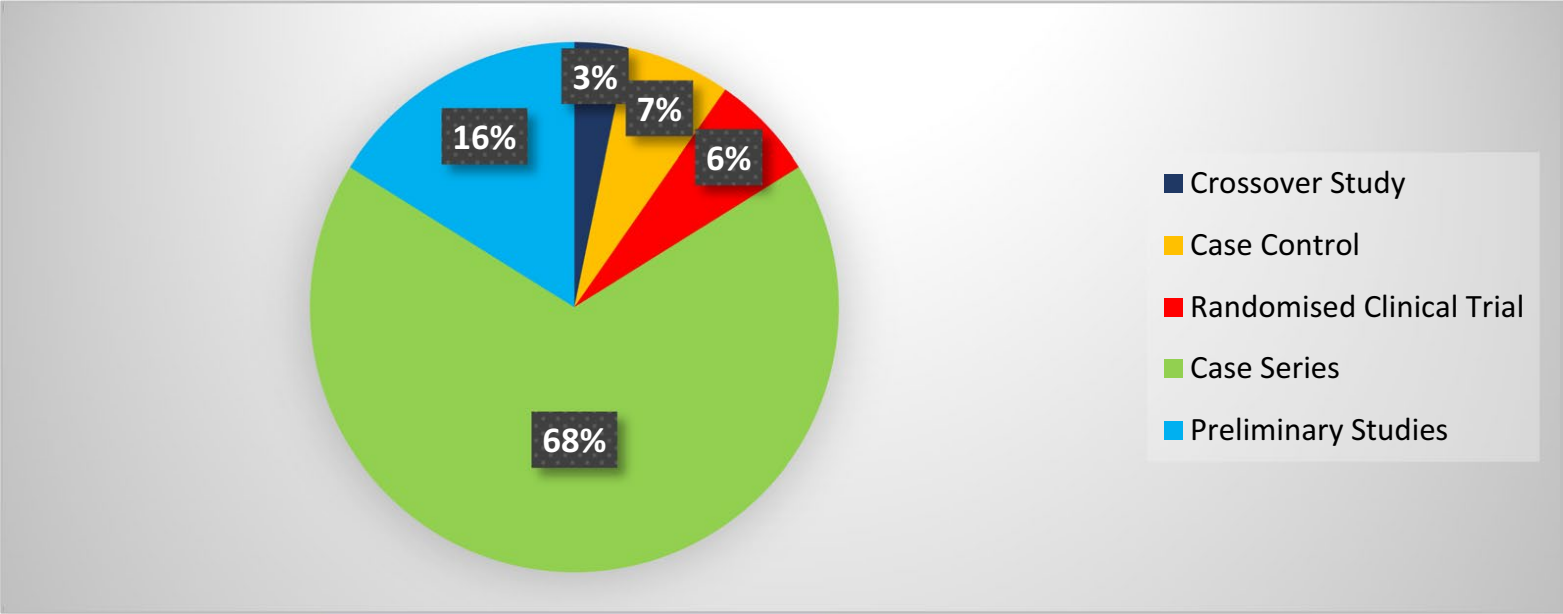
Study design types of included articles in the Workers’ population studies category

In this category, 31 studies have focused on the workers’ population and the risk of work-related musculoskeletal (WMSK) conditions or disorders. Two RCT studies, a crossover study, two case–control studies, 21 case series studies, and 5 preliminary studies are included in this category (Fig. 7).

**Fig. 8:**
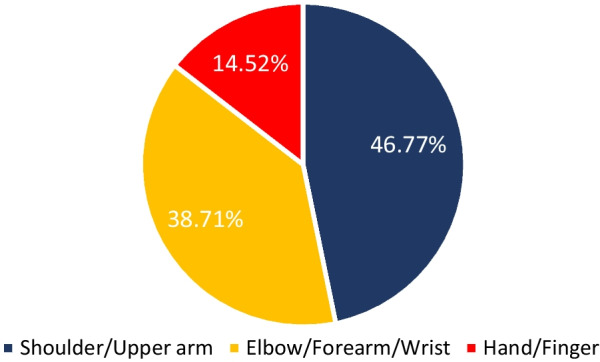
Sensor placements at different upper limb segments for the Workers’ population studies category

From the perspective of sensor placement location, 21 Studies (68% of studies in workers’ population category) have placed the sensors on multiple arm segments (the most common segments are the upper arm and shoulder). This can indicate that the most studies of this category have correctly assessed more than one single-point arm segment to obtain a complete perspective of the risk involved in various working conditions. It can be deduced that the primary focus point of these studies is upper arm segment (Fig. 8).

The participants of this category’s studies form diverse samples of different populations including, dentists [55–57], surgeons [58, 59], warehouse and manufacturing workers [60–62], farmers [63, 64], athletes [65], and other areas. According to Table 2, nearly all the studies have either aimed to investigate ergonomic risk levels of a specific job or measure the exposure to ergonomic risks related to WMSK conditions. A significant number of the studies (about 48% of the included studies) have exploited Rapid Upper Limb Assessment (RULA) score (or a modified version of it), which represents the level of MSD risk for a job task being evaluated [66]. Thus, the RULA score is one of the frequently reported outcomes of a considerable number of included studies of this category. Some of the studies have aimed to evaluate the validity of a proposed wearable sensor platform for monitoring the ergonomic performance of workers with respect to an optical motion capture system accepted as a gold standard [67–69].

Regarding the utilized instruments and hardware of wearable sensors applied in the studies, IMU sensors have been exploited in all studies. In this regard, seven studies have declared that Xsens company (headquartered in the Netherlands) products, including MVN link and Biomech™, were used to obtain inertial measurements [55–57, 63, 67–69]. It can be implicated that due to convenient application, and various provided features, Xsens products are more favorable for researchers aiming to study work-related musculoskeletal risks or non-laboratory setup and locations. Six studies have also utilized sEMG recording electrodes in their system to obtain muscle activity of arm segments [63, 64, 69–72]. One study has included force sensors in addition to embedded IMU sensors in a glove to acquire complementary information regarding the work-related ergonomic risks of hands and fingers [73].

Two studies have devised a haptic feedback system via a vibration actuation unit in their wearable system design to provide alerts for workers for changing their posture [62, 74]. In these studies, vibrations are applied to the subject’s body, warning the wearer after exceeding a certain threshold of angles and spending time durations in high-risk postures. The applied sensors number, type, model, provider of utilized sensors in included studies are presented in the "Sensor type and number" column of Table 2. The attachment or embedding method of the sensor in the designed wearable platform, core processing unit, the data sampling frequency of wearable sensors, and related data of utilized motion capture systems such as VICON are also described in case of reporting by authors.

Regarding data collection methods or intervention procedures, some of the studies’ participants have performed specific movements in a standardized condition, such as in studies conducted by Humadi et al. [67, 68], Lee et al. [75], and Vignais et al. [76]. Some studies have collected the data in a non-standardized setup like a factory or work setting. In these studies, particular durations of work shifts have been recorded and analyzed, such as in studies conducted by Ohlendorf et al. et al. [55], Blume et al. [56], and Schall et al. [60]. A summary of these procedures’ calibration processes has also been reported in Additional file 2: Appendix-B (in case of reporting by authors).

Regarding the software component of studies, nine articles have stated using MATLAB software for processing or displaying their data and results. Other studies have either not declared the specific used processing software (or platform for developing their software) or have utilized the specialized software presented by provider companies of the sensors. Four studies have mentioned using smartphone apps (self-developed or company-developed) as their data processing software or their provided GUI for subjects [62, 65, 77, 78].

In contrast to the previous section, the home-based applicability is not assessed since the studies are focused on workers’ population. Each study’s comfortability assessment and wireless data transmission ability have been investigated and reported in Table 2. In this regard, 22 studies have stated that their wearable platform has the wireless data transmission ability (71% of included studies in this category), which is a significant finding for the studies of this category. Only three studies have assessed the comfortability of their presented wearable sensor platform through questionnaires and interviews [61, 62, 79].

### General wearable design/performance studies

**Fig. 9:**
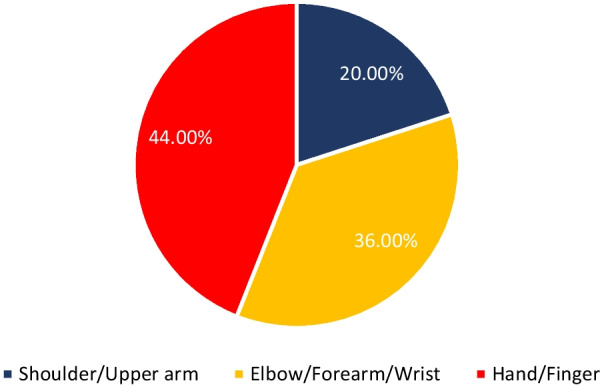
Sensor placements at different segments for the general wearable design/performance studies category

This section explores and summarizes a total number of 18 studies that developed a wearable platform or proposed a novel method or design to assess upper limb movement parameters or muscle activity in individuals without emphasizing a specific context or population (Fig. 9). The wrist, forearm, hand, and fingers are the most common sensor placement locations (n = 8) in this category (Table 3).

All included studies propose novel designs, platforms, measurement, and processing methods. No patient population or participants from specific contexts are included in these studies. Therefore, the study design types of all included articles are either case-series (n = 12) or preliminary studies (n = 6). The participants of this category’s studies are all healthy individuals (general population). However, in one of the studies, the proposed design is suggested for people who might develop MSK conditions [87], and two studies focused on athlete populations [88, 89].

Another significant point in the included studies of this category is the larger proportion of articles utilizing non-IMU sensors (n = 10.56% of studies in general design category). For instance, piezoresistive, bend sensors, and contact force sensors operating based on sensors’ electrical resistance or conductivity change are more exploited to detect joint angles and motion in the general wearable designs [87, 90–93]. A novel approach has been considered by using a different type of accelerometer operating based on contact microphones [94]. In this way, the proposed system can detect and obtain accelerations of sensitive motions. Another novel approach has exploited electromagnetic sensing abilities and electromagnetic coils to detect biomechanical motion of joints through electromagnetic impedance [95]. Two studies have utilized sEMG recording electrodes in their general wearable system designs [96, 97]. The related details of wearable sensors, including the utilized number, type, model, and provider of sensors, are included in the “Sensor type and number” column of Table 3. The attachment or embedding method of sensors in the designed wearable platform or the provided wearable platform (like a fabric glove or sleeve), core processing unit and data sampling frequencies of wearable sensors are also mentioned in the case of reporting by authors.

Regarding the software component of studies, the articles have used MATLAB, Python, Microsoft Visual C# software for processing purposes or designing graphical user interfaces. Only one team of researchers has designed a smartphone app to interact with the proposed wearable system [98].

The number of studies indicating home-based functionality or assessment of comfortability of their proposed designs is significantly lower than the two previous sections. Only one study has claimed that their platform has a home-based functional ability [96], and only one study has focused on assessing their system’s comfortability [98]. This can be related to the fact that most of these studies have only considered the general assessment aspect of their platform (n = 16), and these features are of more significant concern in more focused research and areas. Five studies reported their introduced wearable system’s wireless data transmission ability (28% of studies in general design category). The details of each study have been mentioned and illustrated in Table 3.

In the following, some of the significant findings of the result section are summarized and presented. One of the critical aspects is finding the rate of studies aimed to use wearable sensor platforms for either rehabilitation or treatment applications or increasing the quality of remote treatment sessions. In this regard, 11 studies in the clinical population studies category (35% of studies in the category) [26, 27, 29, 32, 33, 36, 37, 40, 42–44], three studies in the workers’ population studies category (10% of studies in the category) [62, 80, 85], and two studies in the general wearable studies category (11% of studies in the category) have focused on treatment applications [87, 98].

Other investigated features in the synthesized studies were the home-based applicability and the presence of any sort of comfortability assessment. Home-based applicability feature has been explored in the first and third categories (Clinical population studies and General wearable design/performance studies); however, the assessment of comfortability has been explored in all three categories. In this regard, 18 studies in total have reported a home-based assessment or treatment (37% of included studies in clinical population and general design categories). The common element in these studies is preparation and utilization of a convenient wearable platform for subjects in the form of a glove [35]; a band, strap, or watch [25–28, 31, 33, 43, 44, 98], a sleeve [29], and a shirt [47–49, 53]. On the other hand, only five studies (7%) have assessed the comfortability of their wearable systems in subjects through methods such as questionnaires [25, 79] and interviews [62, 79].

Another significant finding is the high and low rate of using IMU (n = 68, 85%) and sEMG (n = 13, 16%) sensors in the included studies, respectively. In total, 13 studies have exploited wearable sEMG systems to obtain muscular activity of subjects. Some studies have used both IMU and sEMG sensors to obtain both joint motion and muscle strength information of participants [30, 38–41, 63, 64, 69–72]. Eight studies have placed sEMG electrodes on upper arms and shoulders, four studies have located sensors on forearms, and one study has recorded both upper arm and forearm activities.

The sensor placement locations of each category were presented in previous sections. In Fig. 3, all categories’ sensor placement locations and the number of utilized upper limb segments in wearable sensor systems of studies have been demonstrated to compare each category (Shoulder/Upper arm, Elbow/Forearm/Forearm/Forearm/Wrist, and Hand/Finger). These systems may place the sensors on multiple segments of the upper limb [28, 67, 96]. Thus, all the segments included in each study have been counted separately in corresponding figures (for instance, if the sensors were placed on the forearm and upper arm, both segments were included in the corresponding figures). In total, 129 sensor systems have been utilized in the included studies.

Designing or using a smartphone application for monitoring or displaying the obtained data in several studies was another significant finding of this review. In this aspect, ten studies have either used or developed a smartphone app in all three categories [26, 27, 31, 33, 62, 65, 77, 78, 89, 98]. Considering the remote data collection method in some studies, this ability can facilitate the supervision and connection of researchers, clinicians, and patients.

## Discussion

This scoping review summarized 80 research studies that addressed and found a variety of wearable sensors applications in assessment, prevention, and treatment of upper extremity MSK conditions with the most common goals of joint motion measurement. Reviewed papers were sorted into three categories of clinical population, workers’ population, and general design/performance studies as three primary application fields. The populations of clinical studies were patients with UE-MSK disorders, while the participants of workers’ population studies were manufacturing workers, athletes, surgeons, farmers, and healthy individuals at risk of developing an MSK condition at work. Prevalence rate of MSK or UE-MSK conditions among US workers’ population was 8.23% according to a conducted study in 2018 [105]. Moreover, 1.7 billion people are dealing with MSK conditions at global scale [17]. Thus, the reviewed information of wearable systems in each category can provide a separate overview of different populations categories and address the available knowledge gaps of each. The third category of General wearable design/performance studies proposed novel systems or settings that were not attributed to a specific patient population or context. This category proves to be advantageous for researchers or designers to be informed about available wearable systems design ideas for their future studies regardless of any specific population or setting.

**Fig. 10:**
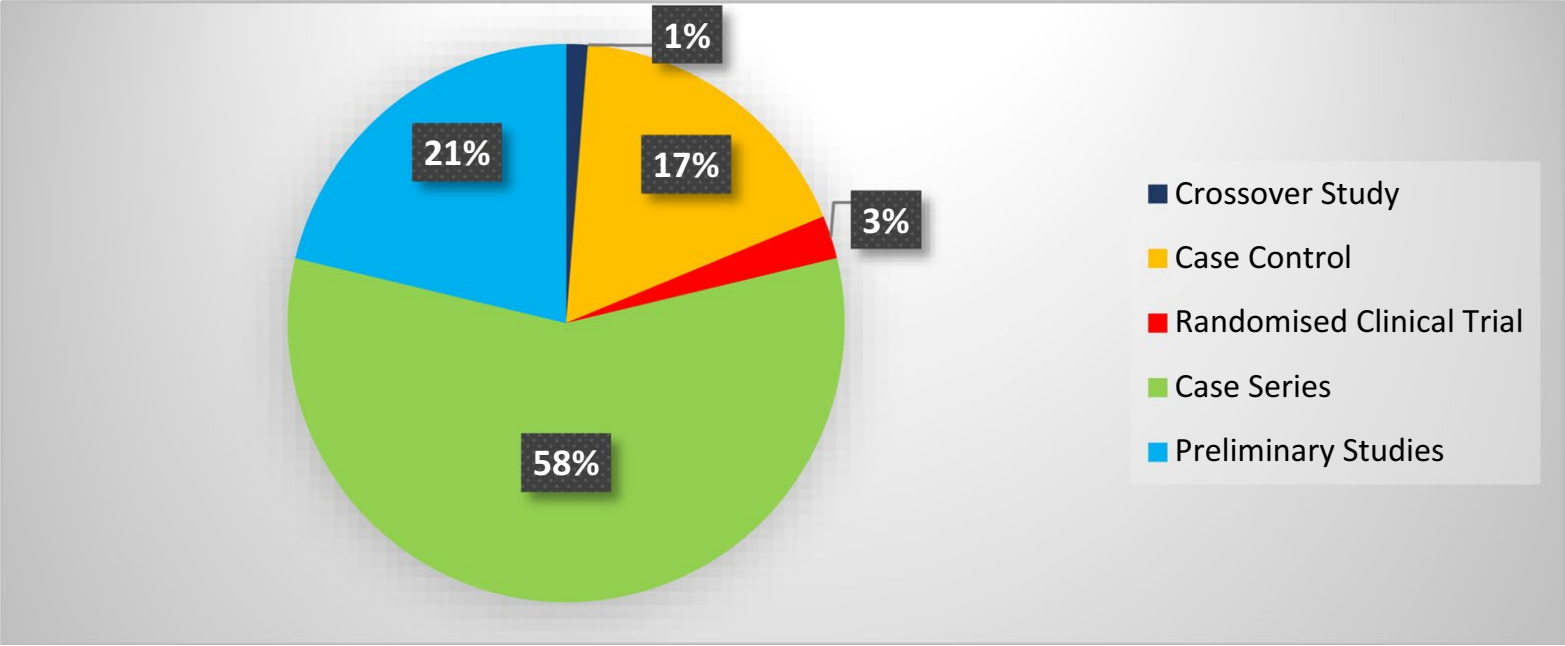
Study design types of included articles in both clinical and workers’ population categories

Clinical population, workers’ population, and general wearable design studies included 25, 24, and 12 observational studies (such as case series, and case control studies), respectively. There were 17 preliminary studies (21%) proposing a novel design, or a processing/classification method were also found and reviewed in all included studies (Fig. 10). A considerable number of included studies in all three categories are conference abstracts that generally represent initial findings on new applications. It may indicate that these applications did not always progress to fully powered studies although it may also reflect the stage of innovation in this field. Two RCT studies were included in the workers’ population category, while no RCT studies were found in the clinical population studies category. Conducting RCTs will obtain more valid results regarding the validity of measured outcomes by wearable systems in the field of UE-MSK disorders. All in all, it appears that studies with more rigorous study designs providing more valid data are still not conducted or scripted. This finding is in accordance with results of [106] and [107], in which the number of clinical trials is either low (eight clinical trials including the use of wearable sensors systems in [106]) or reported to be lower than studies on healthy populations [107]. This gap can be addressed by introducing novel and easy-to-apply wearable systems that can facilitate conduction of various tests on a sufficiently large sample MSK patients and healthy individuals. The collaboration of rehabilitation engineers and clinicians in a study on the applicability of wearable sensors in UE-MSK can also cover both technological and clinical aspects. This association is recommended as a solution to overcome this challenge, as already suggested by Collinger et al. to address similar challenges found in the neural interfaces and integrated prosthetics field [108].

One of the challenging points of included papers is the wearability aspect of utilized sensors and instruments. The included studies have either developed a wearable framework for sensors or have called their system a wearable design. However, according to the perspective of other researchers, "wearable sensors" are defined as electronic systems and computers integrated into comfortable and wearable clothing and other accessories [109]. In a rather distinct definition, implanting sensors in the body can also be considered as developing a wearable system [110]. A considerable number of included studies have merely attached IMU, sEMG, and other sensors to the subjects’ bodies through adhesive tapes and other materials and did not mount, embed, or integrate the sensors into a fabric, textile, or structure. Although they have called and considered their system a wearable design platform, the convenient wearability feature of their system is uncertain. This issue can be attributed to the lack of a widely accepted general definition of wearable sensors. Providing such definitions and standards can appropriately address this issue. In the case of this review, the mentioned studies have not been excluded since the aim of a scoping review is to map all available evidence of the wearable sensors field [111]. Therefore, all these studies have been included to provide a better overview of research contributions.

Another significant point is the low rate of treatment applications in the included studies. In total, only 20% of all studies (n = 16) have considered a treatment application in their reports. These studies have used wearable sensors for unsupervised or indirect treatment. In workers’ population category, 3 studies developed a feedback method that prevents the subjects from developing an MSK condition during working hours. The low rate of treatment applications can be attributed to the fact that clinicians or researchers usually face difficulties in providing patients with wearable sensors that can record and store information for long durations. Another potential cause can be related to the complexity of working with wearable sensors. Investigating the rehabilitation and treatment applications in various conditions and their potential barriers is strongly recommended for future studies. Other studies (n = 64) focusing on assessment applications of wearable sensors provide motion information. Researchers of these studies have aimed to gain valuable data about the treatment process or work risk factors of MSK conditions. The greater number of assessment studies can be related to technological advancements of IMU sensors and big data field [112]. A high rate of wireless data transmission can store and send more than 50 samples of joint orientation and motion per second according to the reviewed papers of this scoping review. Nonetheless, the low rate of using a feedback procedure (visual, auditory, haptic, and other feedback forms) with the available wearable systems can be one of the reasons of low rate of wearable sensors treatment applications in MSK conditions.

Another encountered challenge of this review was finding similar articles about one identical study published by similar research teams. These articles were mainly reported as a single study in this review, and it has been attempted to summarize all essential information of these articles. However, in several cases such as Duc et al./Pichonnaz et al. [38, 45, 46], Burns et al. [26, 27], Humadi et al. [67, 68], and Vignais et al. [76, 79], all found articles have been reported separately since either the interventions and processing methods, wearable instrument, or reported outcomes differed from each other. Therefore, they have been treated as separate studies.

One of the investigated features in the studies was their home-based applicability, and as mentioned in the results section, 37% of studies (n = 18/49) in the clinical population and general wearable design categories assessed or suggested a home-based application of their wearable system. This rate is in accordance with reports of other wearable sensor studies, in which the ability of long motion recording activity or tracking daily activities of participants has been considered challenging [113, 114]. Therefore, enabling home-based motion recording of individuals for long durations deems necessary for future research applications considering that the subjects must be able to set up the settings conveniently and independently at their home for enabling the opportunity of conducting more robust studies. Moreover, the comfortability parameter of the applied systems is another critical aspect that has been investigated in a low share of studies (n = 5, 7%). This feature has a significant impact on the extensive functionality of wearable sensors, especially for recording long daily activity recordings. This rate represents another gap of knowledge in this field. Conducting studies that can provide reliable and valid information on the comfortability of wearable sensors through standard questionnaires and interviews or potential challenges that emerged in using these kinds of systems can guide the developer companies of these systems toward more generalizable wearable systems. Therefore, it is highly recommended that researchers consider the comfortability assessment of their applied wearable sensors in subjects for their future studies.

Most of the papers (n = 68, 85%) have used IMU sensors to obtain inertial data of subjects. However, sEMG recordings were also made in some of the studies (n = 13, 16%). According to these results, ROM and joint angles, or the time spent in certain angle ranges were more considered and measured as the primary outcomes of studies. While muscle strength and maximum voluntary muscle contraction were usually measured as complimentary outcomes. This finding can be primarily attributed to the fact that the sEMG costs, setup process, finding precise sensor placement location, and providing settings for extended recording ability (continuous skin cleaning process) are troublesome procedures [115–117]. While using IMUs do not necessarily require advanced settings or setup [115]. They can operate in wireless setting and without causing notable disturbance in comparison to sEMG sensors. In this scoping review, only two of the studies have proposed a convenient wearable platform for positioning and utilizing sEMG signals [96, 97]. Other studies merely attached the electrodes with straps or adhesive tapes. As mentioned by the authors of this study, this leads to the emergence of challenges in finding accurate and sEMG sensor placement and long extended signal recording durations for future measurements. Thus, providing a comfortable and easy-to-use wearable platform for sEMG electrodes can ameliorate the utilization rate of sEMG systems in future studies.

Regarding sensor placement locations, no significant difference is noted between upper arm/shoulder and forearm/elbow/wrist regions. However, the number of studies that have focused on hand/fingers region is considerably lower than other regions in clinical population and workers’ population studies. It can be speculated that this is due to the convenient placement setting of IMU sensors on upper arm and forearm regions.

Another notable point is the similarity of processing methods of the included studies. The process of obtaining outcome in all three categories of studies usually contain a filtering procedure to clear the noise from obtained data and a classification step to detect a joint movement. Low and high pass filters perform inertial data filtering tasks that result in a bandpass filter. The frequency range of this filter is not unique in the studies; however, the cutoff frequencies are between 0.1 Hz [28] for lower cutoff frequency to 10 Hz [101] for upper cutoff frequency. This finding is consistent with similar reported filter frequencies by Kim et al. [19]. Moreover, the EMG signal filtering is also performed by a bandpass filter. The reported frequencies are between 10 Hz (for lower cutoff frequency) to 500 Hz (for upper cutoff frequency) [30]. On the other hand, the classification step is realized through various techniques. One of the methods to perform classifications is machine learning algorithms like Support Vector Machine (SVM), Convolutional neural network, and k-nearest neighbors, that are applied in studies such as Burns et al. [26], Jang et al. [99], Nath et al. [84], and Rodríguez-Vega et al. [73]. Another classification method is simple thresholding, which is considered by Pichonnaz et al. [45], Duc et al. [38], and Larrivée et al. [25]. All these classification methods have unique advantages and disadvantages that must be utilized according to the study’s aims and applications. Further investigation of these methods is beyond the scope of this paper. However, important points about each study’s applied processing and classification methods have been reported in Additional file 2: Appendix B of this review. As mentioned in previous sections, the statistical analysis of studies has not been reviewed or summarized in this review. Thus, the utilized software for statistical analysis purposes of studies has not been reported. Nonetheless, the related analytical procedures have been usually conducted on different versions of MATLAB, Excel, and SPSS software.

Exploiting smartphone applications for monitoring or displaying the obtained data was another investigated feature in the studies of this review. To provide a better experience for subjects or patients, especially for work environments in which subjects might not have easy access to computers or displays, developing or using a smartphone app can significantly improve the quality of monitoring or displaying the inertial data [118]. Moreover, through smartphone applications, various forms of feedback such as auditory, text, and vibrations can be provided for users to notify them regarding their risky postures or correct/incorrect motions [118]. Considering the significant technological advancements in smartphones, developing a smartphone application can raise the quality of supervision and connection of health care providers, clinicians, and researchers with individuals. Therefore, including a smartphone application in their wearable system is highly recommended for future applications considering its current low rate of utilization in UE-MSK conditions (n = 10, 13%).

As can be understood from the results of this scoping review, a diverse set of equipment, setting, processing and intervention has been applied in studies that used wearable sensor systems. No globally accepted standard method for sensor placement, hardware characteristics, intervention guidelines for wearable systems, extracting features from the inertial signal, or categorizing activity across important functional motions has been proposed. In accordance with findings of Dobkin and Martinez [112], and Attal et al., [119], this can be considered as one of the most important limitations of this field.

The findings of this scoping review are limited to the included studies and synthesized pieces of found evidence, and this is one of the limitations of this review. Conducting a broad search with other keywords (for example, including different types of MSK disorders like arthritis and carpal tunnel syndrome in the search process keywords) and database might result in finding other studies that alter the interpretation of this review’s results. Nevertheless, in this scoping review, it has been attempted to include all necessary keywords to yield all related papers. While other search strategies might lead to different results. Other available sources have been also searched and examined to acquire all available pieces of evidence in the field. One of the other points that should be noted when reading our scoping review is that we did not consider the statistical analysis/results, including details about algorithms and performance metrics. However, we would like to clarify that the primary focus of this scoping review is on wearable sensor systems and their corresponding hardware. Given the breadth and depth of the topic, we made a deliberate decision to exclude detailed statistical analyses and results to keep the manuscript concise and in alignment with our specific research scope. We believe this approach allows us to provide a clear and coherent overview of the wearable sensor technologies and their applications.

## Conclusion

Wearable sensors are increasingly applied in UE-MSK related studies. They prove to be significantly important in the development of the next generation of health care technologies in the assessment and treatment fields. They can be utilized for clinical assessments, home-based applications, daily activity recording of patients and individuals or non-standardized areas such as work environments to characterize the physical risk factors of developing UE-MSK conditions or investigating the ergonomic risk levels of various work environments. A large share of reviewed papers are observational studies that shows the need for conducting more robust research studies from the aspect of study design to yield more valid results. IMU sensors were primarily applied in most of studies (n = 68), and sEMG sensors were included in wearable platform system of some papers to obtain inertial data and muscle activity (n = 13), respectively. The share of assessment-oriented studies is greater than treatment-oriented papers that represents the current assigned primary role of wearable sensors in obtaining objective upper body motion data. Based on this review’s findings and current evidence, conducting randomized clinical trials using wearable sensors system and research about novel treatment applications of wearable sensors, focusing on home-based applicability of suggested systems, the inclusion of a variety of sensors such as user-friendly EMG and bend sensors in addition to IMUs, and designing smartphone applications for convenient and continuous monitoring of users are recommended for future studies of this field.

### Supplementary Information

**Additional file 1.** Appendix A.

**Additional file 2. Table 1**: Clinical studies. **Table 2**: Work-related Musculoskeletal conditions. **Table 3**: General wearable studies and designs.

## Data Availability

All data generated or analyzed during this study are included in this published article (and its supplementary files).
