# Supplementary material for: Applications of wearable sensors in upper extremity MSK conditions: a scoping review
Source: J Neuroeng Rehabil. 2023 Nov 18;20:158. doi: 10.1186/s12984-023-01274-w (PMC10656914; doi:10.1186/s12984-023-01274-w)
Supplement: Supplementary file 1 — Additional file 1. Appendix A. [file 12984_2023_1274_MOESM1_ESM.docx]

Appendix A

Scopus: 957 articles

( TITLE-ABS-KEY ( "upper extremity" ) OR TITLE-ABS-KEY ( "upper limb" ) OR TITLE-ABS-KEY ( "shoulder" ) OR TITLE-ABS-KEY ( "hand" ) OR TITLE-ABS-KEY ( "wrist" ) OR TITLE-ABS-KEY ( "elbow" ) OR TITLE-ABS-KEY ( "arm" ) OR TITLE-ABS-KEY ( "forearm" ) ) AND ( TITLE-ABS-KEY ( "IMU" ) OR TITLE-ABS-KEY ( "inertial measurement unit*" ) OR TITLE-ABS-KEY ( "wearable sensor*" ) OR TITLE-ABS-KEY ( "wearable electrode*" ) OR TITLE-ABS-KEY ( "instrumented garment*" ) OR TITLE-ABS-KEY ( "smart prosthesis" ) OR TITLE-ABS-KEY ( "wearable device*" ) OR TITLE-ABS-KEY ( "wearable electronic*" ) OR TITLE-ABS-KEY ( "inertial sensor*" ) ) ANoD ( TITLE-ABS-KEY ( "musculoskeletal" ) OR TITLE-ABS-KEY ( "MSK" ) OR TITLE-ABS-KEY ( "MSD" ) OR TITLE-ABS-KEY ( "muscle*" ) OR TITLE-ABS-KEY ( "bone*" ) OR TITLE-ABS-KEY ( "tendon*" ) OR TITLE-ABS-KEY ( "ligament*" ) )

IEEEXPLORE: 296 articles

(( (("upper extremity") OR ( "upper limb" ) OR ( "shoulder" ) OR ( "hand" ) OR ( "wrist" ) OR ( "elbow" ) OR ( "arm" ) OR ( "forearm" )) AND ( ( "IMU" ) OR ( "inertial measurement unit" ) OR ( "wearable sensor*" ) OR ( "wearable electrode*" ) OR ( "smart prosthesis" ) OR ( "wearable device" ) OR ( "wearable electronic*" ) OR ( "garment" ) OR ( "inertial sensor*" )) AND (( "musculoskeletal*" ) OR ( "MSK" ) OR ( "MSD" ) OR ( "muscle*" ) OR ( "bone*" ) OR ( "tendon" ) OR ( "ligament" ))))

PubMed: 168 articles & Embase: 223 articles [Using Ovid]

1 Musculoskeletal*.tw,kf.

2 MSK*.tw,kf.

3 MSD*.tw,kf.

4 bone*.tw,kf.

5 muscle*.tw,kf.

6 tendon*.tw,kf.

7 ligament*.tw,kf.

8 or/1-7

9 upper extremity/ or arm/ or axilla/ or elbow/ or forearm/ or hand/ or fingers/ or metacarpus/ or shoulder/ or wrist/

10 upper limb*.tw,kf.

11 9 or 10

12 Wearable Electronic Devices

13 Wearable sensor*.tw,kf.

14 IMU*.tw,kf.

15 Inertial measurement unit*.tw,kf.

16 inertial sensor*.tw,kf.

17 smart prosthetic*.tw,kf.

18 wearable device*.tw,kf.

19. instrumented garment*.tw,kf.

20 or/12-19

21 8 and 11 and 20
