# Supplementary material for: Applications of wearable sensors in upper extremity MSK conditions: a scoping review
Source: J Neuroeng Rehabil. 2023 Nov 18;20:158. doi: 10.1186/s12984-023-01274-w (PMC10656914; doi:10.1186/s12984-023-01274-w)
Supplement: Supplementary file 2 — Additional file 2. Table 1: Clinical studies. Table 2: Work-related Musculoskeletal conditions. Table 3: General wearable studies and designs. [file 12984_2023_1274_MOESM2_ESM.docx]

**Table-1: Clinical studies**

| Author- year | Interventions or data collection methods | Measured outcome(s)  [secondary outcomes are indicated with ‘secondary’] | Important points of outcome processing method |
| --- | --- | --- | --- |
| **(1)**  **Duc. et al.- 2014** | (1) In the laboratory settings, subjects performed 90° arm abduction, 90° arm flexion, 40° arm extension, and arm external rotation, (all performed in both states of extended elbow and flexed elbow at 90°.  (2) Subjects’ daily activities were recorded for 7h duration. | (1) Duration of humorous movements (s)  (2) Duration of muscular activation (s) in medial deltoid and biceps brachii | (1) Humerus movement was detected when its angular velocity norm exceeds a specific threshold, and larger than the thorax angular velocity norm. The activation duration is recorded (T_mov_).  (2) Muscle activity duration (T_EMG_) is calculated when the EMG signal exceeds the specified threshold (average+ 3*the standard deviation of the baseline activity at rest posture).  (3) The ratio of these durations is defined as the relative time of muscular activation. |
| **(2) Pichonnaz et al. -2015** | (1) Subjects recorded a minimum of 7 hours of their daily routine (walking, sitting, and standing postures). | (1) Arm activity usage (%) | (1) Active duration of arm was defined in time sample that the product of acceleration and angular velocity (movement power), exceeds the threshold corresponding to the mean power of 1 hour recorded data on a motionless person.  (2) The mean of the group of healthy subjects having similar dominant side characterized the expected arm usage. The outcome was defined underuse in case of obtaining a negative difference between the measured surgical side usage and the expected arm usage (measurements were performed at 3, 6, and 12 months). |
| **(3)**  **Duc. et al.- 2013** | (1) Laboratory: Subjects changed the places (up-down and left-right) of 1.5-liter bottles and pens while standing.  Daily life activity: Subjects recorded a minimum of 7 hours of their daily routine (walking, sitting, and standing postures). | (1) The movement frequency of arms (number of arm movements/ the number of sitting and standing hours)  (2) The symmetry index of movement frequency in dominant and non-dominant arms (%)  [Using duration of movements (h) and angular velocity of arms (°/s)] | (1) Arm movements were recognized, when the angular velocity of the humerus was larger than the angular velocity norm of the trunk.  (2) The arm usage periods were defined using movement  frequency (Fr) that is obtained by dividing the number of detected arm movements and the number of hours of sitting and standing  postures. Also, the symmetry index of Fr in dominant and non-dominant arms (SIFr) was obtained based on the Fr in dominant and non-dominant arm.  (3) The quality of arm usage was assessed by comparing the arm  velocity distribution of the individual patient to that of the control group using angular velocity norms and the empirical cumulative distribution function of velocity in control group and intervention group subjects. |
| **(4)**  **Najafi, B et al. -2021** | (1) Subjects began the movement task in the state of 90° abductions with 90° elbow flexed, and performed 180° arm abduction with elbow extension to the  “stick-up” position and then returned to initial state. This movement is repeated as fast as possible for 20 s. | (1) Joint angle, and angular velocity in 3 axes (yaw, pitch, roll; °, and °/s).  (2) Number of performed cycles (#)  (3) Duration of abduction and adduction movements (ms) | (1) Processing and feature extractions were conducted by MATLAB software (Mathwork, Inc., Ver R2018a).  (2) 18 Kinetic and kinematic features were obtained from 5 categories of motor capacity (slowness, weakness, rigidity, exhaustion, and unsteadiness) and 6 features of speed (°/s), abduction time (ms), abduction/adduction time (ms), number of performed cycles (#), power (deg^2^/s^3^), and range of motion (°) were meaningful in discriminating affected-and unaffected extremities. |
| **(5)**  **Van de Kleut. et al.- 2021** | (1) Subjects performed daily activities for a day with two follow up sessions at 3 months and 12 months after the operation (mean wear time= 7.1 hour) | (1) Number of elevation events/h (2) Percentage of time spent within different elevation ranges of 0◦-20◦, 20◦-40◦, 40◦-60◦, 60◦-80◦, 80◦-100◦, and >100◦ (%)  (3) The percentage of occurred elevation events within the elevation ranges (%)  (4) Intensity of arm activity (low, moderate, or high)  [using arm joint angles in 3 axes (yaw, pitch, roll; °)] | (1) Data processing was performed in MATLAB, and orientations of arm joints has been determined using real-time Kalman filtering.  (2) IMU data of sensors were time matched, and rotational difference was obtained with respect to the torso. Through this, the anatomic joint angles and angles of shoulder elevation were acquired.  (4) Elevation events were counted when a minimum peak height of 20◦ and width of 1 second occurred.  (5) Intensity of arm activity were defined by sorting the number of elevation events in a 60s epoch in 3 categories. |
| **(6)**  **Kwak et al.- 2019** | (1) Performing forward elevation for 2 times and for each arm while subjects were seated (the move was instructed by a video) | (1) The number of peaks in the sum of angular velocity  (2) The peak velocity–to–mean velocity ratio (ratio of the maximum recorded velocity of the motion and the mean velocity)  (3) The number of sign reversals in angular velocity (number of zero crossings)  [Using angular velocity and acceleration of arms (°/s, g).] | (1) Data processing was performed in MATLAB, R2015a (MathWorks, Natick, MA, USA), using angular velocity and acceleration of the arm movement. The intact arms have been compared with shoulders with rotator cuff tears.  (2) The number of peaks in the sum of angular velocity, the peak velocity–to–mean velocity ratio (ratio of the maximum recorded velocity of the motion and the mean velocity), and the number of sign reversals in angular velocity (number of zero crossings) were considered the motion smoothness parameters. |
| **(7) Larrivée et al.- 2019** | (1) Subjects performed their daily activities for 5 weeks from morning to bedtime that is the data recording duration (the measurement and follow-up times were the week before the CIS, the day of CIS, and 2 and 4 weeks after CIS). | (1) Active time; reported as a ratio with total recording time (ratio)  (2) Mean activity count (AC) per minute (ratio) sorted in 3 categories of low-intensity  (LIA), medium-intensity (MIA), and high-intensity activities (HIA)  [Using 3 axes acceleration data (g)]. | (1) Data Processing was conducted by low-pass filtering (Butterworth, 1 Hz, 2nd order) full-wave rectifying and high-pass filtering (Butterworth, 5 Hz, 2nd Order) the accelerometer data for removal of sensor noise and gravitational acceleration vector.  (2) The “active time” periods of acceleration data were defined in time samples, where 50% of the data values over a 10-second window were larger than 0.015 g threshold.  (3) Activities with an AC below the 33^rd^/66^th^/100th percentile of all activities recorded were classified as low, medium, and high intensity activities, respectively. The two thresholds separating these three activity levels are an AC of 90.0 and 180.0 (for low to medium and medium to high, respectively).  (4) Number of times that subject forgot to wear or operate the system have been acquired by a short questionnaire at the end of study. The percentage of missing days over the total number of participant-days have been considered as compliance and reliability. |
| **(8)**  **Burns et al.-2018** | (1) Performing 7 shoulder exercises of an evidence-based rehabilitation protocol for full thickness atraumatic rotator cuff tears including pendulum (PEN), abduction (ABD), forward elevation (FEL), internal rotation (IR), external rotation (ER), trapezius extension (TRAP), and upright row (ROW). IR, ER, and TRAP movements were performed with a medium resistance band, and ROW was made with a 3 lb weight. | (1) Acceleration and rotational velocity in all 3 axes (yaw, pitch, roll; g, and °/s) | (1) Activity recognition was processed and conducted in seglearn software (an open-source Python package).  (2) Class labels (exercise type) were assigned to the raw data after feature extraction and segmentation of corresponding data. Through a semi-autonomous algorithm, the inactive portion of data was discarded using thresholding the 2 s moving average of the accelerometer signal energy.  (3) 4 Supervised learning algorithms were trained and optimized for exercise classifications including: k-nearest neighbors, random forest, support vector machine, and a convolutional recurrent neural network. |
| **(9)**  **Burns et al.- 2020** | (1) Subjects participated in physiotherapy exercise sessions wearing the smart watch, up to a maximum of 5 months.  (2) The exercise type and technique labels were recorded every 2 weeks synchronously with inertial data collection (the technique will be labeled as a binary variable of correctly performed or not).  (3) A record of prescribed home exercises type, technique evaluations at each session were made. | (1) Participation adherence rate (%) over each 2-week interval of treatment  (2) Shoulder active range of motion in 3 axes (yaw, pitch, roll; °) | (1) Sensor data were divided into fixed-length durations (2-4 s) learned by a deep convolutional recurrent neural network (CRNN) classifier. After training, the classifier reconstructed a record of activities from the inertial sensor data.  (2) Trained algorithms generate a record of exercises performed from inertial sensor data collected during unsupervised home sessions and supervised sessions, and by comparison of the quantity and frequency of home exercises with the prescribed exercises participation adherence parameter can be obtained. |
| **(10)**  **Hurd et al.-2018** | (1) Subjects performed their daily activities from morning till night at a 3-day period preoperative, after 2 months and after 1 year from the surgery. The subjects were trained for using and removing the wearable system at home. | (1) Mean limb activity (m/s^2^/epoch)  (2) The activity frequency categorized in 3 levels of inactive, low, and high (%)  [Using 3 axes accelerometer data (g)] | (1) The raw inertial data were processed in self-developed MATLAB software, high-pass filtered (at 0.1 Hz), and the result was segmented into 1-min epochs  (2) Rach epoch was assigned a single activity value by summing the vector magnitude from the 3 axes, and mean activity value was calculated for each segment.  (3) Activity frequency was categorized into 3 levels:  (a) Inactivity: percentage of [activity value < 110 m/s^2^/epoch].  Low/High activity: maximum activity (MA) was determined from a matched control group, and after performing normalization:  (b) Low activity: percentage of [110 m/s2/min epoch ≤ Activity value t ≤ 33% of MA]  (c) High activity: percentage of [Activity value t > 33% of MA] |
| **(11) Ajcevic et al.-2020** | (1) The physical therapy was organized in 15 one-hour sessions (in three months period) including performing  (a) micro-mobilization of joints, (b) combined multidirectional mobilization  technique in the gleno-humeral joint, and (c) counter-resistance mobilizations with post-isometric release in anterior flexion, abduction, external rotation and internal rotation and postural active exercises. | (1) The range of motion in elevation, and abduction movements of scapula and humerus (yaw, and pitch; °)  (2) Activation time of the scapula and humerus (s) | (1) Calibration is performed by a static measure with the subject standing in a pre-determined posture and the calculation of the anatomical coordinate systems: upright position, elbow flexed at 90°. |
| **(12)**  **Chen et al.-2020** | (1) Calibration: placing the sensor on a horizontal fixture for measurement of each axis relative offset correction (the original offset of the sensors to each other)  (2) Measurements are performed for shoulder abduction, flexion, extension, external rotation, and internal rotation.  (3) For reliability, the active ROM (aROM) and passive ROM (pROM) are assessed using the motion sensor device and then by two examiners using a universal goniometer.  (4) The aROM involves measurement of subjects moving their arm as far as they could, and the pROM involves measurement of movements of subject’s arm to its mechanical limit or a limit imposed by pain performed by examiner.  (5) For effectiveness, home-based group exercises included Codman’s or pendulum exercises (circumduction) and passive stretching exercises holding the position for 10 s (10 repetitions), and for the motion sensor–assisted rehabilitation group exercises that were received on operating the motion sensor device and Patient App with detailed instructions. | (1) Active ROM and passive ROM of shoulder (yaw, pitch, roll; °)  (2) Exercise completion rates (%) | (1) The raw data were converted into a quaternion algorithm to convert the relative angle changes of the upper arm and wrist sensors into a 3D motion of the shoulder.  (2) The patient smartphone app (developed by BoostFix) provided modes for measuring shoulder mobility, generating historical records of angular measurements and exercise completion rates, and providing daily shoulder exercises including a forward wall walking stretch, lateral wall walking stretch, and cane stretch for shoulder flexion, extension, abduction, internal rotation, and external rotation presented by a 3D avatar.  (3) Doctor smartphone app (developed by BoostFix) provides the latest shoulder ROM measurements of subjects and exercise completion rates for the previous week.  (4) Physiotherapists and physicians can assign personalized daily home-based exercises with adjustable targeted angles, numbers of repetitions, and holding times for each patient based on the angular status of the affected shoulder, and they can be in contact with patients through text messages provided in the app. |
| **(13)**  **Aslani et al.-2018** | (1) A gimbal test stand simulation was designed for quantifying the IMU sensor performance.  (2) Arm elevations were performed medially, then anteriorly, cranially, posteriorly, laterally, and then back to the initial rest position (3 repetitions for evaluating the repeatability of the test). | (1) Shoulder range of motion in Spherical coordinate (azimuthal angle and elevation angle)  Reference point: Top shoulder joint  (2) RMS values of EMG activity of muscles (V) | (1) ATmega microcontroller filtered, rectified, and processed the raw EMG signal. Afterward, it synchronizes EMG and IMU sensors data.  (2) A self-developed MATLAB software has been designed for processing purposes, and the data is represented through an animated figure and graphs.  (3) The orientation quaternion vector is calculated by microcontroller and through mathematical equations the obtained rotation matrix of arm is converted to a cartesian coordinate system and finally to a spherical coordinate. |
| **(14)**  **Yin and Xu-2018** | (1) Subject raises the arm to control height of the aircraft in game. The actions resemble the wall climbing exercises. | (1) Joint elevation angles in all 3 axes (yaw, pitch, roll; °) | (1) The motion sensitive game was developed with Microsoft® XNA game engine.  (2) The game console stores the game’s scores, arm angles and bonus items into the server.  (3) The stored data are assessed to evaluate the patient’s recovery by completing the training tasks. |
| **(15) Xuedan et al.-2019** | (1) No information has been provided regarding the optimum duration. The proposed system is equipped with basic display, control, and pulse emission functions, enabling to provide a variety of treatment options. | (1) Peak optical power (W)  (2) peak optical density (mW/cm^2^) | (1) The treatment occurs by emitting optical Pulse-Width Modulation (PWM) signals (ON time= 50us and OFF time=800ms, duty cycle ~1/16000) from LEDs.  (2) The average optical power is measured with a Newport Model 843-R Optical Power meter. |
| **(16) Körver et al.-2014** | (1) The measurement performed at the outpatient clinic and directly after diagnosing impingement syndrome. Subjects sat on a non-rotating stool with 90◦ of hip, knee, and ankle flexion, and performed two motion tasks of (a) and (b) with <5 min duration and 3 repetitions. They held the position for 2s and returned to neutral position.  (a): Subjects touched the belt indicator with their hand palm starting from neutral anatomical position of arm.  (b): Subjects reached their hand palms to their occiput with the elbow straight out to the side starting from neutral anatomical position. | Upper arm accelerations and angular rate (velocity) in 3 axes (yaw, pitch, roll; g, and °/s) | (1) The data is processed in 3DM-GX2 Software Development Kit, and the algorithms obtain the asymmetry in shoulder movements between both sides.  (2) Angular rate (AR) score of shoulders that is the average of the sum of the peak-to-peak differences in the angular rate signal for all 3 axes is calculated. Asymmetry AR-scores and relative asymmetry AR-scores with respect to healthy subjects scores are also obtained.  (3) Higher asymmetry values represent increasing asymmetry in shoulder kinematics, indicating a probable cause for unilateral deteriorated shoulder movements or by a highly developed shoulder movement. |
| **(17) Lorussi et al.-2019** | (1) Subject perform gamified moves based on the movements of the presented 3D avatar in the application. The moves include concentric and eccentric arm abduction at different angles. | (1) Shoulder and wrists joint angles in 3 axes (yaw, pitch, roll; °) with respect to standing position reference  (2) The measurements error of software in 3 axes (yaw, pitch, roll; °) | (1) Self-developed software presents the measurements data.  (2) Through a previously proven valid model that uses the movements of the scapular-thoracic complex and gleno-humeral joint and constraint due to the scapular-humeral rhythm, a mathematical equation considers various subject-specific parameters along with obtained abduction and horizontal flexion angles of the arm detected by the wrist IMU with respect to the sternum.  (3) The performed move is replayed through another 3D avatar for the subject and clinician for evaluation purposes, and the quality of subjects’ movements is assessed by the application (4) Clinicians can design specific exercises including concentric, eccentric, and isometric movements that several parameters of them such as the speed of motion, holding time, amplitude and the index referring to the actual position can be modified. |
| **(18) Carmona-Ortiz et al.-2020** | (1) Calibrations were performed by recording the drift of sensors for 12.5 h and wearing the system for 4.5 h by healthy subject during his daily life and periodically repeat the static N-pose for assessment of reliability.  (2) Both subjects wore the ArmTracker device 7 hours (approximately from 10 a.m. to 5 p.m.) doing their daily life activities. | (1) Arm and wrists joint angles and acceleration (yaw, pitch, roll; °, g) | (1) A self-developed MATLAB processed the data  (2) Joint angles, positions, and acceleration data were used for analyzing joint ranges of motion, subject’s functional workspace, and actimetry metrics, respectively.  (3) ArmTracker and the Xsens systems recordings were made during elbow flexion/extension and forearm pronation/supination movements for validation purposes.  (4) The quality of the upper limb movements was assessed by obtaining range of motion, functional workspace distribution (the percentage of time spent of the subject’s hand in 8 determined regions), and actimetry metrics (unilateral activity, bilateral magnitude, and magnitude ratio). |
| **(19) Zucchi et al.-2020** | (1) The subjects sat up straight, with the examined arm fixed by an elastic band.  (2) After an anatomical calibration, flexion (FL)-extension (EX), radial (RD), and ulnar deviation (UD) were performed by subjects, next movements were pronation (PR) and supination (SU) performed by subjects with IMU positioned on the dorsal surface of the radio with the forearm in a neutral position (4 repetitions).  (3) For EMG recordings and for each muscle, 4 isometric contractions were performed, each lasting 30s, and maximal voluntary contractions (arms in adduction position, elbow flexed at 90°, and with forearm in a neutral position). | (1) Affected wrist ROM (°) and in percentages with respect to the unaffected wrist (%) through ulnar and radial deviation (yaw), flexion  and extension (pitch), and pronation and supination  of forearm  (2) EMG signal (V) | (1) Both IMU and EMG systems provide their own software for processing and presenting the data (Fisio computer software provides real time 3D kinematics of wrist).  (2) For each trial, the maximal values of FL, EX, RD, UD,  PR, and SU were obtained in both affected and unaffected wrist.  (3) An RD or UD value higher than 5° represents the presence of ulnar or radial compensation, respectively.  (4) EMG data was bandpass filtered at 10–500 Hz.  (5) Time-frequency transformation was performed using Discrete Fourier Transform for analyzing shifts in instantaneous spectrum median frequency (SMF) between each isometric contraction (subdivided into intervals that last 1s). The percentage decrease in the median frequency represents muscle fatigue. |
| **(20) Perraudina et al.-2018** | (1) Subjects wore sensors for all day (for 4 weeks) and performed STS test (5 repetitions) at their home in the morning 3 times per week.  (2) Researchers taught the subjects correct performing of the STS test, and subjects completed a test under their supervision in the beginning and at the end of the study.  (3) STS test consists of 5 cycles of standing and sitting, with arms crossed over the chest. | (1) The 3-axis accelerometer data (g), and its spherical coordinates (norm vector, azimuth in degrees, elevation in degrees)  (2) The duration of the 5×STS tests (s)  (3) Adherence to the tests (%) | (1) The sensor devices data were periodically collected in an aggregated format (1 sample/min), and the percentage of wear time was obtained.  (2) The data set was divided into development (24 subjects), test (11 subjects), and validation data sets (10 subjects). The development datasets selected the model, the test datasets confirmed the model, and the validation datasets assessed the generalizability to other subjects. 5 days data of each subject was added to validation dataset for subject-specific model validation.  (3) The cartesian coordinates (x, y, z) data gathered by accelerometer were converted into spherical (r, azimuth, elevation) coordinates, where r is the norm value of the acceleration, azimuth is the accelerometer rotation angle and elevation are the tilt relative to the plane of the device/watch face.  (4) The main variable of the built model is the duration of the 5×STS tests. Other covariates like age, gender, BMI, seconds since getting up, and disease type that associate with pain and stiffness were also considered. |
| **(21) Kassanos et al.-2019** | (1) No procedure | (1) Temperature of element placed on wrist (°c) | (1) Two copper meandering tracks parallel to each other with a copper thickness of 35 μm represented the Joule heating element and temperature sensor can cover an area of 60 mm x 26 mm on the wrist.  (2) Heater element operates linearly by inducing direct current of electricity (limited to raise the temperature within 30°c to 50°c). |
| **(22) Murad et al.-2017** | (1) The subject performs nine hand gestures including flick up, flick down, flick left, flick right, forward, backward, clockwise, counterclockwise, and swipe down. Each of the gestures are mapped into a specific sound enabling to compose a musical piece. | (1) 3 Axes accelerometer signals (yaw, pitch, roll; g) | (1) The artificial neural network is used for classification of gesture recognition (raw accelerometer values across 3-axis as input).  (2) Sudden changes in accelerometer values are defined as occurrence of a gesture (obtained by using Euclidean distance).  (3) 6 Features of mean, standard deviation, variance, minimum and maximum values, and dynamic time wrapping are obtained for training the neural network. |
| **(23) Holland et al.-2020** | (1) Subjects were instructed to place a finger sensor onto the center of the load cell with maximum contact of a fingertip with the load cell as comfortably possible, and gradually increase the force applied to the load cell, in a  linear fashion, until they reached a set average threshold of 12.5±2.5 N.  (2) Regular golf grip strength and maximum golf grip strength of subjects were obtained.  (3) Each subject performed two swings, hitting a golf ball off artificial turf into a net with all 12 grips.  (4) A camera perpendicular to the joints records the subjects grip positions during the trial with reflective markers. | (1) Forces at the distal palmar  aspect of the thumb, index, middle, and ring finger of each participant’s trail hand (Pounds per square inch or psi)  (2) Grip configuration or ROM of the thumb and index fingers (°) | (1) The Dartfish Movement Analysis Software evaluates the small angle measures of the thumb and index using the videos and 2D reflective markers. Further grip force data and video-based data have been processed through a self-developed MATLAB 2017b software (The MathWorks Inc., USA) script.  (2) A single degree of freedom load cell (by Pressure Profile Systems Inc.) converts each individual sensor output from capacitance to force.  (3) Maximum forces of the thumb, index, middle, and ring finger were separately averaged for each swing, and then an overall golf grip force was obtained by summing these average values for each activity. Finally, by normalizing the activity’s total maximum applied grip force values to the individual test group’s maximum golf grip force, the percentage of grip force during a golf swing with respect to the maximum golf grip strength is obtained.  (4) Chameleon Visualization Software by Pressure Profile  Systems Inc. is used for obtaining the force and pressure data. |
| **(24) Silişteanu et al.-2016** | (1) At first the calibration process was performed on a normal hand and the values of the possible angles for each joint were set.  (2) The data were used to consider the angular limit values to not worsen the condition of subjects with Carpal tunnel syndrome. | (Secondary)  (1) The positions of fingers (no information regarding the details) | (1) The system obtains the fingers movements through optical fibers making a loop near each joint. The execution angle of the joint movement is acquired by measuring the intensity of the reflected light. |
| **(25) Connolly et al.-2018** | (1) After initial calibration, subjects performed a protocol of finger flexion and extension exercises (repeated for both gloves and recorded using self-developed software). | (1) ROM and angular velocity of finger joints (yaw, pitch, roll; °, °/s) | (1) Each IMU sensor detects inclination and orientation of individual finger phalanx’s relative to local hand position (not to gravity) by subtracting overall inclination of the hand relative to IMU’s on each phalanx from the IMU placed at the back of the hand.  (2) All IMU sensors are sampled before beginning of movements (hand in a neutral position) to calculate and remove finger joint thickness and slope offset.  (3) Through a mathematical filter accelerometer and gyroscope data are combined to obtain rotation angle, and angular velocity.  (4) Self-developed user interface software presents the angular output displayed by a 3D model of hand and identifies variations and deterioration in movement caused by joint stiffness. |
| **(26)**  **Mack and min-2019** | No information | Wrist angle (pitch, °) | (1) Self-developed GUI software using MATLAB script enabling data processing, data storage, and detecting hand-wrist positions.  (2) Bending increases the resistance value of the sensor (“flat” sensor resistance is 8 kΩ, and around 20 kΩ when flexed to 90°). Then, it is translated into voltage sensed by the microcontroller and converted into angle through simple mathematic equations and look-up table of sensor |
| **(27) O’Quigley et al.-** | (1) Samples were prepared from the LTT and LR fabric, with and across the knit (4 samples) with attached cooper tapes at each end of them serving as physical and electrical connection.  (2) Each sample was moved by a sinusoidal wave at 0.25 Hz for 20 cycles of 1mm - 5mm amplitude, with the total stretch, from resting state, equal to twice the amplitude, and another test with similar parameters for 1200 cycle. | Finger joint angles (°) | (1) Self-developed GUI presents a real-time virtual representation of the hand.  (2) Arduino sends the sensed voltage signal onto a computer and the results are transformed into resistance values in a spreadsheet using a certain equation and further into angles.  (3) Obtained results are compared with the gold standard Vicon Nexus motion capture system (Infrared reflective markers are placed on the fingers). |
| **(28)**  **Langohr et al.-2018** | (1) The subjects’ ROMs were assessed using a long-arm goniometer  (2) Subjects wore the motion-tracking garment and asked to assume a "tin soldier" position, which was defined as 0° of abduction and internal-external rotation. The sensors were activated, and the patient was asked to perform a series of motions throughout maximum ROM.  (3) Subjects leave the lab and continue their daily activities until the end of the day. Moreover, they were asked to make a brief log activity for the detection of any abnormal pattern. | (1) ROM and shoulder joint angles (°)  (2) Percentage of time spent in different angle ranges of elevation and plane of elevation axes (%)  (3) Number of motions per hour in each angle range | (1) All patients were at more than one-year postoperative status at the time of inclusion in the study  (2) They had primary arthroplasties operation, lived independently, and were fully ambulatory without aids  (3) The LabVIEW developed GUI represents the recorded angles of joints at each time point.  (4) Peaks and valleys of shoulder motion in different axes were obtained and for filtering the occurred minute motions, a motion threshold of 10° was set (passing this threshold would be identified as a shoulder motion). |
| **(29)**  **Haverstock et al.- 2020** | (1) The subjects’ ROMs were assessed using a long-arm goniometer  (2) Subjects wore the motion-tracking garment and asked to assume a "tin soldier" position, which was defined as 0° of abduction and internal-external rotation. The sensors were activated, and the patient was asked to perform a series of motions throughout maximum ROM.  (3) Subjects leave the lab and continue their daily activities until the end of the day. Moreover, they were asked to make a brief log activity for the detection of any abnormal pattern. | (1) ROM and forearm joint angles in flexion/extension and pronation/supination postures (°)  (2) Percentage of time spent in different angle ranges (%)  (3) Number of elbow motions per hour in each angle range | (1) The accuracy of the kinematic shirt was obtained 4°  ± 3° compared with a simultaneous passive optical tracking recording system  (2) The LabVIEW developed GUI represents the recorded angles of joints at each time point.  (3) Peaks and valleys of elbow flexion-extension and pronation-supination were obtained and for filtering the occurred minute motions, a motion threshold of 10° was set (passing this threshold would be identified as an elbow motion). |
| **(30)**  **Lavado and Vela- 2022** | (1) Subjects performed a complete isometric submaximal elbow flexion and extension during a trial of 10 seconds for a total of 5 times. The rest period between each trial was 2 minutes.  (2) Joint angles were simultaneously obtained through analyzing optical markers and the video recordings using a GoPro Hero 7 camera to validate the inertial sensors angles. The video recordings are processed using the Kinovea software. | (1) ROM and flexion-extension elbow joint angle (°)  (2) EMG amplitude of biceps brachii and triceps brachii (V) | (1) The GUI presents the angular position of the elbow joint angle measured by the IMU sensors, and the EMG signals obtained from the biceps and the triceps muscles. In the Range of Motion section of the GUI, a physician can enter the allowed angle range for the patient's therapy session.  (2) The WEMOS D1 mini Wi-Fi module was used for wireless transmission of data that allows Wi-Fi connection to be established through the wireless transmission protocol TCP/IP  (3) A 60-Hz notch filter, a second-order Butterworth low-pass filter with 150 Hz cut-off frequency and a second-order Butterworth high-pass filter with 20 Hz cut-off frequency were implemented. The sampling frequency of the EMG signal was 1 kHz.  (4) The inertial data of Y and Z axes in the accelerometer and the X axis of gyroscope were responsible for calculating the elbow joint angle. |
| **(31) Rigozzi et al.- 2022** | (1) Subjects initially completed a guided 7- minute warm-up of hitting forehands cross-court at different spin levels into a target zone.  (2) For the Testing Phase, subjects hit 40 forehand strokes cross-court following the height guideline into the target zone for each forehand spin level. There was 15-seconds rest between each series and a 5-minute rest between each spin level.  (3) For obtaining maximum voluntary contraction, subjects maximally griped the racket using their normal forehand grip position for 5 seconds in a comfortable seated position with a 2-minute rest between each trial | (1) Normalized EMG activity and grip strength to the maximum voluntary contraction (MVC) activity level (%)  (2) Angular rotation of racket, wrist, and elbow (°/s) | (1) The MATLAB software is used for processing both EMG and accelerometer data.  (2) The timestamps (beginning and ending times) associated with each successfully hit shot were manually extracted from the original file through a threshold of 20g on the ICM20649 z-axis accelerometer sensor. When this threshold was reached, time zero was established and a half-second of data was stored on each side.  (3) A Butterworth Filter order 8 (high-pass with 10 Hz cut-off frequency and a low- pass with 10 Hz cut-off frequency) was applied to the ECR and FCR data. |

(ROM: Range of motion; sEMG: Surface electromyography; V: Volt; W: Watt; s: Second; ms: Millisecond; °: Degree; m: Meter; cm: Centimeter; °c: Degree Celsius; Hz: Hertz; g: g-force [Unit of acceleration])

**Table-2: Work-related Musculoskeletal conditions**

| Author- year | Interventions or data collection methods | Measured outcome(s)  [secondary outcomes are indicated with ‘secondary’] | Important points of outcome processing method |
| --- | --- | --- | --- |
| **(1)**  **Humadi et al.-2020** | (1) Three manual material handling tasks (6 repetitions each). Including (a) trunk twisting to pick up an object from the initial position, (b) an asymmetric movement in which subjects bent in the sagittal plane to the knee  height to pick up a box and place it on another desk (higher height), and (c) the subjects stand motionless (N-pose) for five seconds and then reach to an object at the head height. Each repetition lasts about 15s. | (1) Root-mean-square error (RMSE) of obtained joint  angles in both methods with respect to obtained joint angles by VICON system as the gold standard (°).  (2) RULA score (1 to 7)  [Using 3 axes upper arm, lower arm and elbow joint angles (yaw, pitch, roll; °)] | (1) Self-developed software and hardware were utilized for obtaining and synchronizing the data of IMUs, and motion-capture system.  (2) The IMU data were up sampled to 100Hz to reach equal sampling frequency of camera system.  (3) The IMU-based segmental kinematic 3D model is achieved by the Xsens-MVN Analyze software (Xsens Technologies, The Netherlands).  (4) The obtained data of motion cameras are converted into joint angles through simple mathematic equations and compared with IMU-measured angles to obtain RMSE of both methods at each joint.  (5) The obtained upper arm and elbow joint angles were used in calculating the sub-scores of the RULA method for each segment at each time sample. |
| **(2)**  **Humadi et al.-2021** | (1) Manual material handling tasks include (a) packing (twisting the body for picking up an imaginary object from a table and put it on another table, (b) loading (bending and twisting for picking up an imaginary object from a cart and placing it on a table), (c) picking up (picking an imaginary object from the ground), (d) package inspection (a symmetric movement to bend forward to the knee height for picking up an imaginary  object and put it on another table at the head height, (e) reaching (reaching an imaginary object in a position above the head).  Tasks (a) to (c) repeated 6 times and tasks (d) to (e) repeated 3 times. Moreover, the Kinect device was set in front of the subjects with 3 m distance and at the height of 69 cm. | (1) Median of joint angles in 3 axes (yaw, pitch, roll; °)  (2) Root-mean-square error (RMSE) of obtained joint  angles in both methods with respect to obtained joint angles by VICON system as the gold standard (°). | (1) Self-developed software and hardware were utilized for obtaining and synchronizing the data of IMUs, Kinect, and motion-capture systems.  (2) Both IMU and Kinect data were up sampled to 100 Hz, to equalize their sampling rate with the motion-capture system.  (3) Xsens-MVN Analyze software package (Xsens Technologies, The Netherlands) generates the IMU-based kinematic model. Through this, segments’ orientation, angular velocity, and angular acceleration, joints’ center position, velocity, and acceleration, and the 3D joint angles were acquired.  (4) A planar projection method was utilized to define the anatomical planes and calculate the joint angles for comparison of measurements of all three methods.  (5) The obtained angles of upper-arm flexion/extension, adduction/abduction, rotation, and shoulder adduction/abduction were used in calculating the sub-scores of the RULA method for each segment at each time sample.  The threshold angle value for not-mentioned segments in RULA was defined 20°.  (6) The agreement between IMU or Kinect and camera-based methods’ RULA scores was assessed by the proportion agreement index and Cohen’s Kappa coefficient (κ). |
| **(3)**  **Lee et al.-2020** | (1) 92 Sample images including 43 different postures were prepared, and the observers mimicked the postures in these randomly ordered sample images displayed on a computer screen.  (2) The other observers performed the conventional posture quantification method (SHARP), by clicking on an appropriate location of the upper extremity on the computer image to identify the postures. | (1) 3 Axes elbow, wrist, forearm, and upper arm joint angles (yaw, pitch, roll; °) | (1) TK Motion Manager software (version 1.0.0; NexGen ergonomics Inc, Pointe Claire, Quebec) calibrates the sensors  (2) Human Motion Analyzer software (version 2.5.0; NexGen Ergonomics Inc, Pointe Claire, Quebec) applies a Kalman filter (NexGen Ergonomics, 2016), and converts the raw IMU sensor data into joint angle measurements (local Cartesian coordinate system)  (3) Each rater/observer classifies raw joint angle values from the selected data set into pre-defined posture categories and reliability is calculated using Fleiss kappa (κ) and Conger weighted kappa (κw) coefficients. |
| **(4)**  **Akanmu, and Olyawela-2020** | (1) Flooring subtasks including sizing and installing wooden floor frames and boards (sensor measurements and video recordings were obtained for 42 min). | (1) Joint angles (yaw, pitch, roll; °)  (2) Duration of carpentry subtasks (s) | (1) Nonrelated subtasks were removed for processing.  (2) Measurements include carpenter’s subtasks, timing of the subtasks, number of cycles and number of data points per cycle.  (3) Based on timings the corresponding joint angles and rotation data for each body segment is extracted.  (4) Raw data are acquired in radians and later converted to degrees.  (5) Using the rotation data, orientation of joints from the neutral plane were obtained.  (6) Risk impositions during each subtask were obtained in terms of percentage of time spent in the awkward posture for each subtask. |
| **(5)**  **Ohlendorf et al.-2020** | (1) 1 Hour of data recording is conducted on each of the four treatment concepts (4 h in total), on a dummy head.  (2) Determined activities of each specialization including, surgical tooth removal, root canal treatment, multiband treatment, and tartar removal are conducted in all four quadrants.  (3) Measurement process of activities is filmed in a total view for differentiating the activities of each individual and synchronization purposes. | (Secondary)  (1) A risk score for every recorded frame using the RULA (a mean score and SD) for wrist and arm  (2) The percentage of spent time in the respective risk scores and at high-risk postures (%) | (1) Synchronization of measurement system and video data is performed by the Xsens software.  (2) MATLAB® vR2018a software (The Mathworks Inc., Natick, MA, USA) is used for the data analysis of the biomechanical measurements.  (3) Both of outcome measurement values for each time sample are combined to obtain upper arm, wrist, and an overall RULA score indicating a level of MSK disorder risk of the activities. |
| **(6)**  **Blume et al.-2021** | (1) The measurement was performed in the “no level” function setting of MVN software since the measurements are not performed on a level floor (the subjects were sitting on a dentist chair).  (2) After calibration, the dental activities were performed on a dummy head using a saddle chair.  (3) The dental tasks included root canal treatment, tartar removal, tooth filling and other tasks (the dental tasks took 4–5 min, except tartar removal that took around 3 min). | (1) Modified RULA score (1 to 7) and relative time spent at each RULA score  [Using accelerometer, angular velocity, and joint angle values in 3 axes (yaw, pitch, roll; °, °/s, g)] | (1) The hip segment was considered as the reference point of the individual´s coordinate system, and the “HD reprocessing” filter setting was applied.  (2) The measurement process was filmed in an all-round view for precise assignments of movements and deviations investigation. the MVN Analyze 2020.2 software (Xsens, Enschede, The Netherlands) synchronized the video data and measurements.  (3) For the evaluation of ergonomic assessment of the posture, RULA score was obtained from the data for each captured image point. |
| **(7)**  **Maurer-Grubinger et al.-2021** | (1) The measurement was performed in the “no level” function setting of MVN software since the measurements are not performed on a level floor (the subjects were sitting on a dentist chair).  (2) The dentists performed a palatinal and marginal incision in region 16 to 11 in the first quadrant for 60 s.  (3) The study focuses on treatment concepts 1 and 2; in treatment 1, the functional area is divided between the dentist and assistant, while in treatment concept 2, dentist and assistant use the same functional area. | (1) Modified RULA score (1 to 7) and relative time spent at each RULA score [Using accelerometer, angular velocity and joint angle values in 3 axes (yaw, pitch, roll; °, °/s, g)] | (1) The hip segment was considered as the reference point of the individual´s coordinate system, and the “HD reprocessing” filter setting was applied.  (2) The measurement process was filmed in an all-round view for precise assignments of movements and deviations investigation. the MVN Analyze 2020.2 software (Xsens, Enschede, The Netherlands) synchronized the video data and measurements.  (3) The angle ranges are mapped to the specific score values of RULA. Therefore, RULA score for each joint can be calculated with respect to time. |
| **(8)**  **Schall et al.-2021** | (1) The data of 15 consecutive weekday shifts of workers were measured.  (2) Calibration includes performing three lateral arms raises from a neutral posture to approximately 90◦ relative to gravity.  (3) During each shift, subjects entered the performing times of work tasks in a diary. The data was used by research team for classification of the work tasks into 6 categories.  (4) Assembly or operating machinery tasks were performed cyclic group subjects. Maintenance or set-up tasks were performed by non-cyclic group subjects. | (1) Upper arms posture or angles (°), and relative time spent at neutral and extreme states (%)  (2) Angular movement speed (°/s) and relative time spent at low-speed and high-speed states (%)  (3) Relative time spent at 3 states of low speed, neutral, and low speed-neutral of rest/recovery exposure (%) | (1) Inclination measurements of the upper arms were acquired through a computationally efficient first-order complementary filter that combined accelerometer and gyroscope data using ActiGraph software. Movement speeds were obtained by the derivative of the upper arm and trunk posture waveforms with respect to time.  (2) The percentage of time in neutral (<20◦) and “extreme” (≥60◦) upper arm posture angles and in low (<5◦/s) and high movement speeds (≥90◦/s) were calculated.  (3) Rest/recovery exposure metrics were defined as the percentage of time in a neutral posture for ≥ 3s, the percentage of time moving at a low speed for ≥ 3s, and the percentage of time in both a neutral posture and moving at a low speed.  (4) The diary information was used to obtain a time-weighted  averages of each posture and movement speed exposure metric by predominant task category. |
| **(9)**  **Merino et al.-2018** | (1) The sensors were calibrated, and subjects performed cutting and removing the bunches from the central stalk in the usual manner for a period of 5 min, of which 4 min and 50 s were synchronized for myoelectric signal acquisition. | (1) Mean joint angles (yaw, pitch, roll; °) and time taken to remove the bunches from the stalk (s)  (2) The maximum voluntary isometric contraction (μV), peak muscle use (RMS and percentage) and mean value of signal and median frequency in each muscle (μV and Hz) | (1) The data were processed in Microsoft Excel.  (2) EMG analysis also involved linear regression of RMS and median frequency values for each muscle.  (3) IMU data were analyzed in Xsens MVN Studio Pro software. |
| **(10)**  **Vignais et al.-2017** | (1) The subjects clean each subpart of the medical material filters, put subparts  together, vacuum the filter, and check the quality of the vacuuming. Afterwards, subjects place each filter inside a packet and seal them (10 work cycles). | (1) RULA score for each joint and an overall score (1 to 7), and percentage of time spent of each joint at risky levels (%)  [Using shoulder, elbow, and wrist joint angles (yaw, pitch, roll; °)] | (1) The electro goniometer data is synchronized with the IMUs data by CAPTIV software (TEA, Nancy, France).  (2) The entire process is filmed with two camcorders.  (3) The joint angles of interest are obtained through a biomechanical model developed by the manufacturer (TEA, 2014).  (4) RULA risk score (ranged 1 to 7) is calculated based on the joint angles at each time step, and an articulation-based analysis was conducted using predefined local scores thresholds. If the risk score of a joint exceeds the threshold the time spent in that posture, or risk score is presented. |
| **(11)**  **Vignais et al.-2013** | (1) Data collection was performed in the SmartFactoryKL living lab that enables conducting tasks in a realistic industrial production environment.  (2) The manual task was composed of (a) turning two hand levers by 90°, (b) removing 4 fuses at knee level and putting them into  a box, (c) taking a screwdriver, unscrewing 4 screws of a transducer’s covering, and putting it down near the box, (d) unscrewing 4 screws of an upper transducer’s covering and putting it down near the box (forces and loads exerted during the task were lower than 4.4 lbs and the standard execution time was below 4 min). | (1) RULA score for each joint and an overall score (1 to 7), and percentage of time spent of each joint at risky levels (%) [Using shoulder, elbow, and wrist joint angles (yaw, pitch, roll; °)] | (1) RULA risk score (ranged 1 to 7) is calculated based on the joint angles at each time step, and an articulation-based analysis was conducted using predefined local scores thresholds. If the overall risk score is 7, an auditory signal is fed to the subject for a period of at least 0.5 s. If a joint’s threshold score exceeds the concerned joint and segment will be highlighted in red through the head mount display of subject. For a RULA score of 5 or 6, the auditory warning is issued after spending at least 5 s in this range. |
| **(12)**  **Zhang et al.-2022** | (1) Calibration was performed by subjects standing in a neutral posture and raising lateral arms to approximately 90◦ relative to gravity (3 repetitions).  (2) Subjects are in two groups of cyclic and non-cyclic tasks. In the “cyclic” group, repetitive and assembly-based production tasks were performed. Subjects in the non-cyclic group mostly perform non-routinized tasks like maintenance, and stocking workstations. | (1) Upper arm elevation angle (°), and the magnitude of the elevation speeds (°/s) | (1) The self-report discomfort, distraction, and burden score of subjects are collected at the end of work shifts (0 to 10, representing the absence to full presence of parameters, respectively).  (2) The inertial data was processed in ActiLife software (ActiGraph, LLC, Pensacola, FL).  (3) 9 Variables were obtained as predictors in linear models: age, sex, BMI (kg/m2), time-weighted average stress ratings, work category (cyclic/non-cyclic), the percentage of time with an arm elevated ≥60◦, right arm in a neutral posture (<20◦) and moving at a low speed (<5◦/s), and the 90th percentile of trunk flexion/extension.  (4) The obtained linear models provide information regarding the discomfort, distraction, and burden scores of using wearable sensors. |
| **(13)**  **Seidel et al.-2021** | (1) At various workplace of subjects, measurements of relevant tasks were performed for 0.5 to almost 5 h per worker. These tasks include (a) shoulder flexion/extension, ab-/adduction, internal/external rotation, (b) elbow flexion/extension, (c) forearm supination/pronation, and (d) wrist flexion/extension, and radial/ulnar deviation. | (1) Mean power frequency of the power spectra of angular data (Hz) [Using 3 axes angular values of measured joints (yaw, pitch, roll; °)]  (2) Mean angular velocity (°/s)  (3) Kinematic micro-pauses (%)  (4) HAL exposure categories (Low/Medium/High)  [Using RMS values of EMG signal (V)] | (1) The updated CUELA-related software acquires the data of CUELA system.  (2) A repetition score is calculated based on the outcomes for wrist and elbow.  (3) Electromyographic data of the forearm flexor/extensor muscles were used to estimate the force component of the TLV for HAL.  (4) By combining the repetition scores and force assessment values, and performing detailed mathematical calculations, a final rate is obtained that is classified in three TLV for HAL exposure categories (Low/Medium/High) |
| **(14)**  **Poitras et al.-2020** | (1) Three maximal voluntary contractions (MVC) were performed for each muscle (anterior and middle deltoids).  (2) An idle standing pose was performed to calibrate the Vicon system and an N-pose (subject standing with 90◦ shoulder abduction).  (3) Simple tasks included shoulder flexion, abduction, scaption (5 repetitions at 3  different joint angles of 60, 90 and 120°)  (4) Complex tasks included lifting a crate from a step, moving it to a specific location on the shelf, taking a 3-s break and returning it back to the starting position (2 repetitions in total, 2 weights of 2.3 and 6.8 kg for 2 shelfs with heights of 1.74 and 1.25 m, 4 weights of 2.3, 6.8, 13.6, and 22.7 kg for a shelf with the height of 0.46 m). | (1) Shoulder ROM and RMSE value with respect to VICON measurement (°)  (2) RMS EMG (V, and (% of MVC) | (1) Collected data of Vicon was processed in the Nexus software (Vicon Motion Systems Ltd., Oxford, UK), and were digitally low-pass filtered at 8 Hz (Butterworth double-pass filter). The Xsens data were obtained from MVN Studio BIOMECH software and imported into MATLAB.  (2) Shoulder joint angles in both systems were calculated relative to the position of the trunk and arm markers and sensors by applying the ZYZ Euler rotation sequence.  (3) EMG signals were processed with a self-developed MATLAB script (digitally filtered with a 4th-order zero-lag Butterworth filter; band-pass 20–450 Hz).  (4) An RMS rectangular window of 20 ms was used for rectifying and smoothing the signal, and the mean RMS value of MVC for each muscle was used to normalize EMGs. |
| **(15)**  **Bassani et al.-2021** | (1) Comparison of proposed EMG system and g.®USBAmp (Guger Technologies): while subject is standing, she holds a 2 Kg mass and flexes her wrist while keeping the arm stretched (8 repetitions).  (2) Comparison of proposed EMG system and shimmer3 EMG: subject squeezes her fist, extends her fingers, and does elbow flexions between 0◦ and 90◦ holding 1.5 Kg mass (3 repetitions).  (3) Comparison of IMU+EMG system with the Vicon (OMG, Oxford, UK) optical motion tracking system: after the calibration, the subject grasps a 2 Kg mass while standing and flexes the wrist four times while keeping her arm stretched. After that she flexes both her shoulders and her elbows and to repeat the task, finally she repeats the four flexions with the arm stretched. | (1) Signal to noise ratio of EMG signals (SNR)  [Using RMS EMG signal values (mV)]  (2) Acceleration, angular velocity, and angle of joints in all 3 axes (yaw, pitch, roll; g, °/s, and °) | (1) A self-developed script in MATLAB provides storing the acquired data and displaying the recorded data.  (2) The power line noise is filtered with a Notch filter and through a 4-th order Butterworth bandpass filter (20 and 500 Hz) sEMG signal is filtered, and finally, they are smoothed, detrended and rectified.  (3) The proposed system has been compared to two other EMG recording systems and Vicon motion tracking system for validation purposes.  (4) The average band power of EMG signal is obtained between 25 and 200 Hz over windows of 256 ms with 99% overlapping, and noise is considered as the inactive states of the muscle. Then, SNR value for each wrist flexion is obtained. |
| **(16)**  **Lee et al.-2019** | (1) 3 Trained ergonomists analyzed joint angles from posture frames using conventional posture observation methods  (2) Subjects mimicked postures in 92 randomly ordered sample images (symmetrical postures of the upper extremities during general material handling activities) displayed on a computer screen (3 repetitions).  (3) The joint angles were measured by a manual goniometer to be used as ground true values in the analysis. | Upper arm joint angles and RMSD values in all 3 axes (yaw, pitch, roll; °) | (1) The TK Motion Manager software (version 1.0.0, NexGen Ergonomics Inc.) obtained calibrated data from IMU system, and the HM Analyzer software (version 2.5.0, NexGen Ergonomics Inc.) converted these data into joint-angle measurements.  (2) Kalman filter as a sensor fusion algorithm allowing compensating the sensor component limitation was applied to compensate for the gyroscope drift in this study.  (3) Joint angles were obtained by I2M IMU system through combining the accelerometer data, orientation of the magnetometer, with the gyroscope data.  (4) Reliability was measured by obtaining intra-class correlation of measured data by 3 trained ergonomists. The validity of the proposed posture-matching method was assessed by estimating the root-mean-square difference between the sensor data and true joint angle of 8 different body postures for each observer. |
| **(17)**  **Peppoloni et al.-2016** | (1) Calibrations include motion tracking system calibration and calibration of the segmentation state flow machine thresholds.  (2) A grocery cashier performs the daily activity including reaching for an object, grasping it, scanning and releasing the object (two check-out operations including 10 items with different weights).  (3) MVC is performed for calculating the ratio of muscle force measured by sEMG. | (1) Shoulder, elbow and wrist extension/flexion, and ulnar deviation (yaw, pitch, roll; °)  (2) RMS values of EMG signal (mV) and power spectral density (W/Hz)  (3) Strain index (SI) and RULA score (1 to 7) | (1) A real-time risk assessment is performed in Self-developed MATLAB GUI based on the Strain Index and RULA score.  (2) In a segmentation state machine, the activity of subjects is categorized (neutral, grasp, reach, and move) based on the shoulder flexion angle and the sum of the RMS of the PSD for all EMG channels.  (3) Based on a 7-DoF model of the human arm (the chest as root) and three links for upper arm, forearm and hand, joint angles, angular velocities, and angular accelerations are estimated from IMU sensors by an Unscented Kalman Filter.  (4) The RULA score computation considers the upper arm flexion, forearm flexion and pronation/supination together with the wrist flexion and abduction. The SI uses both postural (measured from IMUs) and effort scores (measured from EMG sensors). |
| **(18)**  **Battini et al.-2014** | (1) Fashion industry distribution center: operators pick the necessary goods from shelves with 7 different height levels and place them inside shipping box with the selling cases (transportation occurs with big carts). The carts must be kept away from isles and operators go forwards and backwards to pick or to store the required items (the items are hand-carried).  (2) Supermarket warehouse: the operators pick the required items of a picking list, and collect the parts using a cart, carry the cart to a specific zone. | (1) Ergonomic evaluation scores such as RULA, OWAS, OCRA and Lifting Index (LI) [Using shoulders (scapula), upper arms, forearms, and hands  joint angles and postures with 6-DoF (yaw, pitch, roll; °)] | (1) The operators’ movements are real-time assessed based on different ergonomic evaluations such as RULA, OWAS, OCRA and Lifting Index (LI) using three dimensional modeling and the ergonomics indices development with colored measurement scaling through a self-developed software. The software also provides the task types, time, and methods to perform.  (2) Through the provided items in the software, users can select the best suitable method based on the specific limitations of each ergonomics assessment method. Threshold levels are also provided to make alarms if the risk score assessment exceeds those levels. |
| **(19)**  **Slade et al.-2021** | (1) Subjects performed walking for 3 mins on a treadmill at 1.25 m/s.  (2) Subjects performed running for 1 min on a treadmill at 4.0 m/s.  (3) The Fugl-Meyer upper-limb task was simulated by subjects through picking up a knife from a table, performing a cutting motion, and placing the knife back on the table.  (4) All tasks were performed for 10 repetitions. | (1) Shoulder joint angles and RMSE during flexion, adduction, and rotation (yaw, pitch, roll; °)  (2) Elbow joint angle and RMSE during flexion (pitch, °) (3) Wrist joint angle and RMSE during flexion (pitch, °) | (1) OpenSenseRT can support up to 14 IMUs to be sampled.  (2) The software that has been built on the OpenSense tools (OpenSim version 4.2) performs calibration of gyroscopes (averaging gyroscopes data for the first 10 seconds in a neutral pose), and accelerometers (determining the initial orientation of all the IMUs in standing neutral pose of subjects).  (3) The calibrated model and initial pose are applied to the inverse kinematics solver in the software. Microcontroller computes orientations of segments using a Mahony Filter, and another process combines the orientations data and the musculoskeletal model to solve the inverse kinematics. This process results in estimations of joint kinematics at each time step.  (4) The optical motion capture data is applied to the OpenSim software to estimate the validated joint angles and kinematic parameters, and Pearson correlation coefficient of these datasets and proposed system data are calculated to assess the accuracy of the system. |
| **(20)**  **Yang et al.-2020** | (1) Calibration: subjects held an “I-pose” including standing straight, arms hanging close to the body, thumbs pointing forward, and head being neutral.  (2) Surgeries include colorectal, cardiothoracic, general, head and neck,  hepatopancreatobiliary transplantation, neurology, obstetrics, and gynecology, orthopedic, plastic, urology, and vascular. | (1) Mean deviation angle of upper arms (°)  (2) Percentage of spent time in the demanding posture during the surgical time (%) | (1) A self-developed software in MATLAB calculates postural angles for right and left upper arms.  (2) The IMU data is used to define the upper arms orientations relative to the other IMUs, and the segment deviation angles relative to gravity are obtained. The software categorizes postural angles according to a modified RULA and the demanding postures were as >45° for upper arms.  (3) The case duration is based on the scrub-in and scrub-out time and change in fatigue and body part discomfort rating after the operation was defined as the difference of maximum of either during- or post-operation rating and pre-operation rating. |
| **(21)**  **Hallbeck et al.-2020** | (1) The inertial data during 2 NSM cases and two SSM cases for each surgeon was measured.  (2) A researcher attached the IMU sensors on the surgeon and calibrations for movements and postures were performed. | (Secondary)  (1) Orientation (angles) of upper arms using mean angle (yaw, pitch, roll; °)  (2) Spent time in each RULA level for postures during surgery (%) | (1) IMU data were processed and analyzed using a self-developed script in MATLAB.  (2) The segment deviation angles relative to gravity were obtained and were further sorted into levels of a modified Rapid Upper Limb Assessment (RULA) protocol.  (3) The percentage of time spent in each RULA level and an overall risk score for each body part were calculated (scores from 1 to 4; scores higher than 2.5 were considered risky). |
| **(22)**  **Nath et al.-2018** | (1) A cyclic operation including subjects loading a box onto a cart, pushing it to the inspection area, and waiting in the inspection area. If acceptance order is issued, the subject lowers the box onto the cart, pushes it to the unloading area, unloads the box and pulls the cart back to the loading area. If rejection order is issued, the worker pulls the cart back to the loading area (15 repetitions). | (1) Acceleration (g), linear acceleration), and angular velocity in all 3 axes (yaw, pitch, roll; g, m/s^2^, and °/s)  (2) Activity duration (s) and frequency (%) | (1) Jerk or the difference between two consecutive data points, and magnitude of the tri-axial data are obtained, and fixed-length time-windows with 50% overlap are applied to the sensor data.  (2) Statistical features of sensors including the mean, minimum, maximum, standard deviation, skewness, kurtosis, mean absolute deviation, and the 4th-order autoregressive coefficients are collected in each window (12 features per sensor).  (3) SVM method was used for classification in MATLAB® (category 1: lift/lower/carry, category 2: push/pull, category 0: any other no-risk activity).  (4) Based on the duration and frequency of each activity and according to empirical rules of the Occupational Safety and Health Administration, ergonomic risk levels (low, moderate, and high) are estimated. |
| **(23)**  **Jahanbanifar and Akhavian-2018** | (1) The BTE Simulator II simulates construction physical activities including a series of 21 attachments mounted on its exercise head in multiple positions.  (2) 10 Experiments were conducted each of which with a relatively fixed level of power consumed (each lasted for approximately 20 s). | (1) F as the net force exerted (N or kg.m/s^2^)  (2) P as the power (W or kg.m^2^/s^3^)  (3) d as the displacement of the subject’s arm (m)  [Using acceleration, angular velocity and posture values in all 3 axes (yaw, pitch, roll; g, °/s, and °)]  (4) t as the duration of the experiments (s) | (1) Sensor Log smartphone application measured acceleration of subject’s arm.  (2) The experiments were filmed for labeling and cross-referencing of the data and performed experiments.  (3) The outliers and redundant points of raw data were removed in Microsoft Excel with the aid of the recorded video. At last, the data was imported to Python software for the analysis.  (4) Artificial Neural Network (ANN) was applied for developing a model to predict the unseen force levels using the acceleration data. |
| **(24)**  **Cerqueira et al.-2020** | (1) Calibration: subject stand straight back, arms parallel to the trunk, and looking forward (for 10 s), to obtain the offset angle value and subtracted from future measurement.  (2) 5 Sequential tasks in which the first 4 tasks, the two screws must be driven into a robot end-effector at different heights (the neck level, chest level, waist level, and hip level. The 5th task is lifting a 1kg box and placing it at the top of the cabinet (2 repetitions with biofeedback and 2 repetitions without it).  (3) The measured angles are validated using the UR3, a collaborative robot arm from Universal Robots. | (1) Upper arm posture, and RMSE in pitch and roll axes (°)  (2) Time spent percentage during each risk state or posture state (%) | (1) A self-developed graphical interface in MATLAB performs posture state analysis, a risk percentage assessment in a pie plot.  (2) The joint’s orientation in the sagittal and coronal plane are obtained in Euler angles using a Kalman filter to fuse  accelerometer and gyroscope data.  (3) A finite state machine converts an analog angular value to a risk state based on RULA index and complemented with LUBA regarding the coronal plane as thresholds.  (4) Risk levels are Low, Medium, Medium-High, and High along with an allowed time interval to spent in that state. |
| **(25)**  **Singh et al.-2017** | (1) The recordings are made while surgeons perform vaginal procedures on 4 chair types using a 4*4 Latin square model, including a conventional round stool, a round stool with a backrest, a saddle chair with a backrest, and a Capisco chair (the mean recording time of surgery was 122.3 minutes). | (Secondary)  (1) The percentage of time spent in each RULA score for each body part (%) [Using shoulder elevation angle (°)] | (1) The musculoskeletal postural load was sorted into modified RULA risk categories (ranged from 1 to 7).  (2) The calculated percentage of time spent at each RULA score is multiplied by its corresponding risk score. For each body part, a local overall risk score was obtained. |
| **(26)**  **Lind et al.-**  **2020** | (1) Calibration: establishing a neutral position of the upper arm and sending 2 test vibration intensities sequentially to verify proper device wearing  (2) Task: simulated mail sorting task in a  laboratory setting through sorting 30 randomly ordered letters in boxes (7 repetitions) | (1) Angles of upper arm elevation (°)  (2) Proportion of time-spent at upper-arm elevations 30◦, ≥45◦ and ≥60◦ (%) | (1) Data collection and real-time exposure calculation, visual and haptic feedback were included in the self-developed ‘ErgoRiskLogger’ software smartphone application.  (2) The STM32 microcontroller as part of the IMUcore software modules (LP Research, Tokyo, Japan), obtains the quaternion angular positions of IMUs.  (3) If real-time angles of upper arm elevation and work duration exceeds the thresholds of 30° and 60°, a mild and intense vibration actuation is requested to alarm the subject, respectively. |
| **(27)**  **Granzow et al.-2017** | (1) One full-shift day of subjects that included unloading boxes of tree seedlings from a refrigerated trailer, loading seedlings into a bag for planting, and the actual hand planting of the seedlings.  (2) Subjects used a dibble bar with a T-style handle as the primary tree planting tool and carried a bag of seedlings on their back.  (3) For obtaining submaximal isometric reference contraction, in the anterior deltoid, subjects held a 2 kg weight in each hand with upper arms flexed forward to 90◦ of elevation and the elbows fully extended (3 repetitions); subjects performed each contraction for roughly 15s and the mean RMS amplitude of the middle 10s was selected. | (1) Shoulder muscle forces as a percentage of the RMS EMG amplitudes observed for the submaximal reference contractions (%RVE)  (2) Upper arm flexion/extension angles (°) and angular velocity (°/s)  (3) The ratio of time spent at three postures of neutral, rest and extreme, and at two velocities of low and high (%) | (1) A self-developed LabVIEW (version 2013, National Instruments, Inc., Austin, TX, USA) script was used for processing EMG data.  (2) RMS EMG amplitude was obtained using a 100-sample moving window with a 50-sample overlap.  (3) IMUs data were down sampled to 20 Hz using linear interpolation to match the effective sampling rate of the RMS EMG data processing.  (4) Upper arm flexion/extension values were sorted into three postures of neutral, rest and extreme. |
| **(28)**  **Khalil et al.-2021** | (1) Subjects threw 5 fastballs at “maximal effort” state toward a catcher with 18.4 m distance. There was 30s to 60s rest time between each pitch. | (1) Medial elbow torque (N.m)  (2) Arm rotation (maximum angle of the forearm; °),  (3) Arm slot (angle of the forearm in relation to the ground at ball release; °)  (4) Arm speed (maximum rotational velocity of the forearm; rotations/minute) | (1) The Motus Global smartphone application (motusTHROW,  version 8.3.3; Motus Global) processed the data and performed the biomechanical algorithms.  (2) Dynamic ultrasound imaging was utilized for measurements of the ulnar collateral ligament (UCL) and ulnohumeral joint space (UHJS), to assess elbow laxity and further compared with wearable sensor measurements to find the statistical relation. |
| **(29)**  **Villalobos and Maccowlry-2021** | (1) Calibration was performed by asking workers to hold a resting state for the first 10 s of the measurement and remove the offset of data.  (2) The data were obtained during the morning shift of workers from 6 a.m. to 14 p.m with 587 femur deboning actions recorded in total.  (3) For feature extraction, a worker RULA score was measured by an ergonomist during 10 consecutive cuts, in which the ergonomist filled upper body RULA score. | (1) Angle, angular velocity, and acceleration of wrist/hands (yaw, pitch, roll; °, °/s, g)  (2) Wrist/hands RULA score (1 to 4) and the spent time in a risky posture based on RULA score (s) | (1) A self-developed graphical user interface (GUI) was designed for real-time data measurement, risk-factor labeling, and activity visualization.  (2) The processing includes a binary classification algorithm for predicting sharpness/bluntness of the knife, and a multiclass classification algorithm for predicting the value of the wrist/hand RULA score.  (3) Euclidean vector norm across 3 axes was obtained for the acceleration and angular velocity.  (4) A proxy for the exerted power output (normalized by mass) was obtained by calculating the dot product between the tri-axial acceleration vectors and the angular velocities.  (5) 2 Ergonomic features from the data were considered: “over-extension” that counts the number of times during a cut in which a worker passes a high-intensity value for a threshold of acceleration or velocity, and “duration of maximum exertion” that measures the percentage of the time in which the worker is in a risky posture. |
| **(30)**  **Forsman et al.-2021** | (Phase 1): sensors attached to a non-flexible ruler were attached to the right lower arm and hand of the subject as he was performing a manual task, during more than two minutes.  (Phase 2): in a motion tracking lab, three simulated work tasks including, hair drying, folding paper plane and sorting mail were performed to compare their velocity with velocity computed from optical markers. | (1) Wrist angular velocity (yaw, pitch, roll; °/s) | (1) A prototype self-developed smartphone app displays the gyroscope data of each sensor. (2) Subtracting the absolute angular velocity of forearm sensor from the hand sensor velocity leads to the wrist velocity (°/s). |
| **(31)**  **Rodríguez-Vega et al.-2022** | (1) Wrist flexion-extension movements within the range −15° to 15° and movements in a wider range (<−15° and >15°) were performed, and measurements were made when the wrist was pronated, supinated, and in a neutral position.  (2) A goniometer with 1° resolution on the dorsal side of the hand was used for validation of correct hand postures.  (3) Spheric hand grip was performed using five fingers, and with a compressible ball. | (1) Triaxial acceleration, angular velocity, and magnetic field (yaw, pitch, roll; m/s2, rad/s, and µT)  (2) Exerted force each fingertip (described by the voltage) | (1) RULA criteria was the basis of the flexion-extension movement.  (2) Both sensors’ datasets were classified using the Classification Learner application from MATLAB and segmented based on a sliding window with size of 30 observations and step of 10 observations.  (3) The accuracy of 4 classification methods of k-nearest neighbors, support vector machine, decision trees, and Naïve–Bayes algorithms were evaluated. |

(ROM: Range of motion; sEMG: Surface electromyography; V: Volt; W: Watt; s: Second; ms: Millisecond; °: Degree; m: Meter; cm: Centimeter; °c: Degree Celsius; Hz: Hertz; g: g-force [Unit of acceleration]; WMSD: Work-related musculoskeletal disorder; RMSE: Root mean square error; μV: Microvolt; MVC: Maximum voluntary contraction; kg: Kilogram; RMS: Root mean square; N: Newton; T: Tesla; rad: Radian; DoF: Degree of Freedom)

**Table-3: General wearable studies and designs**

| Author- year | Interventions or data collection methods | Measured outcome(s)  [secondary outcomes are indicated with ‘secondary’] | Important points of outcome processing method |
| --- | --- | --- | --- |
| **(1)**  **Hong y et al.- 2021** | (1) Bandings caused by joint movements are translated into electrical signals using piezoelectric sensors, then they are processed and recorded during a 100-s moving trial. | (1) Bending angle (°) (2) Bending radius (mm)  (2) Voltage recorded from sensors movement (mV) | (1) Self-developed software processes the sensor parameters data.  (2) The Finite Element Analysis (FEA) is applied through COMSOL Multiphysics version 5.5 and obtains the mechanical and electrical responses of HAPNC sensor, and based on FEA, the deformation modes correspond to different strain states. |
| **(2)**  **Jang et al.-2020** | (1) 12 Classes were defined including three conditions corresponding to elevated shoulders, i.e., symmetric shoulders, higher right shoulder, or higher left shoulder and to train the deep leaning algorithms.  (2) Subjects performed 6 actions for 1 min each. The actions included moving neck forward and backward while holding different shoulder positions (symmetric/asymmetric and rounded/unrounded). | (1) The shoulder symmetry angle (SA) represented by percentage (%)  [Using elevation angle of shoulders (°)] | (1) The angles between a horizontal line and the line connecting the C7 and acromion are obtained by the IMU sensors placed on shoulders and neck.  (2) Through simple math equations and two machine learning methods of SVM and KNN and two deep learning algorithms of DNN and CNN, the SA values were obtained and compared. A SA value less than −10% or more than 10% is regarded as a subject has elevated his/her shoulders. |
| **(3)**  **Matiur Rahman et al.-2021** | (1) The elbow angles (0°, 30°, 60°, 90° and 120°) were measured with a goniometer, and static contractions with maximum force (MVC) were performed by subjects at each angle for 5s durations (3 repetitions). | (1) sEMG signal (V) | (1) K nearest neighbors (k-NN) was applied to build a model for determining the EMG characteristics and discriminating between  elbow joint angles.  (2) 15 Time-domain features were extracted from sEMG signals including:  a. Mean absolute value  b. Standard deviation (STD)  c. Root mean square (RMS)  d. Zero crossing (ZC)  e. Simple square integral  f. Variance (VAR)  g. Integrated EMG (IEMG)  h. Mean peak value (Peak)  i. Energy (EN)  j. Skewness (SK)  k. Coefficient of variation  l. Mean absolute deviation  m. Wavelength (WL)  n. Average amplitude change  o. Difference absolute standard deviation value |
| **(4)**  **Zabat et al.-2015** | (1) Accelerometer calibration is performed using a 360° protractor associated with a bubble level and for magnetometer is performed by a 360° rotation of the sensor in horizontal plane and draw Y-axis magnetic component versus X-axis magnetic.  (2) The subject performs a maximal shoulder flexion-extension. | (1) Upper arm joint angles (yaw, pitch, and roll; °) | (1) Self-developed GUI designed with Microsoft Visual C# environment with .NET Framework presents a 3D real-time movement animation, measured angles and recording the data in an Excel file.  (2) The accelerometer measures the angles in a vertical plane (flexion/extension, and abduction/adduction) while the magnetometer performs the correct measure in a horizontal plane (internal/external rotations). |
| **(5)**  **Romero avilla et al.-2020** | (1) The sEMG signals were recorded in a resting state for 20 s to determine and remove the noise level of the sEMG sensor system.  (2) The subjects were strapped to a pulley machine (5 kg load) such that the elbow axis aligned with the central axis of a deflection pulley.  (3) Subjects performed an elbow flexion/extension movement with self-selected movement velocity (5 repetitions). | (1) RMS values EMG signal (mV) | (1) Both systems sEMG signals were band pass filtered (Butterworth, ninth order, 20–500 Hz), and the envelope of the sEMG signal was obtained after full wave rectifying and getting smoothed with a moving average 80 ms sliding window.  (2) The signal envelopes are divided into 80-ms epochs, and the RMS value of each epoch was obtained.  (3) Mean plus 10-fold standard deviation of the sEMG envelope was defined as a threshold of the phases of muscular activation.  (4) A reference sEMG system (Noraxon USA, Inc. Scottsdale, AZ, USA; sampling frequency of 1500 Hz) measured the sEMG signals for comparison purposes. |
| **(6)**  **Jurioli et al.-**  **2020** | (1) The device is evaluated through solving a VR jigsaw puzzle of a smartphone application (the settings of puzzle like sensitivity, number of pieces, and other information can be modified by a therapist).  (2) Tests for delay, motion sickness and level of sensitivity of VR jigsaw puzzle were performed by subjects, and their subjective scores were obtained through questionnaires. | (1) Wrist positions in relation to the Elbow (yaw, and roll, °)  (2) Time spent for completing the puzzle (s) | (1) After data processing, the outcome values are sent to the self-developed smartphone application allowing to control the VR environment and further analysis of therapist.  (2) The software considers one patient arm being used in the VR environment. |
| **(7)**  **Elshafei and**  **Sheihab-2021** | (1) Subjects pick up a specific dumbbell placing the right elbow on the top of the inner right thigh and pull up the dumbbell by only moving the forearms and contracting the biceps while breathing out. The dumbbell must be pulled up to shoulder level. The position is held for 1 s and then the dumbbell is slowly brought back (5 sets, and 15 repetitions in each of the sets are performed). | (1) X-axis and Z-axis angular velocity and posture (°/s, °), Y-axis of accelerometer (g)  (2) Total exerted force of hand (calculated by product of dumbbell mass and acceleration; N) | (1) After five sets of concentration curls, a rate of perceived exertion (RPE) value evaluated by subjective value of Borg scale is reported by the volunteers to identify fatigue existence in a repetition.  (2) Machine learning methods for classification models is used to extract 33 features for detection of bicep muscle fatigue in concentration curl repetitions. |
| **(8)**  **Karunarathne and Pathirana-2014** | (1) An arm exercise is performed with a low acceleration compared to gravity by lifting a bottle from the front of subject body to his/her mouth. | (1) The Root Mean Square Error (RMSE) of measured angles in both methods of IMUs and VICON motion capture (°)  [Using wrist and elbow joint angles, accelerations, and velocities (yaw, pitch, roll; °, °/s, g)] | (1) 8 Solutions for Wahba’s problem are considered and root mean square error derived from comparison of the IMUs measurements and the VICON optical motion capture system is assessed. The 8 solutions are High Pass Filter – Gyroscope,  Low Pass Filter – Accelerations, Traditional Complementary Filter, Adaptive Complementary Filter, TRIAD Method, Davenport’s q Method, Singular Value Decomposition Method, and QUEST Method. |
| **(9)**  **Young et al.-2021** | (1) Subjects performed a dynamic range of motion trial for both Flexion/Extension (FE) and Radial/Ulnar deviation axes.  (2) Subjects held their static wrist angles by placing their forearm on an armrest affixed to the desk and increased in 10-degree increments shown by a protractor on the desk. | (1) Wrist flexion/extension and radial/ulnar deviation angles (pitch, yaw; °) | (1) The data analyzing was made through self-developed Python 3.9 script.  (2) Wrist angles were calculated using Euler angles with a ZYX rotation order (Z-axis defined FE and the X-axis defined RUD).  (3) Joint angles were calculated based on the ISB recommendations for the wrist joint coordinate system.  (4) All joint angle time series were low pass filtered (4th order zero lag Butterworth filter; cut-off frequency=10Hz). |
| **(10)**  **Hochman et al.-2020** | (1) Subjects move their wrist according to the two exercises at the desired movement speed (2 s cycle period) through provided animations (10 cycles of exercises, three times for each combination of unweighted flexion-extension and rotation exercises at two sets of microphone location for both wrists).  (2) The forearm was held stationary by strapping it to the arm of the Subject’s chair. The subjects hold the grip containing IMU and press the accelerometer onto the skin of the palm while also constraining fingers. | (1) Signal-to-noise ratio (db) of recorded signal of microphones (uniaxial-accelerometer)  (2) Wrist angle and acceleration in 3 axes (yaw, pitch, roll; °) | (1) The acoustic data were bandpass filtered (150 Hz–20 kHz) and processed through a self-developed MATLAB script.  (2) The signal power of click windows (± 50 ms of each detected click) were obtained and divided to signal power of non-click windows as noise.  (3) 9 Audio features were extracted, and intraclass correlation coefficient (ICC) coefficients of variability (CVs), and Jensen–Shannon (JS) divergence were acquired for evaluation of the interrater repeatability of the signals. |
| **(11)**  **Saito et al.-2017** | (1) Elbow movements are made after embedding the pyrolytic graphite sheet (PGS) sensor in a rubber glove.  (2) Finger movements are made after embedding the PGS sensor in an ultrathin glove over the middle finger joint. | (1) Resistance of the strain sensor (Ω) | (1) Through movements of the joint the resistance of the PGS strain sensor changes. Thus, by performing measurements of resistance change, joint motions can be detected.  (2) The proposed sensor can be applied to monitor health care of patients and even as controllers for virtual games. |
| **(12)**  **Xie et al.-2020** | (1) Experiments of index finger bending include bending slowly for ∼25 s, then bending quickly for ∼10 s, holding for ∼10 s, and quickly bending for ∼5 s, and holding for ∼10 s (total recording duration is ∼1 minute). Another experiment was random finger bending total recording duration is ∼90 s.  (2) Experiments of wrist bending include non-bending for ∼5 s, and then bending up/down for ∼5 s (total recording duration is ∼35 s). | (1) Finger and wrist bending level and frequency through EM impedance (Ω) | (1) By performing bending, the distance between the EM coils becomes different. Therefore, the mutual impedance signal will  be different.  (2) The obtained signal is around 0 Ω during full bending and reaches to a peak during non-bending state.  (3) Four finger-bending status of non-bending, slight bending, middle-level bending, and full bending can be detected. |
| **(13)**  **Smondrk et al.-2021** | (1) The bend sensors’ electric resistance is measured using the developed calibration equipment utilizing the goniometer. The sensor was bent from 0° to 110° with a step of 5° and the resistance and corresponding voltage is recorded.  (2) Experimental reliability is assessed by a subject bending each finger at full range (3 repetitions) and making a fist by flexing the fingers (5 repetitions). | (1) Fingers joint angles (pitch, °) | (1) Through a self-developed electrical circuit, the change in the resistance value of the bend sensor is transformed into voltage.  (2) The voltage is amplified through a mathematical equation the recorded voltages are translated into angles. |
| **(14)**  **Zheng et al.-2016** | (1) The validity was evaluated by comparing the sensor-measured values and traditional measurement values (goniometer and weights).  (2) Reliability was evaluated through different tests including gripping a mold, flexing fingers, and lifting a salver.  (3) Using ‘‘resample” procedure, the time courses of joint bending angles recorded by FuncAssess glove were processed, and the inter-joint relationships abnormality is identified by comparing the resample results of abnormal motion modes with the normal motion modes. | (1) Finger joints bending angle (°)  (2) Finger joints load (gram) | (1) A self-developed MATLAB software was designed for receiving, presenting, and storing the data.  (2) The received voltage data were translated to angle values through a look-up table at 0.1° intervals generated by a shape-preserving interpolation function, “pchip” in MATLAB. |
| **(15)**  **Moreira et al.-2014** | (1) After performing an initial calibration of sensors, reference for IMUs is determined by the current orientation of each IMU at 10 seconds after the gyroscope offset calibration.  (2) Technical tests were performed to evaluate the stability, accuracy, and consistency of the sensor-measured angles.  (3) Repeatability of the finger bending trajectories during performing four daily tasks of closing/opening fist, counting with fingers, picking up a glass and releasing it, and moving a mouse/pressing a click (all for 5 repetitions) and calculating intra-class correlations between the repetitions. | (1) Fingers and hand joint angles (yaw, pitch, and roll; °) | (1) A python-developed script of Blender 3D software presents a 3D hand model with 17 bones.  (2) Using the quaternions mathematic equations and combining the data of 3 sensors of IMUs, the localized joint orientation quaternions of each IMU and therefore, each joint is obtained. |
| **(16)**  **Hazman et al.-2020** | (1) Relax state (as calibration) is measured initially, and fully flexion of fingers are performed by subjects (3 repetitions), and the average value is tabulated. | (1) ROM of the MCP, DIP, PIP joints in angles (yaw, pitch, and roll; °)  (2) Percentage of error between both methods (%) | (1) A self-developed GUI on MATLAB monitors the ROM of every finger joint for data analysis and presents a 3D visualization of a rectangle object representing finger.  (2) Goniometry is the validated reference for the accuracy of the IMU and bend sensors measurements. |
| **(17)**  **Oigawa et al.-2021** | (1) Subjects gripped and released their hands under the states of free, fast, slow, irregular, shivering, and with/without moving a specific finger.  (2) Grip-and-release motions were performed by both hands for 20 s each (sampling frequency = 200 Hz). | (1) 3 Axes, and total absolute acceleration (yaw, pitch, roll; g)  (2) Contact force (N) | (1) The HapLog software performed the data collection and processing tasks.  (2) Video recordings were made during measurements.  (3) 10s of data (2048 data points) were obtained from a stable grip-and-release motion after the first 5 s of start point.  (4) Data variation, number grip and releases, frequency characteristics, and correlation of each finger were obtained for further evaluation. |
| **(18)**  **Rovini et al.-2020** | (1) While sitting, subjects put their hands on a table in a relaxed position. After hearing a tone, the tasks must be performed (10 repetitions):  (a) Finger tapping (tapping forefinger against the desk with self-pace speed)  (b) Thumb-forefinger tapping (subjects lean the forearm on the desk, with the thumb in contact with the forefinger and tap his/her forefinger against his thumb)  (c) Drink (reaching and grasping a bottle with distance of 21 cm on the table and moving it toward the mouth)  (4) Individual (reaching and grasping a bottle with distance of 21 cm on the table and moving it toward the target position located 28 cm at the right side of bottle initial position)  (5) Social (reaching and grasping a bottle with distance of 21 cm on the table and passing it to a partner at the right side) | (1) Triaxial accelerations and  angular velocities (yaw, pitch, roll; g, °/s)  (2) Number of repetitions, frequency of the movement (Hz), and range of index finger movement (°) | (1) Vicon Nexus 2 software real time processed and collected the data.  (2) SensRing processes the triaxial accelerations and angular velocities with a 4th-order low-pass digital Butterworth filter (cut-off=5 Hz) for eliminating the high-frequency noise.  (3) The data were segmented and processed with self-developed algorithms to extract: (a) number of repetitions, (b) frequency of the movement, and (c) amplitude of the movement of the index finger.  (4) The absolute error (difference between VICON data and SenseRing data) on the average data was calculated for the three mentioned parameters. |

(ROM: Range of motion; sEMG: Surface electromyography; V: Volt; s: Second; ms: Millisecond; °: Degree; °c: Degree Celsius; Hz: Hertz; g: g-force [Unit of acceleration]; RMSE: Root mean square error; RMS: Root mean square; VR: Virtual reality; SVM: Support vector machine; KNN: K-nearest-neighbors; CNN: Convolutional neural network; DNN: Deep neural network)
